# Expedient discovery of fluorogenic amino acid-based probes *via* one-pot palladium-catalysed arylation of tyrosine†

**DOI:** 10.1039/d5sc00020c

**Published:** 2025-01-23

**Authors:** Olivia Marshall, Rochelle McGrory, Sineenard Songsri, Andrew R. Thomson, Andrew Sutherland

**Affiliations:** a School of Chemistry, University of Glasgow Joseph Black Building, University Avenue Glasgow G12 8QQ UK Andrew.Sutherland@glasgow.ac.uk

## Abstract

To overcome the limitations of using large extrinsic chromophores for biological imaging, fluorescent unnatural α-amino acids have been widely adopted as intrinsic peptidic probes. Although various classes have been successfully utilised for imaging applications, novel amino acid probes readily prepared through operationally simple synthetic methodology are still required. Here, we report a new approach for the synthesis of unnatural α-amino acids *via* a one-pot process involving activation and palladium-catalysed arylation of tyrosine. Rapid access to a small library of novel α-amino acids has allowed the discovery of a dimethylaminobiphenyl analogue that displays strong charge transfer-based fluorescent properties and is both solvatochromic and pH sensitive with a significant hypsochromic shift in emission under acidic conditions. The imaging potential of the dimethylaminobiphenyl α-amino acid was demonstrated *via* its application as a FRET donor in a novel decapeptide substrate for monitoring and evaluating the kinetics of a serine protease.

## Abstract 2

A one-pot palladium-catalysed arylation process has been used to expedite the synthesis of biaryl α-amino acids, leading to the discovery of an environment-sensitive fluorescent probe that was used to monitor the activity of a serine protease.

## Graphical abstract

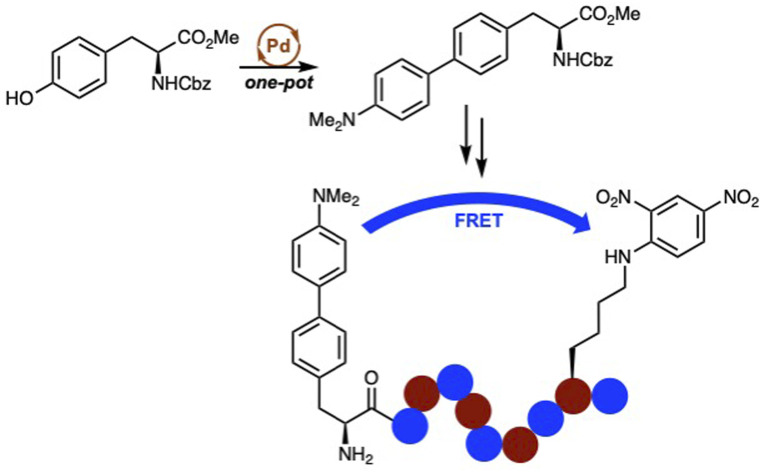

## Introduction

Fluorescent spectroscopy is a key technique for studying biological processes.^1^ The high detection sensitivity down to single-molecule level and real-time monitoring along with variable time scales have allowed its application for the non-invasive imaging of various events in biomedical and life sciences research.^2^ However, enabling the full potential of fluorescent spectroscopy requires the development of minimally invasive fluorophores that can report without interrupting the biological process.^3^ For imaging with proteins and peptides, large chromophores with appropriate photophysical properties are typically attached at the terminus using a chemical spacer.^4^ However, this can still disrupt the structure and function of the peptide and, positioning of the chromophore at the terminus of a protein limits applications.

**Fig. 1:**
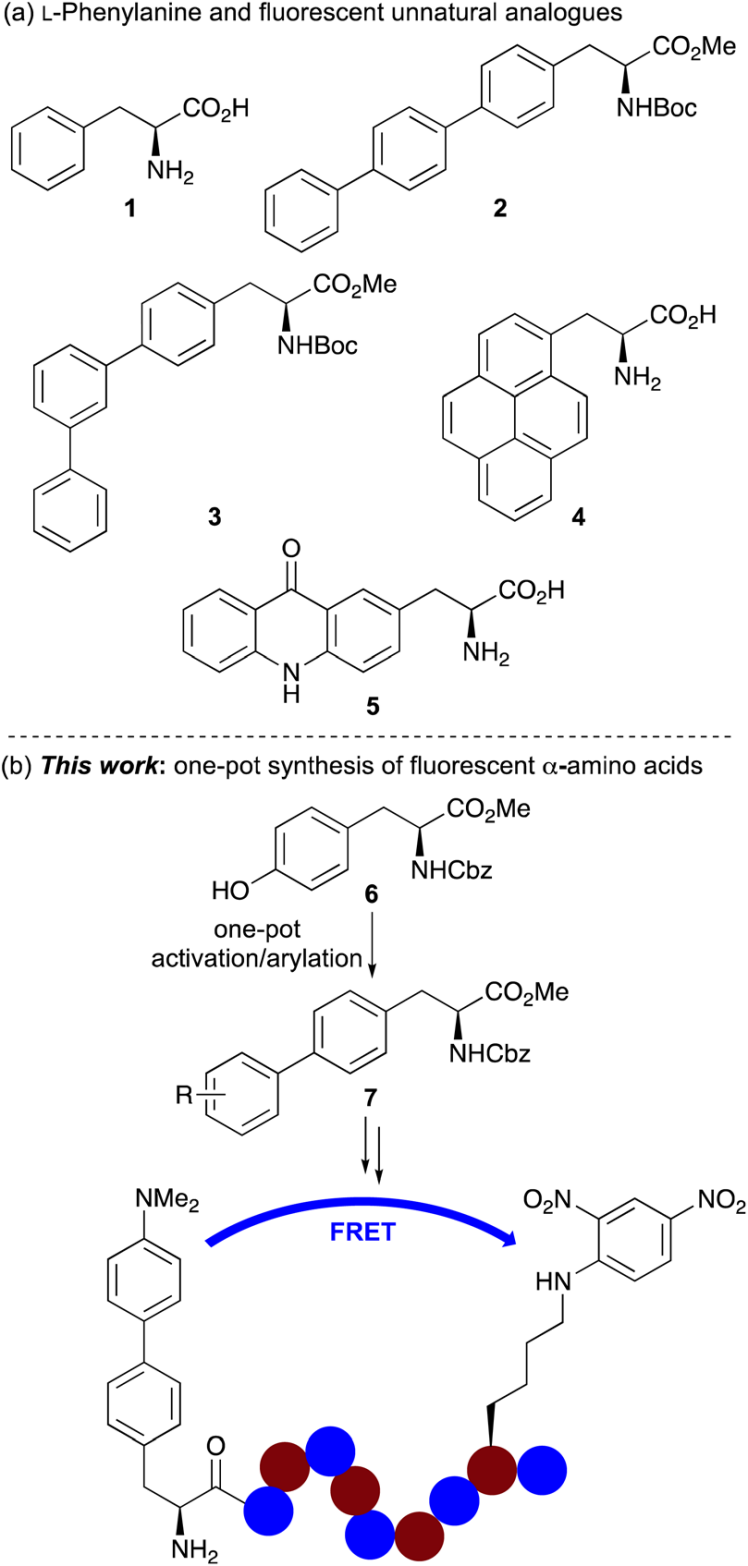
(a) l-Phenylalanine (1) and selected fluorescent analogues. (b) One-pot synthesis of biaryl α-amino acids and application as an internally quenched fluorescent peptide.

The issues associated with the use of large extrinsic chromophores for peptide imaging have resulted in the development of fluorescent unnatural α-amino acids as probes for biological imaging.^5^ Chromophores with specific photophysical properties can be incorporated into the amino acid side chain, and the amino acid probe can be selectively embedded into the protein structure using either solid phase peptide synthesis (SPPS) or genetic encoding. A key approach for the design of novel fluorescent amino acids involves the modification of fluorescent proteinogenic α-amino acids. Although efforts have primarily focused on the development of structural analogues of the most fluorescent proteinogenic α-amino acid, tryptophan,^6–8^ analogues of phenylalanine (1) have also been reported (Fig. 1a).^5^ These include a number of polyaromatic analogues such as 4-biphenyl-l-phenylalanine 2, which was incorporated into dihydrofolate reductase (DHFR) *via* genetic encoding and used in a Förster resonance energy transfer (FRET) experiment to study conformation change following inhibitor binding.^9^ Other terphenyl α-amino acids such as 3 and prenyl-l-alanine (4) have also been introduced into DHFR and used to investigate protein dynamics.^10,11^ Acridon-2-yl-l-alanine (5), prepared in five steps from l-tyrosine has been shown to possess a long fluorescent lifetime and emit in the visible region.^12^ These properties have allowed the use of this fluorescent unnatural α-amino acid for monitoring protein folding and binding interactions using FRET assays with common fluorophores such as coumarins.^12*b*^ More recently, the Ackermann and Vendrell groups have reported the synthesis of phenylalanine-BODIPY conjugates that have been used to detect *Candida* infections in human urine samples.^13^

Despite the significant progress in identifying fluorescent polyaromatic α-amino acids for biological applications, novel analogues with minimally invasive side chains, which can be readily incorporated into peptides using standard techniques for new imaging experiments are still required. In addition, synthetic methodology that allows rapid access to libraries of novel α-amino acid analogues which can expedite the discovery of new probes is of particular importance. As part of a research programme focused on the development of fluorescent unnatural α-amino acids,^8,14^ we were interested in exploring new analogues of phenylalanine for the development of a fluorophore that could be used in FRET experiments to monitor protease activity. Here, we report the development of a one-pot synthesis of biaryl α-amino acids by activation of a commercially available tyrosine derivative, followed by palladium-catalysed arylation that allows rapid access to novel fluorescent phenylalanine analogues (Fig. 1b). We also describe how straightforward access to these compounds has resulted in the discovery of several α-amino acids with strong fluorescence, including an environmentally sensitive dimethylaminobiphenyl analogue, that is highly compatible with SPPS and an effective FRET donor for determining the enzyme kinetics of serine proteases.

## Results and discussion

The first objective of this research programme was the development of a one-pot procedure for the preparation of biaryl α-amino acids from tyrosine derivative 6. Although, various methods for the one-pot activation and arylation of simple phenols using transition metal catalysis are known,^15^ to the best of our knowledge the use of α-amino acid substrates has not been reported. Aryl nonafluorobutylsulfonates (aryl nonaflates, ArONf), which are readily prepared from phenols and commercially available perfluoro-1-butanesulfonyl fluoride are increasingly used for metal-catalysed cross-coupling reactions.^16^ This is due to the ease of synthesis and purification, as well as their high reactivity compared to other aryl sulfonates. Akai and co-workers have shown these to be useful intermediates during the one-pot conversion of phenols to substituted aryl compounds using Suzuki–Miyaura, Sonogashira and Stille reactions.^15*a*^ Based on this work, the use of a nonaflate intermediate of tyrosine 6 for subsequent arylation was investigated (Table 1). Under standard conditions, tyrosine 6 was converted to the corresponding nonaflate derivative with complete conversion as shown by ^1^H NMR spectroscopy. On formation of the nonaflate, Suzuki–Miyaura cross-coupling using phenylboronic acid was initially attempted using the optimised conditions described by Akai and co-workers,^15*a*^ involving Pd_2_(dba)_3_ (1 mol%) and the ligand, SPhos (2 mol%, entry 1). However, this gave biphenyl α-amino acid 7a in only 30% yield. To improve the cross-coupling step, the use of the Buchwald precatalyst XPhos Pd G2 was explored.^17^ At 1 mol% loading and using Cs_2_CO_3_ gave no conversion (entry 2), however, the use of K_3_PO_4_ as the base gave 7a in 46% yield (entry 3). Further improvement was achieved with the addition of water as a co-solvent (entry 4), which in combination with the base is known to facilitate transmetallation and reductive elimination.^18^ This resulted in the isolation of 7a in 66% yield. At this stage, further optimisation studies showed that a reaction time of 20 h and a temperature of 60 °C were necessary for high conversion.^19^ Further analysis by ^1^H NMR spectroscopy of the cross-coupling step showed that under standard conditions (entry 4), the reaction was incomplete. Higher catalyst loading (2 mol%) gave a slight increase in yield to 71% (entry 5), however, nonaflate intermediate was still observed in the reaction mixture. To ensure complete conversion during the second step, batch addition of catalyst was investigated. After some optimisation, it was found the addition of three batches of catalyst (3 × 1 mol%) during the reaction resulted in complete conversion of the nonaflate and the isolation of 7a in 91% yield (entry 6).

**Table 1:** Optimisation of the one-pot activation and Suzuki–Miyaura cross-coupling reaction of tyrosine derivative 6

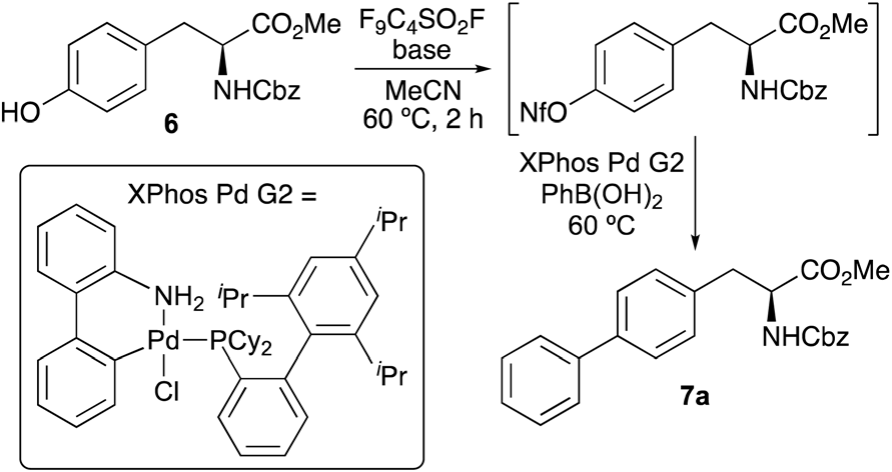				
Entry	Catalyst loading (mol%)	Base	Time (h)	Yield a(%)
1^b^	1	Cs_2_CO_3_	18	30
2	1	Cs_2_CO_3_	18	—
3	1	K_3_PO_4_	18	46
4^c^	1	K_3_PO_4_	20	66
5^c^	2	K_3_PO_4_	20	71
6^c^	3 × 1	K_3_PO_4_	20	91

a Isolated yield.

b Reaction was done using Pd_2_(dba)_3_ (1 mol%) and SPhos (2 mol%).

c Water was added as a co-solvent for the second step.

**Scheme 1:**
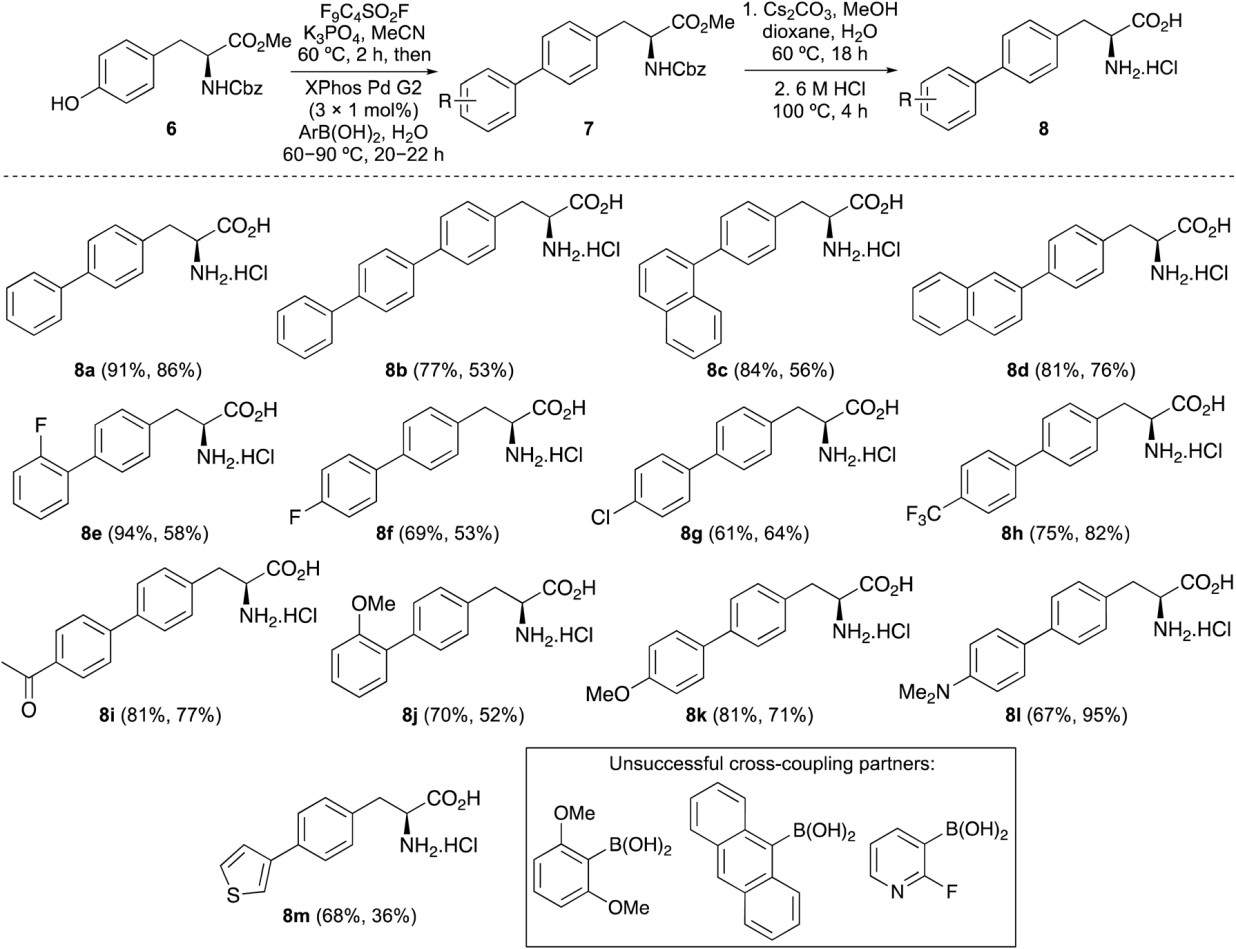
Three-pot synthesis of α-amino acids 8a–8m. The parentheses underneath each structure show the yield for the one-pot coupling followed by the overall yield for the two-step deprotection.

On optimisation of the one-pot activation and Suzuki–Miyaura cross-coupling reaction of tyrosine 6, the scope of the process was explored with a range of aryl boronic acids (Scheme 1). Using polyaromatic or phenyl substituted boronic acids bearing either electron-rich or electron-deficient substituents gave the products in good to excellent yields. The use of *ortho*-substituted aryl boronic acids was also effective, forming 7e and 7j in 94% and 70% yields, respectively. Heteroaromatic boronic acids were also tolerated with the synthesis of thiophene analogue 7m in 68% yield. Unsuccessful cross-coupling reactions were only observed with sterically hindered *ortho*-disubstituted boronic acids or very electron-deficient partners such as 2-fluoropyridin-3-yl boronic acid. Previous syntheses of polyaromatic α-amino acids such as 4-biphenyl-l-phenylalanine 2, used for imaging of DHFR,^9^ have typically been prepared using a four-step approach involving halogenation of phenylalanine using sodium iodate and concentrated sulfuric acid,^20^ incorporation of protecting groups and then the Suzuki–Miyaura cross-coupling step.^9*a*,21,22^ The strategy described here for the synthesis of similar compounds (*e.g.*7b) avoids both harsh conditions and multi-step routes, with one-pot access from commercially available tyrosine derivative 6.

Following the preparation of a series of analogues, these were deprotected in two steps to the parent α-amino acids (Scheme 1). Ester hydrolysis using caesium carbonate was followed by removal of the Cbz-protecting group under aqueous acidic conditions. Purification by recrystallisation gave α-amino acids 8a–8m in 36–95% yields over the two steps. Overall, the strategy of a general one-pot arylation process using a readily available tyrosine derivative allowed the expedient preparation of a range of novel α-amino acids for photophysical analysis.

**Fig. 2:**
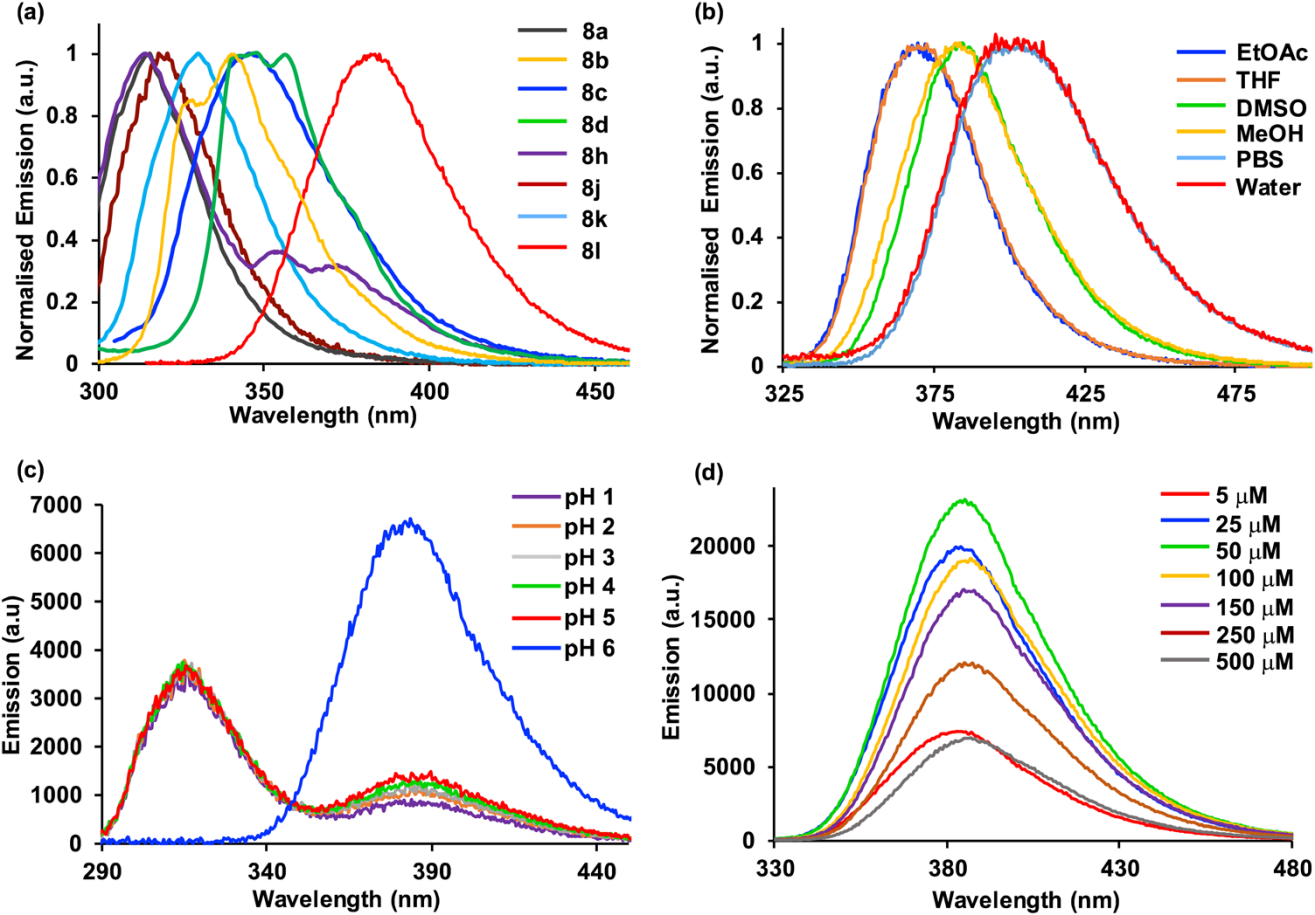
(a) Normalised emission spectra of selected amino acids in methanol at 1–5 μM (excitation was typically performed at absorption maxima). (b) Normalised emission spectra of 8l at 5 μM in various solvents. (c) Emission spectra of 8l at 5 μM at various pH. (d) Emission spectra of 8l at various concentrations in methanol.

The UV/visible absorption and photoluminescence spectra of α-amino acids 8a–8m were then measured (ESI†). As expected, the majority of compounds possessed red-shifted absorption and emission spectra compared to l-phenylalanine (1). In addition, screening revealed that α-amino acids with electron-deficient aryl substituents were found to possess the weakest fluorescent properties with emission from locally excited states (*e.g.*8e–8i), while those with polyaromatic side chains or electron-rich substituents displayed the brightest emission *via* internal charge transfer states (Table 2 and Fig. 2a). Amino acids 8b, 8d and 8j were found to have interesting properties with brightness >5000 cm^−1^ M^−1^, however, 4-dimethylaminophenyl analogue 8l was found to possess the most impressive overall properties. This included absorption and emission maxima at 300 and 384 nm, respectively, a large Stokes shift, a quantum yield of 0.73 and the strongest brightness of 12 460 cm^−1^ M^−1^. With absorption and emission properties at longer wavelengths and stronger brightness than 8b, which has previously been used for biological imaging,^9^ and a higher quantum yield than other rigid, polyaromatic phenylalanines,^11*b*^ it was proposed that 8l could be developed as a new fluorescent amino acid probe.

**Table 2:** Photophysical data for l-phenylalanine and selected α-amino acids^a^

Amino acid	*λ*_Abs_ (nm)	*ε* (cm^−1^ M^−1^)	*λ*_Em_ (nm)	*Φ*_F_ b	Brightness (cm^−1^ M^−1^)
l-Phe^3*a*^	258	200	282	0.024	5
8a	254	24 200	314	0.12	2930
8b	284	14 100	341	0.83	11 740
8c	282	10 300	342	0.27	2800
8d	289	30 900	356	0.18	5562
8h	262	23 300	312	0.15	3490
8j	288	13 800	333	0.38	5240
8k	262	14 100	328	0.24	3430
8l	300	17 000	384	0.73	12 460

a All spectra were recorded at 1–5 μM in MeOH.

b Quantum yields (*Φ*_F_) were determined in MeOH using l-tryptophan as the standard.

**Fig. 3:**
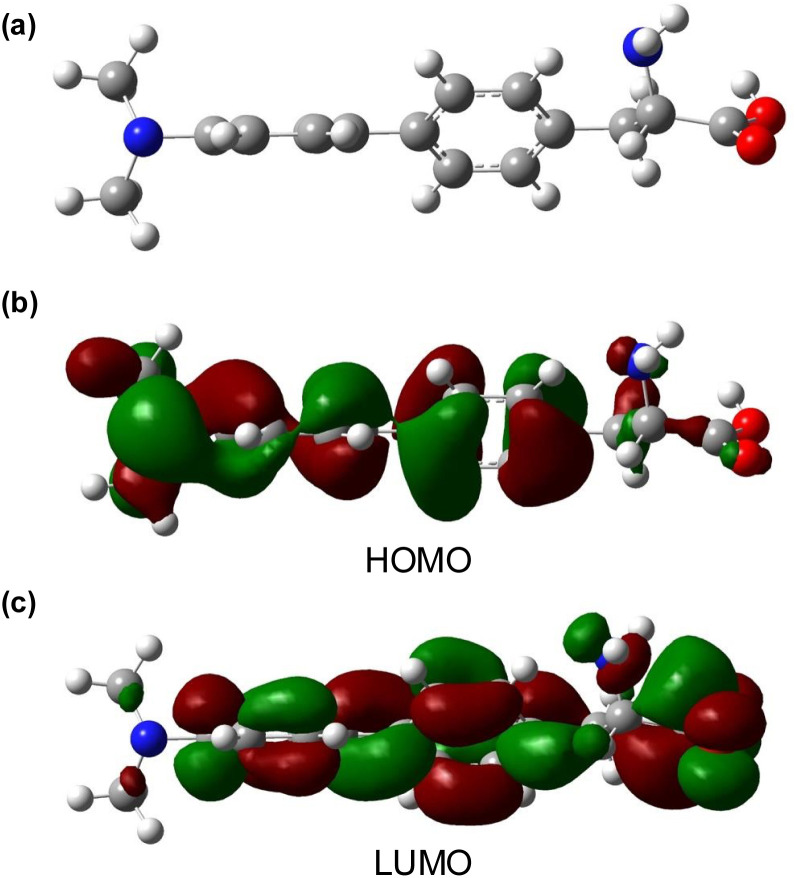
(a) Energy minimised DFT structure of 8l using B3LYP/6-31G basis sets. (b) Calculated HOMO isosurface plot from the DFT model. (c) Calculated LUMO isosurface plot from the DFT model.

To further assess amino acid 8l as an imaging agent, solvatochromic and pH studies were performed. In various solvents, a bathochromic shift in emission was observed on increasing solvent polarity (Fig. 2b). For example, an emission maximum of 370 nm was recorded in ethyl acetate, with a shift to 404 nm in water. This confirms the intramolecular charge-transfer character of the excited state of 8l, which is stabilised in more polar solvents. The solvatochromism of amino acid 8l was further verified by a Lippert–Mataga plot (ESI†), which showed a linear relationship between the Stokes shifts and solvent orientation polarisability.^23^ The photophysical properties of amino acid 8l were found to be highly sensitive to pH. Acidification from pH 6 to pH 1 resulted in a hypsochromic shift of the main bands in both absorption and emission spectra. The p*K*_a_H of dimethylaminobenzene is 5.06 and thus, the dimethylamino group of 8l is expected to be protonated at pH < 5.^24^ Thus, under acidic conditions, charge transfer is effectively turned off (8-fold decrease in CT band at 384 nm) and emission occurs from a locally excited state (315 nm, Fig. 2c) in the same manner as unsubstituted biphenyl amino acid 8a. At neutral or basic pH, when the dimethylamino group is not protonated, strong charge transfer fluorescence at 384 nm is retained (ESI†). Studies to investigate the effect of viscosity and aggregation with amino acid 8l were also conducted. Both the absorbance and emission of 8l were independent to changes in viscosity [100% MeOH (*η* = 0.59 mPa s) to 100% ethylene glycol (*η* = 13.5 mPa s)],^25^ while aggregation studies showed increased emission up to 50 μM, before a further increase in concentration (up to 500 μM) resulted in aggregation induced quenching (Fig. 2d). To further probe the charge transfer properties of 8l, which is directly responsible for the polarity and pH sensitivity, DFT calculations were performed. Initially, an energy minimised model of 8l was optimised using the B3LYP/6-31G basis sets,^26^ which confirmed the expected twisted conformation of the biphenyl side chain (Fig. 3a). Frontier molecular orbital analysis at the same level of theory allowed visualisation of the highest occupied molecular orbital (HOMO) and lowest unoccupied molecular orbital (LUMO) states. The largest contribution of electron density of the HOMO (Fig. 3b) was found localised on the electron-rich 4-dimethylamino-substituent, while the electron density contribution for the LUMO was focused only on the biphenyl ring system (Fig. 3c). This calculated electronic structure is consistent with the emission properties of amino acid 8l. Electron donation from the 4-dimethylamino group through to the biphenyl ring system results in a charge-separated excited state that on de-excitation produces emission at longer wavelengths. In summary, the combination of the photophysical and DFT studies of 8l represents a comprehensive analysis of the properties of this amino acid and confirms its potential as a fluorescent probe for biological applications.

**Fig. 4:**
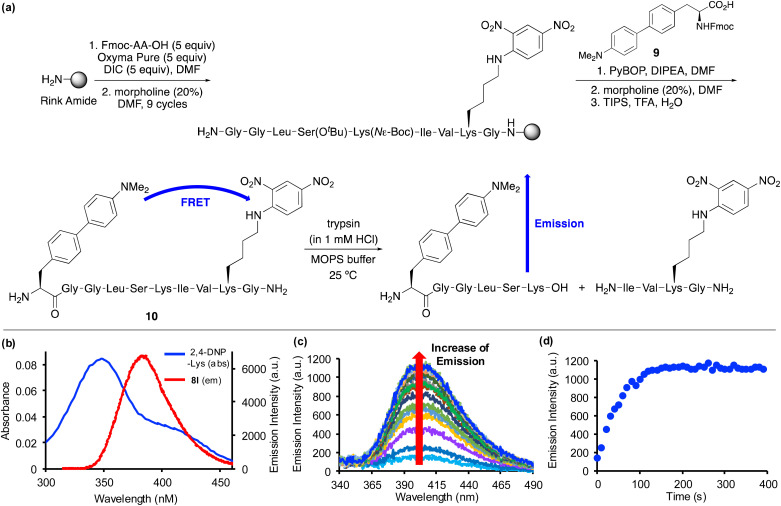
(a) SPPS synthesis of peptide 10, followed by trypsin-mediated digestion. (b) Spectroscopic overlap of absorption band of 2,4-DNP-lysine (blue line) and emission band of amino acid 8l (red line). (c) Emission spectra showing increasing fluorescence over time during trypsin cleavage of decapeptide 10. (d) Plot of emission intensity *versus* time during trypsin hydrolysis of decapeptide 10. The enzyme hydrolysis of peptide 10 used trypsin (0.01 μM), a range of peptide concentrations (0.50–2.50 μM) in 3-morpholinopropoanesulfonic acid (MOPS) buffer (20 mM) at pH 7.0 (*n* = 3).

Based on the positive photophysical properties of 8l and in particular the high quantum yield and strong brightness, we were interested in investigating its application as a FRET donor to report on protease activity (Fig. 4). FRET probes contain a fluorophore donor, which when excited, non-radiatively transfer the energy through long range dipole–dipole interactions to another fluorophore (acceptor) in the ground state.^27^ The emission of intact FRET-based peptides is quenched, but on enzymatic cleavage, fluorescence is turned on. These types of peptide probes are widely used for monitoring protease activity, which are important biomarkers for a range of diseases.^28,29^ Based on the emission wavelength of amino acid 8l, a scan of potential acceptors revealed significant overlap with the absorption band of 2,4-dinitrophenyl-lysine (Fig. 4b). Thus, in a proof-of-concept experiment, a decapeptide containing both amino acid 8l and 2,4-DNP-lysine was designed to monitor the activity of a protease. For this experiment, the serine protease, trypsin, one of the most widely used digesting enzymes for mass spectrometry-based proteomics and known to cleave substrates after lysine and arginine residues was chosen.^30^ The decapeptide sequence 10 was based on internally quenched fluorescent peptidic substrates reported by Poreba and co-workers who used a coumarin donor and 2,4-DNP-lysine acceptor pair to investigate the substrate specificity of various proteases.^29*b*^ Decapeptide 10 was prepared using SPPS methods (Fig. 4a). On coupling of Fmoc-glycine with the polymer support using *N*,*N*′-diisopropylcarbodiimide (DIC)/OxymaPure activation, subsequent rounds of morpholine-mediated *N*-deprotection and coupling with successive amino acids, including Fmoc-2,4-DNP-lysine gave the corresponding nonapeptide. Fmoc-protected amino acid 8l, compound 9 was then manually coupled with the polymer-supported nonapeptide.^31^ Following a final Fmoc-deprotection step, a TFA cleavage cocktail was used to remove the sidechain protecting groups and release decapeptide 10 from the polymer support. Purification by reverse-phase HPLC allowed isolation of decapeptide 10 in >99% purity. Characterisation of 10 by high resolution electrospray ionisation mass spectrometry verified the preparation of decapeptide 10 and the compatibility of amino acid 8l with SPPS methods. Excitation of decapeptide 10 at 300 nm resulted in near complete suppression of emission with only 10% activity retained, thus, confirming effective energy transfer between the 4-dimethylaminobiphenyl donor and the 2,4-DNP acceptor. The Förster distance (*R*_0_), which is the distance when energy transfer between a donor and acceptor is 50% efficient was calculated for the FRET pair (see ESI†). The Förster distance was found to be 36.45 Å, which is comparable to other commonly used FRET pairs.^1^^,^^29*b*^ On reaction with trypsin, decapeptide 10 was confirmed as a substrate with the restoration of fluorescence emission (Fig. 4c). The increasing emission intensity was monitored as a function of time (Fig. 4d) and this data was used to measure the protease activity. Using the observed pseudo-first order kinetics, *K*_M_ and *k*_cat_ values were calculated as 0.33 ± 0.073 μM and 0.73 ± 0.057 s^−1^ (*n* = 3), respectively. Overall, amino acid 8l was found to be compatible with SPPS, resulting in the straightforward preparation of an internally quenched decapeptide. Using the fluorescent properties of 8l as part of this peptidic substrate allowed measurement of the enzyme kinetics of trypsin-mediated hydrolytic cleavage.

## Conclusions

In summary, one-pot nonaflate activation and Suzuki–Miyaura cross-coupling of a tyrosine derivative has led to the expedient synthesis of biaryl α-amino acids, allowing rapid analysis of their photophysical properties and the discovery of several compounds with interesting fluorescent properties. From this library, amino acid 8l bearing a 4-dimethylaminobiphenyl side chain displayed the most red-shifted absorption and emission properties and with a quantum yield of 0.73, exhibited the highest brightness. A solvatochromic study and DFT calculations confirmed the charge-transfer nature of this fluorophore and further studies demonstrated its potential to act as a pH sensor with a major hypsochromic shift of the main emission band on acidification of the dimethylamino group. Due to the enhanced properties of 8l compared to other fluorescent polyaromatic amino acids previously used for measuring enzymatic kinetics, the final aim of this study demonstrated application of this novel fluorophore. An internally quenched fluorescent decapeptide containing amino acid 8l as a donor and 2,4-DNP-lysine as an acceptor was used to monitor and evaluate the kinetics of enzymatic hydrolysis by the serine protease, trypsin. Overall, this work demonstrates a fast and straightforward approach to access functionalised phenylalanine analogues with enhanced photophysical properties that in combination with SPPS techniques, can be incorporated into peptide substrates for biological applications. Current work is underway to further exploit the compatibility of these amino acids with SPPS and the preparation of fluorescent peptide substrates for new applications in biological chemistry.

## Data availability

All experimental and characterisation data, as well as photophysical and NMR spectra are available in the ESI.†

## Author contributions

A. R. T., and A. S. conceived the project. R. M. optimised the one-pot activation and cross-coupling reaction. O. M., R. M. and S. S. performed the synthesis of amino acids 8a–8m and conducted photophysical analysis. O. M. conducted full photophysical analysis of 8l, DFT calculations, synthesis of peptide 10, trypsin digestion assay and enzyme kinetic analysis. A. S. wrote the manuscript with comments from all authors.

## Conflicts of interest

There are no conflicts to declare.

## Supplementary Material

SC-016-D5SC00020C-s001
