# Supplementary material for: Expedient discovery of fluorogenic amino acid-based probes via one-pot palladium-catalysed arylation of tyrosine
Source: Chem Sci. 2025 Jan 23;16(8):3490–7. doi: 10.1039/d5sc00020c (PMC11775655; doi:10.1039/d5sc00020c)

Supplementary Information (SI) for Chemical Science.
This journal is © The Royal Society of Chemistry 2025

Supporting Information for:

Expedient discovery of fluorogenic amino acid-based probes via one-pot palladium-catalysed arylation of tyrosine

*Olivia Marshall, Rochelle McGrory, Sineenard Songsri, Andrew R. Thomson and Andrew Sutherland**

School of Chemistry, The Joseph Black Building, University of Glasgow,

Glasgow G12 8QQ, United Kingdom.

Table of Contents

1. General Experimental S2
2. Experimental Procedures and Spectroscopic Data for all Compounds S2–S17
3. Photophysical Data for α-Amino Acids **8a**–**m** S18–S30
4. Computational Data S31–S32
5. Peptide Synthesis S33–S34
6. Determination of Förster Distance for Amino acid **8l**/Lysine(dnp) Pair and Kinetic Parameters for Trypsin Digestion S35–S36
7. References S37
8. ^{1}H and $^{13}C\{^{1}H\}$ NMR Spectra for all Compounds S38–S90

1. General Experimental

All reagents and starting materials were obtained from commercial sources and used as received. Dry solvents were purified using a PureSolv 500 MD solvent purification system. All reactions were performed under an atmosphere of argon unless otherwise mentioned. Brine refers to a saturated solution of sodium chloride. Flash column chromatography was carried out using Fisher matrix silica 60. Macherey-Nagel aluminium-backed plates pre-coated with silica gel 60 (UV_{254}) were used for thin layer chromatography and visualised by staining with $KMnO_4$, vanillin or ninhydrin. ^{1}H NMR and ^{13}C NMR spectra were recorded on a Bruker DPX 400 or 500 spectrometer with chemical shift values in ppm relative to TMS (δ_H 0.00 and δ_C 0.0), or residual $CDCl_3$ (δ_H 7.26 and δ_C 77.2), DMSO-d_6 (δ_H 2.50 and δ_C 39.5) or CD_3OD (δ_H 3.31 and δ_C 49.0) as standard. ^{1}H and ^{13}C assignments are based on two-dimensional COSY and DEPT experiments, respectively. Mass spectra were obtained using a JEOL JMS-700 spectrometer for EI and CI or Bruker Microtof-q for ESI. Infrared spectra were obtained neat using a Shimadzu IR Prestige-21 spectrometer. Melting points were determined on a Reichert platform melting point apparatus. Optical rotations were determined as solutions irradiating with the sodium D line (λ= 589 nm) using an Autopol V polarimeter. $[\alpha]_D$ values are given in units 10^{-1} deg cm^2 g^{-1}.

2. Experimental Procedures and Spectroscopic Data for all Compounds

General Procedure 1: One-pot Nonaflate Formation and Suzuki-Miyaura Cross-Coupling Reaction. In a sealed tube, a solution of methyl (2*S*)-2-(benzyloxycarbonylamino)-3-(4-hydroxyphenyl)propanoate (1 equiv.) and potassium phosphate (3 equiv.) in acetonitrile (3 mL per mmol) was degassed under argon for 0.2 h. To this was added perfluoro-1-butanesulfonyl fluoride (1.5 equiv.). The reaction mixture was heated to 60 °C and stirred for 2 h. To this was added boronic acid (1.5 equiv.), XPhos Pd G2 (1 mol%) and water (2 mL per mmol). The reaction mixture was stirred at 60, 80 or 90 °C for 2 h. Additional XPhos Pd G2 (1 mol%) was added and the reaction mixture was stirred at 60, 80 or 90 °C for 2 h. A final portion of XPhos Pd G2 (1 mol%) was added and the reaction mixture was stirred at 60, 80 or 90 °C for 18 h. After cooling to room temperature, the reaction mixture was diluted in ethyl acetate (30 mL) and washed with water (3 × 30 mL). The organic layer was dried ($MgSO_4$), filtered and concentrated *in vacuo*. Purification by flash column chromatography gave the arylated products.

General Procedure 2: Ester Hydrolysis and Acid Mediated Removal of the Cbz-Protecting Group to Access Amino Acids 8a–m. To a stirred solution of the protected amino acid (1 equiv.) in methanol (3 mL), dioxane (1.75 mL) and water (1.75 mL) was added caesium carbonate (1.3 equiv.). The reaction mixture was heated to 60 °C and stirred for 18 h. The reaction mixture was cooled to room temperature

and concentrated *in vacuo*. The reaction mixture was diluted in water (5 mL), acidified to pH 1 using 1 M aqueous hydrochloric acid and extracted with ethyl acetate (3 × 20 mL). The organic layers were combined, dried ($MgSO_4$), filtered and concentrated *in vacuo* to give the desired carboxylic acid product. Without further purification, the carboxylic acid product was subsequently dissolved in 6 M hydrochloric acid (3 mL) and dioxane (3 mL) and heated under reflux for 4 h. The reaction mixture was cooled to room temperature and concentrated *in vacuo* to give the final amino acids. The amino acids were purified by recrystallisation from the specified solvent system.

Methyl (2*S*)-2-(benzyloxycarbonylamino)-3-(biphen-4'-yl)propanoate (7a)[1]

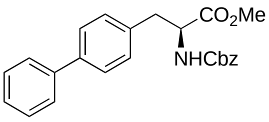

Methyl (2*S*)-2-(benzyloxycarbonylamino)-3-(biphen-4'-yl)propanoate (**7a**) was synthesised as described in general procedure 1 using methyl (2*S*)-2-(benzyloxycarbonylamino)-3-(4-hydroxyphenyl)propanoate (**6**) (0.15 g, 0.46 mmol), perfluoro-1-butanesulfonyl fluoride (0.12 mL, 0.68 mmol), phenylboronic acid (0.083 g, 0.68 mmol), XPhos Pd G2 (0.012 g, 0.015 mmol, 3 mol%) and potassium phosphate (0.29 g, 1.4 mmol) in acetonitrile (1.5 mL) and water (1 mL) at 60 °C. Purification by flash column chromatography, eluting with 30% ethyl acetate in hexane gave methyl (2*S*)-2-(benzyloxycarbonylamino)-3-(biphen-4'-yl)propanoate (**7a**) (0.16 g, 91%) as a white solid. Mp 85–90 °C (lit.[1] 83–85 °C); $[\alpha]_D^{22}$ +35.3 (*c* 0.1, $CHCl_3$); δ_H (400 MHz, $CDCl_3$) 3.14 (1H, dd, *J* 13.9, 5.9 Hz, 3-*H*H), 3.20 (1H, dd, *J* 13.9, 5.9 Hz 3-H*H*), 3.75 (3H, s, OCH_3), 4.67–4.77 (1H, m, 2-H), 5.11 (1H, d, *J* 12.0 Hz, C*H*HPh), 5.15 (1H, d, *J* 12.0 Hz, CH*H*Ph), 5.29 (1H, d, *J* 8.3 Hz, 2-NH), 7.14–7.22 (2H, m, 2'-H and 6'-H), 7.28–7.62 (12H, m, Ph, 3'-H, 5'-H, 2''-H, 3''-H, 4''-H, 5''-H and 6''-H); δ_C (101 MHz, $CDCl_3$) 38.0 (CH_2), 52.5 (CH_3), 54.9 (CH), 67.1 (CH_2), 127.1 (2 × CH), 127.41 (CH), 127.43 (2 × CH), 128.2 (2 × CH), 128.3 (CH), 128.6 (2 × CH), 128.9 (2 × CH), 129.8 (2 × CH), 134.9 (C), 136.4 (C), 140.1 (C), 140.8 (C), 155.8 (C), 172.1 (C); *m/z* (ESI) 412 (MNa^+. 100%).

Methyl (2*S*)-2-(benzyloxycarbonylamino)-3-(4"-phenylbiphen-4'-yl)propanoate (7b)

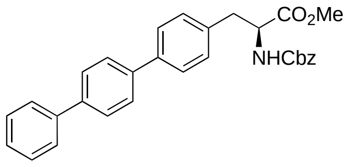

Methyl (2*S*)-2-(benzyloxycarbonylamino)-3-(4''-phenylbiphen-4'-yl)propanoate (**7b**) was synthesised as described in general procedure 1 using methyl (2*S*)-2-(benzyloxycarbonylamino)-3-(4-hydroxyphenyl)propanoate (**6**) (0.155 g, 0.471 mmol), perfluoro-1-butanesulfonyl fluoride (0.120 mL, 0.680 mmol), 4-biphenylboronic acid (0.136 g, 0.687 mmol), XPhos Pd G2 (0.0120 g, 0.0150 mmol, 3

mol%) and potassium phosphate (0.303 g, 1.43 mmol) in acetonitrile (1.5 mL) and water (1 mL) at 60 °C. After cooling to room temperature, the reaction mixture was diluted in chloroform (100 mL) and washed with water (3 × 50 mL). The organic layer was dried ($MgSO_4$), filtered and concentrated *in vacuo*. Purification by flash column chromatography, eluting with 80–100% dichloromethane in hexane gave methyl (2*S*)-2-(benzyloxycarbonylamino)-3-(4''-phenylbiphen-4'-yl)propanoate (**7b**) (0.169 g, 77%) as a white solid. Mp 184–185 °C; ν_{max}/cm^{-1} (neat) 3709 (NH), 3357, 2980 (CH), 2866, 2362, 1724 (C=O), 1052; $[\alpha]_D^{23}$ +47.8 (*c* 0.1, $CHCl_3$); δ_H (400 MHz, $CDCl_3$) 3.14 (1H, dd, *J* 14.0, 6.0 Hz, 3-*H*H), 3.20 (1H, dd, *J* 14.0, 5.6 Hz, 3-H*H*), 3.76 (3H, s, OCH_3), 4.66–4.76 (1H, m, 2-H), 5.10 (1H, d, *J* 8.0 Hz, C*H*HPh), 5.13 (1H, d, *J* 8.0 Hz, CH*H*Ph), 5.27 (1H, d, *J* 8.0 Hz, 2-NH), 7.19 (2H, d, *J* 8.2 Hz, 2'-H and 6'-H), 7.28–7.41 (6H, m, 6 × ArH), 7.43–7.51 (2H, m, 2 × ArH), 7.56 (2H, d, *J* 8.2 Hz, 3'-H and 5'-H), 7.61–7.75 (6H, m, 6 × ArH); δ_C (101 MHz, $CDCl_3$) 38.0 (CH_2), 52.5 (CH_3), 54.9 (CH), 67.2 (CH_2), 127.2 (2 × CH), 127.3 (2 × CH), 127.5 (4 × CH), 127.6 (2 × CH), 128.3 (CH), 128.4 (CH), 128.7 (2 × CH), 129.0 (2 × CH), 129.9 (2 × CH), 135.0 (C), 136.4 (C), 139.6 (C), 139.7 (C), 140.3 (C), 140.8 (C), 155.8 (C), 172.1 (C); *m/z* (ESI) 488.1840 (MNa^+. $C_{30}H_{27}NNaO_4$ requires 488.1832).

Methyl (2*S*)-2-(benzyloxycarbonylamino)-3-[(1''-naphthyl)phen-4'-yl]propanoate (7c)

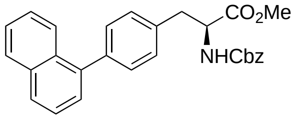

Methyl (2*S*)-2-(benzyloxycarbonylamino)-3-[(1''-naphthyl)phen-4'-yl]propanoate (**7c**) was synthesised as described in general procedure 1 using methyl (2*S*)-2-(benzyloxycarbonylamino)-3-(4-hydroxyphenyl)propanoate (**6**) (0.15 g, 0.46 mmol), perfluoro-1-butanesulfonyl fluoride (0.12 mL, 0.68 mmol), 1-naphthaleneboronic acid (0.12 g, 0.68 mmol), XPhos Pd G2 (0.012 g, 0.015 mmol, 3 mol%) and potassium phosphate (0.29 g, 1.4 mmol) in acetonitrile (1.5 mL) and water (1 mL) at 80 °C. Purification by flash column chromatography, eluting with 25% ethyl acetate in hexane gave methyl (2*S*)-2-(benzyloxycarbonylamino)-3-[(1''-naphthyl)phen-4'-yl]propanoate (**7c**) (0.17 g, 84%) as a colourless oil. ν_{max}/cm^{-1} (neat) 3320 (NH), 3050, 2954 (CH), 1746 (C=O), 1696 (C=O), 1506, 1249, 1211, 1181, 1057, 1014, 741; $[\alpha]_D^{25}$ +61.4 (*c* 0.1, $CHCl_3$); δ_H (400 MHz, $CDCl_3$) 3.19 (1H, dd, *J* 13.9, 6.0 Hz, 3-*H*H), 3.25 (1H, dd, *J* 13.9, 5.7 Hz 3-H*H*), 3.78 (3H, s, OCH_3), 4.71–4.81 (1H, m, 2-H), 5.12 (1H, d, *J* 12.0 Hz, C*H*HPh), 5.16 (1H, d, *J* 12.0 Hz, CH*H*Ph), 5.33 (1H, d, *J* 8.3 Hz, 2-NH), 7.22 (2H, d, *J* 7.8 Hz, 2'-H and 6'-H), 7.28–7.57 (11H, m, Ph, 3'-H, 5'-H, 2''-H, 3''-H, 6''-H and 7''-H), 7.83–7.96 (3H, m, 4''-H, 5''-H and 8''-H); δ_C (101 MHz, $CDCl_3$) 38.1 (CH_2), 52.6 (CH_3), 55.0 (CH), 67.2 (CH_2), 125.5 (CH), 125.9 (CH), 126.1 (CH), 126.2 (CH), 127.1 (2 × CH), 127.8 (CH), 128.3 (CH), 128.38 (2 × CH), 128.42 (CH), 128.7 (2 × CH), 129.4 (2 × CH), 130.4 (CH), 131.7 (C), 133.9 (C), 134.8

(C), 136.4 (C), 139.8 (C), 139.9 (C), 155.8 (C), 172.2 (C); *m/z* (ESI) 462.1679 (MNa^+. $C_{28}H_{25}NNaO_4$ requires 462.1676).

Methyl (2*S*)-2-(benzyloxycarbonylamino)-3-[(2''-naphthyl)phen-4'-yl] propanoate (7d)

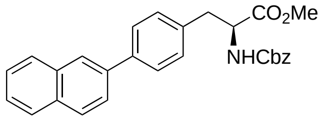

Methyl (2*S*)-2-(benzyloxycarbonylamino)-3-[(2''-naphthyl)phen-4'-yl]propanoate (**7d**) was synthesised as described in general procedure 1 using methyl (2*S*)-2-(benzyloxycarbonylamino)-3-(4-hydroxyphenyl)propanoate (**6**) (0.155 g, 0.472 mmol), perfluoro-1-butanesulfonyl fluoride (0.120 mL, 0.687 mmol), 2-naphthaleneboronic acid (0.0825 g, 0.480 mmol), XPhos Pd G2 (0.0120 g, 0.0150 mmol, 3 mol%) and potassium phosphate (0.304 g, 1.43 mmol) in acetonitrile (1.5 mL) and water (1 mL) at 90 °C. Purification by flash column chromatography, eluting with 5–10% ethyl acetate in hexane gave methyl (2*S*)-2-(benzyloxycarbonylamino)-3-[(2''-naphthyl)phen-4'-yl]propanoate (**7d**) (0.170 g, 81%) as a colourless oil. ν_{max}/cm^{-1} (neat) 3346 (NH), 3320, 3059, 2978 (CH), 1776 (C=O), 1651 (C=O), 1538, 1318, 1249, 1050, 1022; $[\alpha]_D^{25}$ +62.8 (*c* 0.1, $CHCl_3$); δ_H (400 MHz, $CDCl_3$) 3.17 (1H, dd, *J* 13.9, 6.1 Hz, 3-*H*H), 3.24 (1H, dd, *J* 13.9, 5.7 Hz 3-H*H*), 3.78 (3H, s, OCH_3), 4.72–4.79 (1H, m, 2-H), 5.12 (1H, d, *J* 12.0 Hz, C*H*HPh), 5.17 (1H, d, *J* 12.0 Hz, CH*H*Ph), 5.33 (1H, d, *J* 8.3 Hz, 2-NH), 7.23 (2H, d, *J* 7.8 Hz, 2'-H and 6'-H), 7.29–7.38 (5H, m, Ph), 7.47–7.55 (2H, m, 3'-H and 5'-H), 7.64–7.67 (2H, m, 6''-H and 7''-H), 7.73 (1H, dd, *J* 8.5, 1.8 Hz, 3''-H), 7.87–7.94 (3H, m, 4''-H, 5''-H and 8'-H), 8.03 (1H, d, *J* 1.8 Hz, 1''-H); δ_C (101 MHz, $CDCl_3$) 38.0 (CH_2), 52.5 (CH_3), 54.9 (CH), 67.1 (CH_2), 125.5 (CH), 125.8 (CH), 126.1 (CH), 126.4 (CH), 127.7 (2 × CH), 127.8 (CH), 128.2 (2 × CH), 128.29 (CH), 128.32 (CH), 128.55 (CH), 128.65 (2 × CH), 129.9 (2 × CH), 132.7 (C), 133.8 (C), 135.0 (C), 136.4 (C), 138.1 (C), 140.0 (C), 155.8 (C), 172.1 (C); *m/z* (ESI) 462.1688 (MNa^+. $C_{28}H_{25}NNaO_4$ requires 462.1676).

Methyl (2*S*)-2-(benzyloxycarbonylamino)-3-(2''-fluorobiphen-4'-yl)propanoate (7e)

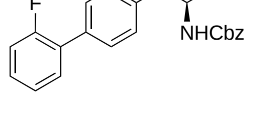

Methyl (2*S*)-2-(benzyloxycarbonylamino)-3-(2''-fluorobiphen-4'-yl)propanoate (**7e**) was synthesised as described in general procedure 1 using methyl (2*S*)-2-(benzyloxycarbonylamino)-3-(4-hydroxyphenyl)propanoate (**6**) (0.15 g, 0.46 mmol), perfluoro-1-butanesulfonyl fluoride (0.12 mL, 0.68 mmol), 2-fluorophenylboronic acid (0.096 g, 0.68 mmol), XPhos Pd G2 (0.012 g, 0.015 mmol, 3 mol%) and potassium phosphate (0.29 g, 1.4 mmol) in acetonitrile (1.5 mL) and water (1 mL) at 80 °C. Purification by flash column chromatography, eluting with 25% ethyl acetate in hexane gave methyl

(2*S*)-2-(benzyloxycarbonylamino)-3-(2''-fluorobiphen-4'-yl)propanoate (**7e**) (0.17 g, 94%) as a colourless oil. ν_{max}/cm^{-1} (neat) 3338 (NH), 2958 (CH), 2852, 1704 (C=O), 1494, 1198, 1050, 915, 728; $[\alpha]_D^{21}$ +70.3 (*c* 0.1, $CHCl_3$); δ_H (400 MHz, $CDCl_3$) 3.14 (1H, dd, *J* 15.9, 8.0 Hz, 3-*H*H), 3.20 (1H, dd, *J* 15.9, 8.0 Hz, 3-H*H*), 3.75 (3H, s, OCH_3), 4.67–4.76 (1H, m, 2-H), 5.10 (1H, d, *J* 12.0 Hz, C*H*HPh), 5.14 (1H, d, *J* 12.0 Hz, CH*H*Ph), 5.29 (1H, d, *J* 8.1 Hz, 2-NH), 7.10–7.24 (4H, m, 2'-H, 6'-H, 3''-H and 6''-H), 7.27–7.39 (6H, m, Ph and 4''-H), 7.42 (1H, td, *J* 7.8, 1.8 Hz, 5''-H), 7.47 (2H, dd, *J* 8.2, 1.7 Hz, 3'-H and 5'-H); δ_C (101 MHz, $CDCl_3$) 38.0 (CH_2), 52.5 (CH_3), 54.9 (CH), 67.2 (CH_2), 116.2 (d, $^2J_{CF}$ 22.8 Hz, CH), 124.5 (d, $^3J_{CF}$ 3.7 Hz, CH), 128.3 (2 × CH), 128.3 (CH), 128.7 (2 × CH), 128.7 (d, $^2J_{CF}$ 13.2 Hz, C), 129.1 (d, $^3J_{CF}$ 8.2 Hz, CH), 129.4 (d, $^4J_{CF}$ 3.7 Hz, 2 × CH), 129.5 (2 × CH), 130.8 (d, $^4J_{CF}$ 3.5 Hz, CH), 134.8 (C), 135.3 (C), 136.4 (C), 155.8 (C), 159.9 (d, $^1J_{CF}$ 247.8 Hz, C), 172.1 (C); *m/z* (ESI) 430.1428 (MNa^+. $C_{24}H_{22}FNNaO_4$ requires 430.1425).

Methyl (2*S*)-2-(benzyloxycarbonylamino)-3-(4''-fluorobiphen-4'-yl)propanoate (7f)

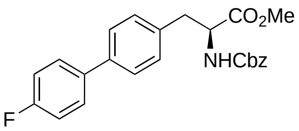

Methyl (2*S*)-2-(benzyloxycarbonylamino)-3-(4''-fluorobiphen-4'-yl)propanoate (**7f**) was synthesised as described in general procedure 1 using methyl (2*S*)-2-(benzyloxycarbonylamino)-3-(4-hydroxyphenyl)propanoate (**6**) (0.155 g, 0.471 mmol), perfluoro-1-butanesulfonyl fluoride (0.120 mL, 0.680 mmol), 4-fluorophenylboronic acid (0.110 g, 0.729 mmol), XPhos Pd G2 (0.0120 g, 0.0150 mmol, 3 mol%) and potassium phosphate (0.307 g, 1.45 mmol) in acetonitrile (1.5 mL) and water (1 mL) at 60 °C. Purification by flash column chromatography, eluting with 20% ethyl acetate in hexane gave methyl (2*S*)-2-(benzyloxycarbonylamino)-3-(4''-fluorobiphen-4'-yl)propanoate (**7f**) (0.127 g, 69%) as a pale yellow solid. Mp 108–109 °C; ν_{max}/cm^{-1} (neat); 3343 (NH), 2366, 1741 (C=O), 1705 (C=O), 1519, 1491, 1214; $[\alpha]_D^{25}$ +54.1 (*c* 0.1, $CHCl_3$); δ_H (400 MHz, $CDCl_3$) 3.12 (1H, dd, *J* 13.8, 6.0 Hz, 3-*H*H), 3.19 (1H, dd, *J* 13.8, 5.6 Hz, 3-H*H*), 3.75 (3H, s, OCH_3), 4.66–4.75 (1H, m, 2-H), 5.09 (1H, d, *J* 12.4 Hz, C*H*HPh), 5.13 (1H, d, *J* 12.4 Hz, CH*H*Ph), 5.26 (1H, d, *J* 8.3 Hz, 2-NH), 7.01–7.18 (4H, m, 2'-H, 6'-H, 3''-H and 5''-H), 7.29–7.39 (5H, m, 7.45, Ph), 7.44 (2H, d, *J* 8.0 Hz, 3'-H and 5'-H), 7.48–7.56 (2H, m, 2''-H and 6''-H); δ_C (101 MHz, $CDCl_3$) 37.9 (CH_2), 52.6 (CH_3), 54.9 (CH), 67.2 (CH_2), 115.8 (2 × CH, d, $^2J_{CF}$ 21.4 Hz), 127.3 (2 × CH), 128.3 (2 × CH), 128.4 (CH), 128.7 (2 × CH, d, $^3J_{CF}$ 8.0 Hz), 128.7 (2 × CH) 129.9 (2 × CH), 134.9 (C), 136.3 (C), 136.9 (C), 139.2 (C), 155.8 (C), 162.6 (C, d, $^1J_{CF}$ 246.3 Hz), 172.1 (C); *m/z* (ESI) 430.1432 (MNa^+. $C_{24}H_{22}FNNaO_4$ requires 430.1425).

Methyl (2*S*)-2-(benzyloxycarbonylamino)-3-[4''-(chlorobiphen-4'-yl] propanoate (7g)

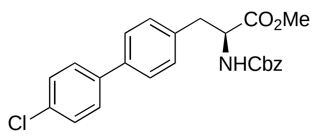

Methyl (2*S*)-2-(benzyloxycarbonylamino)-3-[4''-(chlorobiphen-4'-yl]propanoate (**7g**) was synthesised as described in general procedure 1 using methyl (2*S*)-2-(benzyloxycarbonylamino)-3-(4-hydroxyphenyl)propanoate (**6**) (0.15 g, 0.46 mmol), perfluoro-1-butanesulfonyl fluoride (0.12 mL, 0.68 mmol), 4-chlorophenylboronic acid (0.13 g, 0.68 mmol), XPhos Pd G2 (0.012 g, 0.015 mmol, 3 mol%) and potassium phosphate (0.29 g, 1.40 mmol) in acetonitrile (1.5 mL) and water (1 mL) at 80 °C. Purification by flash column chromatography, eluting with 30% ethyl acetate in gave methyl (2*S*)-2-(benzyloxycarbonylamino)-3-[4''-(chlorobiphen-4'-yl]propanoate (**7g**) (0.12 g, 61%) as a white solid. Mp 90–92 °C; ν_{max}/cm^{-1} (neat) 3341 (NH), 3031, 2955 (CH), 1717 (C=O), 1515, 1487, 1347, 1258, 1212; $[\alpha]_D^{21}$ +53.0 (*c* 0.1, $CHCl_3$); δ_H (400 MHz, $CDCl_3$) 3.13 (1H, dd, *J* 13.9, 5.7 Hz, 3-*H*H), 3.21 (1H, dd, *J* 13.9, 6.1 Hz 3-H*H*), 3.75 (3H, s, OCH_3), 4.68–4.75 (1H, m, 2-H), 5.10 (1H, d, *J* 12.0 Hz, C*H*HPh), 5.14 (1H, d, *J* 12.0 Hz, CH*H*Ph), 5.30 (1H, d, *J* 8.3 Hz, 2-NH), 7.16–7.20 (2H, m, 2'-H and 6'-H), 7.30–7.52 (11H, m, Ph, 3'-H, 5'-H, 2''-H, 3''-H, 5''-H and 6''-H); δ_C (101 MHz, $CDCl_3$) 37.9 (CH_2), 52.5 (CH_3), 54.9 (CH), 67.1 (CH_2), 127.2 (2 × CH), 128.2 (2 × CH), 128.3 (3 × CH), 128.6 (2 × CH), 129.0 (2 × CH), 129.9 (2 × CH), 133.5 (C), 135.3 (C), 136.3 (C), 138.9 (C), 139.2 (C), 155.7 (C), 172.0 (C); *m/z* (ESI) 446.1131 (MNa^+. $C_{24}H_{22}{}^{35}ClNNaO_4$ requires 446.1130).

Methyl (2*S*)-2-(benzyloxycarbonylamino)-3-[4''-(trifluoromethyl)biphen-4'-yl]propanoate (7h)

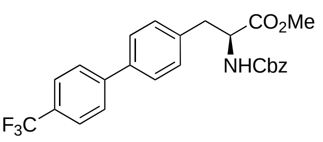

Methyl (2*S*)-2-(benzyloxycarbonylamino)-3-[4''-(trifluoromethyl)biphen-4'-yl]propanoate (**7h**) was synthesised as described in general procedure 1 using methyl (2*S*)-2-(benzyloxycarbonylamino)-3-(4-hydroxyphenyl)propanoate (**6**) (0.15 g, 0.46 mmol), perfluoro-1-butanesulfonyl fluoride (0.12 mL, 0.68 mmol), 4-(trifluoromethyl)phenylboronic acid (0.13 g, 0.68 mmol), XPhos Pd G2 (0.012 g, 0.015 mmol, 3 mol%) and potassium phosphate (0.29 g, 1.4 mmol) in acetonitrile (1.5 mL) and water (1 mL) at 80 °C. Purification by flash column chromatography, eluting with 25% ethyl acetate in gave methyl (2*S*)-2-(benzyloxycarbonylamino)-3-[(4''-trifluoromethyl)biphen-4'-yl]propanoate (**7h**) (0.16 g, 75%) as a white solid. Mp 125–130 °C; ν_{max}/cm^{-1} (neat) 3316 (NH), 3061, 2878 (CH), 2388, 1776 (C=O), 1696 (C=O), 1537, 1392, 1335, 1267; $[\alpha]_D^{15}$ +28.2 (*c* 0.1, $CHCl_3$); δ_H (400 MHz, $CDCl_3$) 3.14 (1H, dd, *J* 13.9, 6.0 Hz, 3-*H*H), 3.21 (1H, dd, *J* 13.9, 5.7 Hz 3-H*H*), 3.76 (3H, s, OCH_3), 4.68–4.76 (1H, m, 2-H), 5.08 (1H, d, *J* 12.0 Hz, C*H*HPh), 5.14 (1H, d, *J* 12.0 Hz, CH*H*Ph), 5.25 (1H, d, *J* 8.2 Hz, 2-NH), 7.18–7.22 (2H, m, 2'-H and 6'-H), 7.28–7.39 (5H, m, Ph), 7.48–7.53 (2H, m, 3'-H and 5'-H), 7.63–

7.72 (4H, m, 2’’-H, 3’’-H, 5’’-H and 6’’-H); δ_C (101 MHz, $CDCl_3$) 38.0 (CH_2), 52.6 (CH_3), 54.9 (CH), 67.2 (CH_2), 124.9 (q, ${}^1J_{CF}$ 272.1 Hz, C), 125.9 (q, ${}^3J_{CF}$ 3.9 Hz, 2 × CH), 127.4 (2 × CH), 127.6 (2 × CH), 128.3 (CH), 128.4 (2 × CH), 128.7 (2 × CH), 129.5 (q, ${}^2J_{CF}$ 32.6 Hz, C), 130.1 (2 × CH), 136.0 (C), 136.3 (C), 138.7 (C), 144.3 (C), 155.8 (C), 172.0 (C); *m/z* (ESI) 480.1395 (MNa^+. $C_{25}H_{22}F_3NNaO_4$ requires 480.1393).

Methyl (2*S*)-2-(benzyloxycarbonylamino)-3-(4’’-acetylbiphen-4’-yl)propanoate (7i)

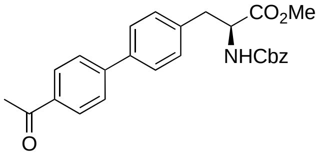

Methyl (2*S*)-2-(benzyloxycarbonylamino)-3-(4’’-acetylbiphen-4’-yl)propanoate (**7i**) was synthesised as described in general procedure 1 using methyl (2*S*)-2-(benzyloxycarbonylamino)-3-(4-hydroxyphenyl)propanoate (**6**) (0.15 g, 0.46 mmol), perfluoro-1-butanesulfonyl fluoride (0.12 mL, 0.68 mmol), 4-acetylphenylboronic acid (0.10 g, 0.68 mmol), XPhos Pd G2 (0.012 g, 0.015 mmol, 3 mol%) and potassium phosphate (0.29 g, 1.4 mmol) in acetonitrile (1.5 mL) and water (1 mL) at 60 °C. Purification by flash column chromatography, eluting with 20% ethyl acetate in hexane gave methyl (2*S*)-2-(benzyloxycarbonylamino)-3-(4’’-acetylbiphen-4’-yl)propanoate (**7i**) (0.16 g, 81%) as a white solid. Mp 125–128 °C; ν_{max}/cm^{-1} (neat) 3332 (NH), 2952 (CH), 2251, 1716 (C=O), 1676 (C=O), 1522, 1354, 1264, 1207, 733; $[\alpha]_D^{25}$ +55.5 (*c* 0.1, $CHCl_3$); δ_H (400 MHz, $CDCl_3$) 2.64 (3H, s, 4’’-$COCH_3$), 3.13 (1H, dd, *J* 13.9, 6.2 Hz, 3-*H*H), 3.22 (1H, dd, *J* 13.9, 5.7 Hz, 3-H*H*), 3.76 (3H, s, OCH_3), 4.68–4.75 (1H, m, 2-H), 5.09 (1H, d, *J* 12.0 Hz, C*H*HPh), 5.13 (1H, d, *J* 12.0 Hz, CH*H*Ph), 5.27 (1H, d, *J* 8.2 Hz, 2-NH), 7.20 (2H, d, *J* 8.4 Hz, 2’-H and 6’-H), 7.27–7.38 (5H, m, Ph), 7.54 (2H, d, *J* 8.4 Hz, 3’-H and 5’-H), 7.66 (2H, d, *J* 8.4 Hz, 2’’-H and 6’’-H), 8.03 (2H, d, *J* 8.4 Hz, 3’’-H and 5’’-H); δ_C (101 MHz, $CDCl_3$) 26.8 (CH_3), 38.0 (CH_2), 52.6 (CH_3), 54.9 (CH), 67.2 (CH_2), 127.2 (2 × CH), 127.6 (2 × CH), 128.2 (CH), 128.4 (2 × CH), 128.7 (2 × CH), 129.1 (2 × CH), 130.0 (2 × CH), 136.0 (C), 136.1 (C), 136.3 (C), 138.8 (C), 145.4 (C), 155.7 (C), 172.0 (C), 197.9 (C); *m/z* (ESI) 454.1631 (MNa^+. $C_{26}H_{25}NNaO_5$ requires 454.1625).

Methyl (2*S*)-2-(benzyloxycarbonylamino)-3-(2’’-methoxybiphen-4’-yl)propanoate (7j)

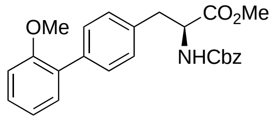

Methyl (2*S*)-2-(benzyloxycarbonylamino)-3-(2’’-methoxybiphen-4’-yl)propanoate (**7j**) was synthesised as described in general procedure 1 using methyl (2*S*)-2-(benzyloxycarbonylamino)-3-(4-hydroxyphenyl)propanoate (**6**) (0.155 g, 0.471 mmol), perfluoro-1-butanesulfonyl fluoride (0.120 mL,

0.680 mmol), 2-methoxyphenylboronic acid (0.139 g, 0.915 mmol), XPhos Pd G2 (0.012 g, 0.015 mmol, 3 mol%) and potassium phosphate (0.307 g, 1.45 mmol) in acetonitrile (1.5 mL) and water (1 mL) at 60 °C. Purification by flash column chromatography, eluting with 20% ethyl acetate in hexane gave methyl (2*S*)-2-(benzyloxycarbonylamino)-3-(2''-methoxybiphen-4'-yl)propanoate (**7j**) (0.137 g, 70%) as a clear oil. ν_{max}/cm^{-1} (neat) 3331 (NH), 2951 (CH), 2365, 1715 (C=O), 1510, 1487, 1240, 1214, 1058, 1023, 757; $[\alpha]_D^{25}$ +50.8 (*c* 0.1, $CHCl_3$); δ_H (400 MHz, $CDCl_3$) 3.13 (1H, dd, *J* 14.0, 6.4 Hz, 3-*H*H), 3.18 (1H, dd, *J* 14.0, 5.6 Hz, 3-H*H*), 3.75 (3H, s, OCH_3), 3.80 (4''-OCH_3), 4.65–4.73 (1H, m, 2-H), 5.06–5.16 (2H, m, CH_2Ph), 5.26 (1H, d, *J* 8.3 Hz, 2-NH), 6.98 (1H, br d, *J* 8.4 Hz, 3''-H), 7.02 (1H, td, *J* 7.5, 1.1 Hz, 5''-H), 7.11–7.16 (2H, m, 3'-H and 5'-H), 7.28–7.37 (7H, m, 2'-H, 6'-H and Ph), 7.43–7.48 (4''-H and 6''-H); δ_C (101 MHz, $CDCl_3$) 38.0 (CH_2), 52.5 (CH_3), 54.9 (CH), 55.6 (CH_3), 67.2 (CH_2), 111.4 (CH), 121.0 (CH), 128.2 (CH), 128.3 (CH), 128.7 (2 × CH), 128.8 (CH) 129.1 (2 × CH), 129.9 (2 × CH), 130.3 (C), 130.9 (2 × CH), 134.3 (C), 136.4 (C), 137.5 (C), 155.8 (C), 156.6 (C), 172.2 (C); *m/z* (ESI) 442.1629 (MNa^+. $C_{25}H_{25}NNaO_5$ requires 442.1625).

Methyl (2*S*)-2-(benzyloxycarbonylamino)-3-(4''-methoxybiphen-4'-yl)propanoate (7k)

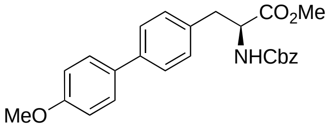

Methyl (2*S*)-2-(benzyloxycarbonylamino)-3-(4''-methoxybiphen-4'-yl)propanoate (**7k**) was synthesised as described in general procedure 1 using methyl (2*S*)-2-(benzyloxycarbonylamino)-3-(4-hydroxyphenyl)propanoate (**6**) (0.15 g, 0.46 mmol), perfluoro-1-butanesulfonyl fluoride (0.12 mL, 0.68 mmol), 4-methoxyphenylboronic acid (0.10 g, 0.68 mmol), XPhos Pd G2 (0.012 g, 0.015 mmol, 3 mol%) and potassium phosphate (0.29 g, 1.4 mmol) in acetonitrile (1.5 mL) and water (1 mL) at 60 °C. Purification by flash column chromatography, eluting with 20% ethyl acetate in hexane gave methyl (2*S*)-2-(benzyloxycarbonylamino)-3-(4''-methoxybiphen-4'-yl)propanoate (**7k**) (0.16 g, 81%) as a white solid. Mp 102–104 °C; ν_{max}/cm^{-1} (neat) 2924 (CH), 2855, 2253, 1717 (C=O), 1501, 1246, 907; $[\alpha]_D^{17}$ +27.3 (*c* 0.1, $CHCl_3$); δ_H (400 MHz, $CDCl_3$) 3.12 (1H, dd, *J* 13.9, 6.0 Hz, 3-*H*H), 3.18 (1H, dd, *J* 13.9, 5.6 Hz, 3-H*H*), 3.75 (3H, s, OCH_3), 3.85 (4''-OCH_3), 4.65–4.73 (1H, m, 2-H), 5.09 (1H, d, *J* 12.0 Hz, C*H*HPh), 5.13 (1H, d, *J* 12.0 Hz, CH*H*Ph), 5.24 (1H, d, *J* 8.3 Hz, 2-NH), 6.94–7.00 (2H, m, 3''-H and 5''-H), 7.11–7.16 (2H, m, 3'-H and 5'-H), 7.28–7.37 (5H, m, Ph), 7.43–7.53 (4H, m, 2'-H, 6'-H, 2''-H and 6''-H); δ_C (101 MHz, $CDCl_3$) 38.0 (CH_2), 52.5 (CH_3), 54.9 (CH), 55.5 (CH_3), 67.1 (CH_2), 114.4 (2 × CH), 127.0 (2 × CH), 128.2 (2 × CH), 128.25 (CH), 128.34 (2 × CH), 128.7 (2 × CH), 129.8 (2 × CH), 133.4 (C), 134.2 (C), 136.4 (C), 139.8 (C), 155.8 (C), 159.3 (C), 172.1 (C); *m/z* (ESI) 420.1805 (MH^+. $C_{25}H_{26}NO_5$ requires 420.1805).

Methyl (2*S*)-2-(benzyloxycarbonylamino)-3-(4''-dimethylaminobiphen-4'-yl)propanoate (7l)

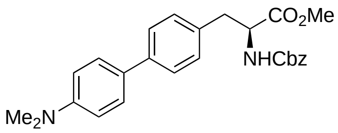

Methyl (2*S*)-2-(benzyloxycarbonylamino)-3-(4''-dimethylaminobiphen-4'-yl)propanoate (**7l**) was synthesised as described in general procedure 1 using methyl (2*S*)-2-(benzyloxycarbonylamino)-3-(4-hydroxyphenyl)propanoate (**6**) (0.452 g, 1.37 mmol), perfluoro-1-butanesulfonyl fluoride (0.370 mL, 2.05 mmol), 4-dimethylaminophenylboronic acid (0.451 g, 2.73 mmol), XPhos Pd G2 (0.0140 g, 0.0180 mmol, 3 mol%) and potassium phosphate (0.875 g, 4.12 mmol) in acetonitrile (4.5 mL) and water (3 mL) at 80 °C. Purification by flash column chromatography, eluting with 20% ethyl acetate in hexane followed by recrystallisation in chloroform/hexane gave methyl (2*S*)-2-(benzyloxycarbonylamino)-3-(4''-dimethylaminobiphen-4'-yl)propanoate (**7l**) (0.398 g, 67%) as a pale yellow solid. Mp 144–146 °C; ν_{max}/cm^{-1} (neat) 3343 (NH), 2952 (CH), 1713 (C=O), 1609 (C=C), 1505, 1347, 1207, 1060; $[\alpha]_D^{25}$ +60.4 (*c* 0.1, $CHCl_3$); δ_H (400 MHz, $CDCl_3$) 3.00 (6H, s, N(C*H*$_3$)$_2$), 3.11 (1H, dd, *J* 14.0, 6.0 Hz, 3-*H*H), 3.16 (1H, dd, *J* 14.0, 5.6 Hz, 3-H*H*), 3.74 (3H, s, OCH_3), 4.62–4.73 (1H, m, 2-H), 5.09 (1H, d, *J* 12.4 Hz, C*H*HPh), 5.13 (1H, d, *J* 12.4 Hz, CH*H*Ph), 5.24 (1H, d, *J* 8.3 Hz, NH), 6.80 (2H, d, *J* 8.8 Hz, 3''-H and 5''-H), 7.10 (2H, d, *J* 8.2 Hz, 3'-H and 5'-H), 7.26–7.40 (5H, m, Ph), 7.46 (2H, d, *J* 8.2 Hz, 2'-H and 6'-H), 7.48 (2H, d, *J* 8.8 Hz, 2''-H and 6''-H); δ_C (101 MHz, $CDCl_3$) 37.9 (CH_2), 40.7 (2 × CH_3), 52.5 (CH_3), 54.9 (CH), 67.1 (CH_2), 112.9 (2 × CH), 126.6 (2 × CH), 127.7 (2 × CH), 128.2 (2 × CH), 128.3 (CH), 128.7 (2 × CH), 128.8 (C), 129.7 (2 × CH), 133.3 (C), 136.4 (C), 140.2 (C), 150.1 (C), 155.8 (C), 172.2 (C); *m/z* (ESI) 433.2133 (MH^+. $C_{26}H_{29}N_2O_4$ requires 433.2122).

Methyl (2*S*)-2-(benzyloxycarbonylamino)-3-[4'-phenyl(thiophen-3''-yl)]propanoate (7m)

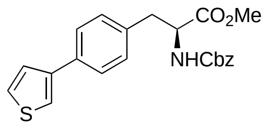

Methyl (2*S*)-2-(benzyloxycarbonylamino)-3-[4'-phenyl(thiophen-3''-yl)]propanoate (**7m**) was synthesised as described in general procedure 1 using methyl (2*S*)-2-(benzyloxycarbonylamino)-3-(4-hydroxyphenyl)propanoate (**6**) (0.156 g, 0.474 mmol), perfluoro-1-butanesulfonyl fluoride (0.120 mL, 0.680 mmol), 3-thienylboronic acid (0.0909 g, 0.710 mmol), XPhos Pd G2 (0.0120 g, 0.0150 mmol, 3 mol%) and potassium phosphate (0.300 g, 1.41 mmol) in acetonitrile (1.5 mL) and water (1 mL) at 80 °C. Purification by flash column chromatography, eluting with 20% ethyl acetate in hexane gave methyl (2*S*)-2-(benzyloxycarbonylamino)-3-[4'-phenyl(thiophen-3''-yl)]propanoate (**7m**) (0.127 g, 68%) as a white solid. Mp 126–128 °C; ν_{max}/cm^{-1} (neat) 3408 (NH), 2962 (CH), 1745 (C=O), 1713 (C=O), 1505, 1441, 1207; $[\alpha]_D^{25}$ +15.4 (*c* 0.1, $CHCl_3$); δ_H (400 MHz, $CDCl_3$) 3.11 (1H, dd, *J* 14.0, 6.0 Hz, 3-*H*H), 3.18 (1H, dd, *J* 14.0, 5.6 Hz, 3-H*H*), 3.74 (3H, s, OCH_3), 4.65–4.74 (1H, m, 2-H), 5.09 (1H, d, *J* 12.4

Hz, CHHPh), 5.14 (1H, d, J 12.4 Hz, CHHPh), 5.31 (1H, d, J 8.4 Hz, NH), 7.14 (2H, d, J 8.0 Hz, 3'-H and 5'-H), 7.29–7.44 (8H, m, 2''-H, 4''-H, 5''-H and Ph), 7.51 (2H, d J 8.0 Hz, 2'-H and 6'-H); δ_C (101 MHz, $CDCl_3$) 38.0 (CH_2), 52.4 (CH_3), 54.9 (CH), 67.1 (CH_2), 120.3 (CH), 126.29 (CH), 126.35 (CH), 126.7 (2 × CH), 128.2 (2 × CH), 128.3 (CH), 128.6 (2 × CH), 129.8 (2 × CH), 134.7 (C), 134.8 (C), 136.3 (C), 142.0 (C), 155.7 (C), 172.0 (C); *m/z* (ESI) 418.1087 (MNa^+. $C_{22}H_{21}NNaO_4S$ requires 418.1083).

(2*S*)-2-Amino-3-(biphen-4'-yl)propanoic acid hydrochloride (8a)[2]

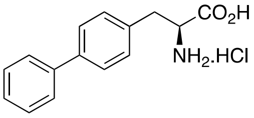

(2*S*)-2-Amino-3-(biphen-4'-yl)propanoic acid (**8a**) was synthesised according to general procedure 2 using methyl (2*S*)-2-(benzyloxycarbonylamino)-3-(biphen-4'-yl)propanoate (**7a**) (0.137 g, 0.352 mmol) and caesium carbonate (0.149 g, 0.457 mmol). Recrystallisation from methanol and diethyl ether gave (2*S*)-2-amino-3-(biphen-4'-yl)propanoic acid hydrochloride (**8a**) (0.0840 g, 86%) as a white solid. Mp 256–258 °C (lit.[2] 256–258 °C); $[\alpha]_D^{18}$ –80.7 (*c* 0.1, MeOH); δ_H (400 MHz, CD_3OD) 3.20 (1H, dd, J 14.5, 7.9 Hz, 3-HH), 3.38 (1H, dd, J 14.5, 5.4 Hz 3-HH), 4.29 (1H, dd, J 7.9, 5.4 Hz, 2-H), 7.33–7.49 (5H, m, 2'-H, 6'-H, 3''-H, 4''-H and 5''-H), 7.59–7.68 (4H, m, 3'-H, 5'-H, 2''-H and 6''-H); δ_C (101 MHz, CD_3OD) 36.9 (CH_2), 55.1 (CH), 127.9 (2 × CH), 128.5 (CH), 128.7 (2 × CH), 129.9 (2 × CH), 131.0 (2 × CH), 134.6 (C), 141.8 (C), 142.0 (C), 171.1 (C); *m/z* (ESI) 242 (MH^+. 100%).

(2*S*)-2-Amino-3-(4"-phenylbiphen-4'-yl)propanoic acid hydrochloride (8b)

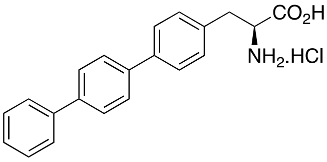

(2*S*)-2-Amino-3-(4"-phenylbiphen-4'-yl)propanoic acid hydrochloride (**8b**) was synthesised according to general procedure 2 using methyl (2*S*)-2-(benzyloxycarbonylamino)-3-(4"-phenylbiphen-4'-yl)propanoate (**7b**) (0.150 g, 0.322 mmol) and caesium carbonate (0.136 g, 0.385 mmol). Recrystallisation from methanol and diethyl ether gave (2*S*)-2-amino-3-(4"-phenylbiphen-4'-yl)propanoic acid hydrochloride (**8b**) (0.0650 g, 53%) as a white solid. Mp 275–280 °C (decomposed); ν_{max}/cm^{-1} (neat) 3418 (OH), 3027 (NH), 2930 (CH), 2356, 2337, 1680 (C=O), 1594 (C=C), 1483; $[\alpha]_D^{15}$ +12.0 (*c* 0.1, MeOH); δ_H (400 MHz, CD_3OD) 3.22 (1H, dd, J 14.4, 8.0 Hz, 3-HH), 3.38 (1H, dd, J 14.4, 5.6 Hz, 3-HH), 4.28–4.33 (1H, m, 2-H), 7.32–7.50 (5H, m, ArH), 7.61–7.73 (8H, m, ArH); δ_C (101 MHz, CD_3OD) 37.0 (CH_2), 55.1 (CH), 127.9 (2 × CH), 128.3 (3 × CH), 128.5 (2 × CH), 128.6 (2 ×

CH), 129.9 (2 × CH), 131.1 (2 × CH), 134.7 (C), 140.6 (C), 141.5 (C), 141.6 (C), 141.8 (C), 171.2 (C); *m/z* (ESI) 318.1491 (MH^+. $C_{21}H_{20}NO_2$ requires 318.1489).

(2*S*)-2-Amino-3-[(1''-naphthyl)phen-4'-yl]propanoic acid hydrochloride (8c)

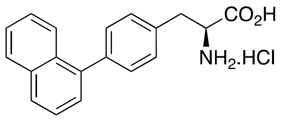

(2*S*)-2-Amino-3-[(1''-naphthyl)phen-4'-yl]propanoic acid hydrochloride (**8c**) was synthesised according to general procedure 2 using methyl (2*S*)-2-(benzyloxycarbonylamino)-3-[(1''-naphthyl)phen-4'-yl]propanoate (**7c**) (0.128 g, 0.291 mmol) and caesium carbonate (0.124 g, 0.379 mmol). Recrystallisation from methanol and diethyl ether gave (2*S*)-2-amino-3-[(1''-naphthyl)phen-4'-yl]propanoic acid hydrochloride (**8c**) (0.0534 g, 56%) as a white solid. Mp 230–236 °C; ν_{max}/cm^{-1} (neat) 2856 (CH), 1768 (C=O), 1692 (C=O), 1598, 1491, 1392, 1324, 1183, 1119, 764; $[\alpha]_D^{16}$ –10.0 (*c* 0.1, MeOH); δ_H (400 MHz, CD_3OD) 3.27 (1H, dd, *J* 14.5, 7.7 Hz, 3-*H*H), 3.43 (1H, dd, *J* 14.5, 5.3 Hz 3-H*H*), 4.32 (1H, dd, *J* 7.7, 5.3 Hz, 2-H), 7.34–7.56 (8H, m, 2'-H, 3'-H, 5'-H, 6'-H, 2''-H, 3''-H, 6''-H and 7''-H), 7.83–7.95 (3H, m, 4''-H, 5''-H and 8''-H); δ_C (101 MHz, CD_3OD) 37.2 (CH_2), 55.3 (CH), 126.4 (CH), 126.7 (CH), 126.9 (CH), 127.1 (CH), 127.9 (CH), 128.9 (CH), 129.4 (CH), 130.5 (2 × CH), 131.8 (2 × CH), 132.8 (C), 134.8 (C), 135.4 (C), 140.9 (C), 141.7 (C), 171.4 (C); *m/z* (ESI) 292.1342 (MH^+. $C_{19}H_{18}NO_2$ requires 292.1332).

(2*S*)-2-Amino-3-[(2''-naphthyl)phen-4'-yl]propanoic acid hydrochloride (8d)

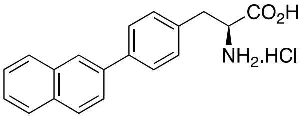

(2*S*)-2-Amino-3-[(2''-naphthyl)phen-4'-yl]propanoic acid hydrochloride (**8d**) was synthesised according to general procedure 2 using methyl (2*S*)-2-(benzyloxycarbonylamino)-3-[(2''-naphthyl)phen-4'-yl]propanoate (**8d**) (0.072 g, 0.16 mmol) and caesium carbonate (0.068 g, 0.21 mmol). Recrystallisation from methanol and diethyl ether gave (2*S*)-2-amino-3-[(2''-naphthyl)phen-4'-yl]propanoic acid hydrochloride (**8d**) (0.040 g, 76%) as a white solid. Mp 236–240 °C; ν_{max}/cm^{-1} (neat) 3331 (NH), 2856 (CH), 1768 (C=O), 1692 (C=O), 1598, 1491, 1392, 1324, 1183, 1119; $[\alpha]_D^{23}$ –11.0 (*c* 0.1, MeOH); δ_H (400 MHz, CD_3OD) 3.24 (1H, dd, *J* 14.1, 7.6 Hz, 3-*H*H), 3.39 (1H, dd, *J* 14.1, 5.6 Hz, 3-H*H*), 4.32 (1H, dd, *J* 7.6, 5.6 Hz, 2-H), 7.41–7.54 (4H, m, 2'-H, 3'-H, 6''-H and 7''-H), 7.75–7.81 (3H, m, 5'-H, 6'-H and 3''-H), 7.83–7.96 (3H, m, 4''-H, 5''-H and 8''-H), 8.09 (1H, br s, 1''-H); δ_C (101 MHz, CD_3OD) 37.0 (CH_2), 55.1 (CH), 126.2 (CH), 126.5 (CH), 127.1 (CH), 127.4 (CH), 128.6 (CH), 128.9 (2 × CH), 129.2 (CH), 129.6 (CH), 131.1 (2 × CH), 134.2 (C), 134.7 (C), 135.2 (C), 139.1 (C), 141.9 (C), 171. 2 (C); *m/z* (ESI) 292.1335 (MH^+. $C_{19}H_{18}NO_2$ requires 292.1332).

(2*S*)-2-Amino-3-(2’’-fluorobiphen-4’-yl)propanoic acid hydrochloride (8e)

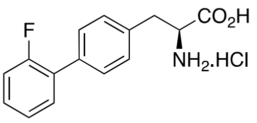

(2*S*)-2-Amino-3-(2’’-fluorobiphen-4’-yl)propanoic acid (**8e**) was synthesised according to general procedure 2 using methyl (2*S*)-2-(benzyloxycarbonylamino)-3-(2’’-fluorobiphen-4’-yl)propanoate (**7e**) (0.062 g, 0.15 mmol) and caesium carbonate (0.064 g, 0.20 mmol). Recrystallisation from methanol and diethyl ether gave (2*S*)-2-amino-3-(2’’-fluorobiphen-4’-yl)propanoic acid (**8e**) (0.026 g, 58%) as a white solid. Mp 197–202 °C; ν_{max}/cm^{-1} (neat) 2882 (CH), 1723 (C=O), 1483, 1252, 1183, 742; $[\alpha]_D^{17}$ +11.1 (*c* 0.1, MeOH); δ_H (400 MHz, CD_3OD) 3.21 (1H, dd, *J* 14.5, 7.9 Hz, 3-*H*H), 3.39 (1H, dd, *J* 14.5, 5.2 Hz 3-H*H*), 4.22–4.34 (1H, m, 2-H), 7.19 (1H, t, *J* 8.9 Hz, 5’’-H), 7.23–7.29 (1H, m, 6’’-H), 7.34–7.45 (3H, m, 2’-H, 6’-H and 4’’-H), 7.45–7.52 (1H, m, 3’’-H), 7.57 (2H, d, *J* 7.7 Hz, 3’-H and 5’-H); δ_C (101 MHz, CD_3OD) 37.1 (CH_2), 55.3 (CH), 117.0 (d, $^2J_{CF}$ 22.9 Hz, CH), 125.8 (d, $^3J_{CF}$ 3.7 Hz, CH), 129.7 (d, $^2J_{CF}$ 13.4 Hz, C), 130.5 (d, $^3J_{CF}$ 8.4 Hz, CH), 130.7 (2 × CH), 130.7 (d, $^4J_{CF}$ 3.0 Hz, 2 × CH), 131.8 (d, $^4J_{CF}$ 3.4 Hz, CH), 135.3 (C), 136.8 (C), 161.1 (d, $^1J_{CF}$ 246.4 Hz, C), 171.4 (C); *m/z* (ESI) 260.1082 (MH^+. $C_{15}H_{15}FNO_2$ requires 206.1081).

(2*S*)-2-Amino-3-(4’’-fluorobiphen-4’-yl)propanoic acid hydrochloride (8f)

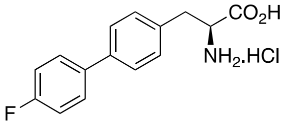

(2*S*)-2-Amino-3-(4’’-fluorobiphen-4’-yl)propanoic acid hydrochloride (**8f**) was synthesised according to general procedure 2 using methyl (2*S*)-2-(benzyloxycarbonylamino)-3-(4’’-fluorobiphen-4’-yl)propanoate (**7f**) (0.107 g, 0.263 mmol) and caesium carbonate (0.114 g, 0.350 mmol). Recrystallisation from methanol and diethyl ether gave (2*S*)-2-amino-3-(4’’-fluorobiphen-4’-yl)propanoic acid hydrochloride (**8f**) (0.041 g, 53%) as an off-white solid. Mp 288–292 °C (decomposed); ν_{max}/cm^{-1} (neat) 2895 (CH), 2162 (CH), 1720 (C=O), 1601 (C=C), 1494, 1232 1156, 812; $[\alpha]_D^{25}$ −5.4 (*c* 0.1, MeOH); δ_H (400 MHz, CD_3OD) 3.22 (1H, dd, *J* 14.6, 7.5 Hz, 3-*H*H), 3.36 (1H, dd, *J* 14.6, 5.4 Hz, 3-H*H*), 4.29 (1H, dd, *J* 7.5, 5.4 Hz, 2-H), 7.17 (2H, t, *J* 8.5 Hz, 3’’-H and 5’’-H), 7.39 (2H, d, *J* 7.9 Hz, 3’-H and 5’-H), 7.57–7.66 (4H, m, 2’-H, 6’-H, 2’’-H and 6’’-H); δ_C (101 MHz, CD_3OD) 36.9 (CH_2), 55.1 (CH), 116.6 (2 × CH, d, $^2J_{CF}$ 21.7 Hz), 128.6 (2 × CH), 129.7 (2 × CH, d, $^3J_{CF}$ 8.1 Hz), 131.1 (2 × CH), 134.7 (C), 138.2 (C), 141.0 (C), 164.0 (C, d, $^1J_{CF}$ 245.2 Hz), 171.2 (C); *m/z* (ESI) 260.1089 (MH^+. $C_{15}H_{15}FNO_2$ requires 260.1081).

(2*S*)-2-Amino-3-(4''-chlorobiphen-4'-yl)propanoic acid hydrochloride (8g)

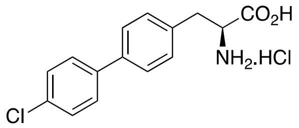

(2*S*)-2-Amino-3-(4''-chlorobiphen-4'-yl)propanoic acid hydrochloride (**8g**) was synthesised according to general procedure 2 using methyl (2*S*)-2-(benzyloxycarbonylamino)-3-(4''-chlorobiphen-4'-yl)propanoate (**7g**) (0.091 g, 0.27 mmol) and caesium carbonate (0.089 g, 0.27 mmol). Recrystallisation from methanol and diethyl ether gave (2*S*)-2-amino-3-(4''-methoxybiphen-4'-yl)propanoic acid (**8g**) (0.043 g, 64%) as a white solid. Mp 235–237 °C; ν_{max}/cm^{-1} (neat) 3457 (NH), 2883 (CH), 2176, 1730 (C=O), 1497, 1213; $[\alpha]_D^{23}$ –12.0 (*c* 0.1, MeOH); δ_H (400 MHz, CD_3OD) 3.20 (1H, dd, *J* 14.5, 7.5 Hz, 3-*H*H), 3.36 (1H, dd, *J* 14.5, 7.6 Hz, 3-H*H*), 4.27–4.30 (1H, m, 2-H), 7.37–7.53 (4H, m, 2'-H, 3'-H, 5' H and 6'-H), 7.58–7.68 (4H, m, 2''-H , 3''-H, 5''-H and 6''-H); δ_C (101 MHz, CD_3OD) 37.0 (CH_2), 55.1 (CH), 128.6 (2 × CH), 129.4 (2 × CH), 130.0 (2 × CH), 131.1 (2 × CH), 134.6 (C), 135.1 (C), 140.5 (C), 140.7 (C), 171.2 (C); *m/z* (ESI) 276.0794 (MH^+. $C_{15}H_{15}{}^{35}ClNO_2$ requires 276.0786).

(2*S*)-2-Amino-3-[(4''-trifluoromethyl)biphen-4'-yl]propanoic acid hydrochloride (8h)

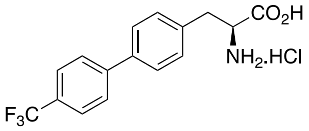

(2*S*)-2-Amino-3-[(4''-trifluoromethyl)biphen-4'-yl]propanoic acid (**8h**) was synthesised according to general procedure 2 using methyl (2*S*)-2-(benzyloxycarbonylamino)-3-[(4''-(trifluoromethyl)biphen-4'-yl]propanoate (**7h**) (0.100 g, 0.219 mmol) and caesium carbonate (0.0930 g, 0.284 mmol). Recrystallisation from methanol and diethyl ether gave (2*S*)-2-amino-3-[(4''-trifluoromethyl)biphen-4'-yl]propanoic acid (**8h**) (0.0612 g, 82%) as a white solid. Mp 260–263 °C; ν_{max}/cm^{-1} (neat) 2871 (CH), 1781 (C=O), 1730 (C=O), 1616, 1484, 1321, 1124, 1071, 811; $[\alpha]_D^{16}$ +37.2 (*c* 0.1, MeOH); δ_H (400 MHz, CD_3OD) 3.23 (1H, dd, *J* 14.5, 7.7 Hz, 3-*H*H), 3.39 (1H, dd, *J* 14.5, 5.4 Hz 3-H*H*), 4.30 (1H, dd, *J* 7.7, 5.4 Hz, 2-H), 7.45 (2H, d, *J* 8.2 Hz, 2'-H and 6'-H), 7.69–7.79 (4H, m, 3'-H, 5'-H, 2''-H and 6''-H), 7.83 (2H, d, *J* 7.9 Hz, 3''-H and 5''-H); δ_C (101 MHz, CD_3OD) 37.1 (CH_2), 55.2 (CH), 125.8 (q, $^1J_{CF}$ 270.6 Hz, C), 126.8 (q, $^3J_{CF}$ 3.9 Hz, 2 × CH), 128.5 (2 × CH), 128.9 (2 × CH), 130.5 (q, $^2J_{CF}$ 32.8 Hz, C), 131.2 (2 × CH), 135.9 (C), 140.4 (C), 145.6 (C), 171.3 (C); *m/z* (ESI) 310.1049 (MH^+. $C_{16}H_{15}F_3NO_2$ requires 310.1049).

(2*S*)-2-Amino-3-(4''-acetylbiphen-4'-yl)propanoic acid hydrochloride (8i)

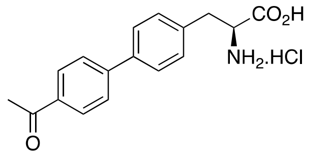

(2*S*)-2-Amino-3-(4''-acetylbiphen-4'-yl)propanoic acid hydrochloride (**8i**) was synthesised according to general procedure 2 using methyl (2*S*)-2-(benzyloxycarbonylamino)-3-(biphen-4'-yl)propanoate (**7i**) (0.052 g, 0.12 mmol) and caesium carbonate (0.051 g, 0.16 mmol). Recrystallisation from methanol and diethyl ether gave (2*S*)-2-amino-3-(4''-acetylbiphen-4'-yl)propanoic acid (**8i**) (0.030 g, 77%) as a white solid. Mp 230–234 °C; ν_{max}/cm^{-1} (neat) 3480 (NH), 2888 (CH), 1762 (C=O), 1645, 1551, 1479, 1294, $[\alpha]_D^{23}$ –18.0 (*c* 0.1, MeOH); δ_H (400 MHz, CD_3OD) 2.64 (3H, s, 4''-$COCH_3$), 3.22 (1H, dd, *J* 14.5, 7.6 Hz, 3-*H*H), 3.38 (1H, dd, *J* 14.5, 5.4 Hz, 3-H*H*), 4.31 (1H, dd, *J* 7.6, 5.4 Hz, 2-H), 7.44 (2H, d, *J* 8.0 Hz, 2'-H and 6'-H), 7.72 (2H, d, *J* 8.0 Hz, 3'-H and 5'-H), 7.78 (2H, d, *J* 8.4 Hz, 2''-H and 6''-H), 8.08 (2H, d, *J* 8.4 Hz, 3''-H and 5''-H); δ_C (101 MHz, CD_3OD) 26.7 (CH_3), 37.0 (CH_2), 55.0 (CH), 128.1 (2 × CH), 128.9 (2 × CH), 130.2 (2 × CH), 131.2 (2 × CH), 135.8 (C), 137.3 (C), 140.6 (C), 146.5 (C), 171.2 (C), 200.1 (C); *m/z* (ESI) 284.1287 (MH^+. $C_{17}H_{18}NO_3$ requires 284.1281).

(2*S*)-2-Amino-3-(2''-methoxybiphen-4'-yl)propanoic acid hydrochloride (8j)

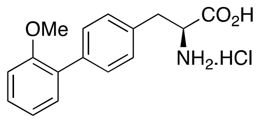

(2*S*)-2-Amino-3-(2''-methoxybiphen-4'-yl)propanoic acid hydrochloride (**8j**) was synthesised according to general procedure 2 using methyl (2*S*)-2-(benzyloxycarbonylamino)-3-(2''-methoxybiphen-4'-yl)propanoate (**7j**) (0.118 g, 0.291 mmol) and caesium carbonate (0.119 g, 0.365 mmol). Recrystallisation from methanol and diethyl ether gave (2*S*)-2-amino-3-(2''-methoxybiphen-4'-yl)propanoic acid hydrochloride (**8j**) (0.046 g, 52%) as a white solid. Mp 215–218 °C; ν_{max}/cm^{-1} (neat) 2912 (CH), 2208, 1724 (C=O), 1594 (C=C), 1483, 1232, 751; $[\alpha]_D^{24}$ +7.8 (*c* 0.1, MeOH); δ_H (400 MHz, CD_3OD) 3.20 (1H, dd, *J* 14.5, 7.9 Hz, 3-*H*H), 3.38 (1H, dd, *J* 14.5, 5.0 Hz, 3-H*H*), 3.78 (3H, s, OCH_3), 4.28 (1H, dd, *J* 7.9, 5.0 Hz, 2-H), 7.00 (1H, t, *J* 7.9 Hz, 5''-H), 7.06 (1H, d, *J* 8.3 Hz, 3''-H), 7.22–7.37 (4H, m, 2'-H, 6'-H, 4''-H and 6''-H), 7.49 (2H, d, *J* 7.8 Hz, 3'-H and 5'-H); δ_C (101 MHz, CD_3OD) 37.0 (CH_2), 55.3 (CH), 56.0 (CH_3), 112.6 (CH), 121.9 (CH), 130.0 (CH), 130.1 (2 × CH), 131.26 (2 × CH), 131.32 (CH), 131.5 (C), 134.0 (C), 139.7 (C), 157.9 (C), 171.4 (C); *m/z* (ESI) 272.1291 (MH^+. $C_{16}H_{18}NO_3$ requires 272.1281).

(2*S*)-2-Amino-3-(4’’-methoxybiphen-4’-yl)propanoic acid hydrochloride (8k)

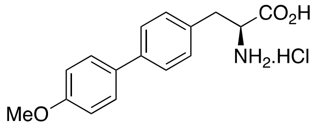

(2*S*)-2-Amino-3-(4’’-methoxybiphen-4’-yl)propanoic acid (**8k**) was synthesised according to general procedure 2 using methyl (2*S*)-2-(benzyloxycarbonylamino)-3-(4’’-methoxybiphen-4’-yl)propanoate (**7k**) (0.122 g, 0.290 mmol) and caesium carbonate (0.123 g, 0.378 mmol). Recrystallisation from methanol and diethyl ether gave (2*S*)-2-amino-3-(4’’-methoxybiphen-4’-yl)propanoic acid (**8k**) (0.0636 g, 71%) as a white solid. Mp 249–254 °C; ν_{max}/cm^{-1} (neat) 3255 (NH), 2928 (CH), 2144, 1731 (C=O), 1605, 1495, 1248, 811; $[\alpha]_D^{18}$ +56.5 (*c* 0.1, MeOH); δ_H (400 MHz, CD_3OD) 3.19 (1H, dd, *J* 14.6, 7.9 Hz, 3-*H*H), 3.36 (1H, dd, *J* 14.6, 5.4 Hz, 3-H*H*), 3.84 (3H, s, 4’’-OCH_3), 4.28 (1H, dd, *J* 7.9, 5.4 Hz, 2-H), 6.97–7.04 (2H, m, 3’’-H and 5’’-H), 7.33–7.39 (2H, m, 3’-H and 5’-H), 7.52–7.64 (4H, m, 2’-H, 6’-H, 2’’-H and 6’’-H); δ_C (101 MHz, CD_3OD) 36.9 (CH_2), 55.1 (CH), 55.8 (CH_3), 115.3 (2 × CH), 128.2 (2 × CH), 128.9 (2 × CH), 130.9 (2 × CH), 133.8 (C), 134.2 (C), 141.7 (C), 160.9 (C), 171.2 (C); *m/z* (ESI) 272.1281 (MH^+. $C_{16}H_{18}NO_3$ requires 272.1281).

(2*S*)-2-Amino-3-(4’’-dimethylaminobiphen-4’-yl)propanoic acid hydrochloride (8l)

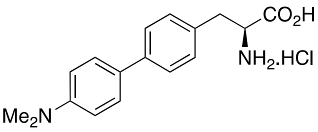

(2*S*)-2-Amino-3-(4’’-dimethylaminobiphen-4’-yl)propanoic acid hydrochloride (**8l**) was synthesised according to general procedure 2 using methyl (2*S*)-2-(benzyloxycarbonylamino)-3-(4’’-dimethylaminobiphen-4’-yl)propanoate (**7l**) (0.070 g, 0.16 mmol) and caesium carbonate (0.069 g, 0.21 mmol). Recrystallisation from methanol and diethyl ether gave (2*S*)-2-amino-3-(4’’-dimethylaminobiphen-4’-yl)propanoic acid hydrochloride (**8l**) (0.044 g, 95%) as a colourless solid. Mp 234–238 °C (decomposed); ν_{max}/cm^{-1} (neat) 2906 (CH), 2704 (OH), 2111, 1719 (C=O), 1498, 1400, 1231, 1139, 808; $[\alpha]_D^{15}$ +16.1 (*c* 0.1, MeOH); δ_H (400 MHz, CD_3OD) 3.23 (1H, dd, *J* 14.5, 7.4 Hz, 3-*H*H), 3.32 (6H, s, $N(CH_3)_2$), 3.36 (1H, dd, *J* 14.5, 5.7 Hz, 3-H*H*), 4.28–4.32 (1H, m, 2-H), 7.43 (2H, d, *J* 8.2 Hz, 3’’-H and 5’’-H), 7.67 (2H, d, *J* 8.2 Hz, 2’’-H and 6’’-H), 7.77 (2H, d, *J* 8.4 Hz, 3’-H and 5’-H), 7.85 (2H, d, *J* 8.4 Hz, 2’-H and 6’-H); δ_C (101 MHz, CD_3OD) 36.9 (CH_2), 47.2 (2 × CH_3), 55.0 (CH), 122.2 (2 × CH), 128.8 (2 × CH), 130.0 (2 × CH), 131.3 (2 × CH), 135.9 (C), 139.8 (C), 143.3 (C), 143.9 (C), 171.1 (C); *m/z* (APCI) 283.1455 ($[M–H]^-$. $C_{17}H_{19}N_2O_2$ requires 283.1452).

(2*S*)-2-Amino-3-[4'-phenyl(thiophen-3''-yl)]propanoic acid hydrochloride (8m)

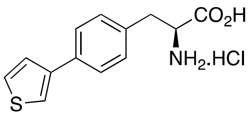

(2*S*)-2-Amino-3-[4'-phenyl(thiophen-3''-yl)]propanoic acid hydrochloride (**8m**) was synthesised as according to general procedure 2 using methyl (2*S*)-2-(benzyloxycarbonylamino)-3-[3''-phenyl-(thiophen-3''-yl)]propanoate (0.063 g, 0.16 mmol) and caesium carbonate (0.068 g, 0.21 mmol). Recrystallisation from methanol and diethyl ether gave (2*S*)-2-amino-3-[4'-phenyl(thiophen-3''-yl)]propanoic acid hydrochloride (**8m**) (0.016 g, 36%) as an off-white solid. Mp 238–240 °C (decomposed); ν_{max}/cm^{-1} (neat) 2908 (CH), 2191, 1731 (C=O), 1494, 1254, 772; $[\alpha]_D^{18}$ +35.2 (*c* 0.1, MeOH); δ_H (400 MHz, CD_3OD) 3.18 (1H, dd, *J* 14.6, 7.6 Hz, 3-*H*H), 3.35 (1H, dd, *J* 14.6, 4.8 Hz, 3-H*H*), 4.26 (1H, dd, *J* 7.6, 4.8 Hz, 2-H), 7.34 (2H, d, *J* 7.8 Hz, 3'-H and 5'-H), 7.43–7.52 (2H, m, ArH), 7.63–7.65 (1H, m, ArH), 7.68 (2H, d, *J* 7.8 Hz, 2'-H and 6'-H); δ_C (101 MHz, CD_3OD) 37.1 (CH_2), 55.2 (CH), 121.6 (CH), 127.0 (CH), 127.5 (CH), 128.0 (2 × CH), 131.0 (2 × CH), 134.3 (C), 136.9 (C), 142.9 (C), 171.3 (C); *m/z* (ESI) 248.0741 (MH^+. $C_{13}H_{14}NO_2S$ requires 248.0740).

(2*S*)-2-[(9*H*-Fluoren-9-ylmethoxycarbonyl)amino]-3-(4''-dimethylaminobiphen-4'-yl)propanoic acid (9)

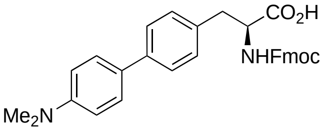

(2*S*)-2-Amino-3-(4''-dimethylaminobiphen-4'-yl)propanoic acid hydrochloride (**8l**) (0.150 g, 0.470 mmol) was dissolved in dioxane (1.5 mL) and water (1.5 mL). Sodium hydrogen carbonate (0.160 g, 0.474 mmol) and *N*-(9-fluorenylmethoxycarbonyloxy)succinimide (0.159 g, 1.88 mmol) were added and the reaction mixture was stirred at 25 °C for 24 h. The reaction mixture was concentrated *in vacuo*. The reaction mixture was diluted in water (20 mL) and acidified to pH 2 using 1 M hydrochloric acid and extracted with ethyl acetate (3 × 20 mL). The combined organic layers were dried ($MgSO_4$), filtered and concentrated *in vacuo*. Purification by flash column chromatography, eluting with 10% methanol in acetone afforded (2*S*)-2-[(9*H*-fluoren-9-ylmethoxycarbonyl)amino]-3-(4''-dimethylaminobiphen-4'-yl)propanoic acid (**9**) (0.115 g, 49%) as a white solid. Mp 212–215 °C; ν_{max}/cm^{-1} (neat) 3322 (NH), 2921 (CH), 2360, 1686 (C=O), 1609 (C=C), 1502, 1033, 809; $[\alpha]_D^{15}$ +46.6 (*c* 0.1, DMSO); δ_H (400 MHz, DMSO-d_6) 2.90 (6H, s, $N(CH_3)_2$), 2.91–3.00 (1H, m, 3-*H*H), 3.11–3.17 (1H, m, 3-H*H*), 3.96–4.21 (3H, m, 2-H, OC*H*HCH and OCH_2CH), 4.26–4.36 (1H, m, OCH*H*CH), 6.74 (2H, d, *J* 8.8 Hz, ArH), 7.19 (2H, d, *J* 8.0 Hz, ArH), 7.24–7.42 (8H, m, ArH), 7.58–7.66 (2H, m, ArH), 7.87 (2H, d, *J* 6.4 Hz, ArH); ^{13}C data unavailable due to compound decomposition in DMSO; *m/z* (ESI) 507.2296 (MH^+. $C_{32}H_{31}N_2O_4$ requires 507.2278).

3. Photophysical Data for α-Amino Acids 8a–m

Absorption and emission data were recorded using the following instruments:

1. UV-Vis spectra were recorded on a Perkin Elmer Lamda 25 instrument. Fluorescence spectra were recorded on a Shimadzu RF-5301PC spectrofluorophotometer. Emission data were measured using excitation and emission bandpass filters of 3 nm.

2. Both UV-Vis spectra and fluorescence spectra were recorded on a Horiba Duetta Fluorescence and Absorbance spectrometer. Absorbance spectra were recorded with an integration time of 0.05 s, and a band pass of 5 nm. Fluorescence spectra were recorded with and excitation and emission band pass of 5 nm, an integration time of 1 s or 2 s, and with detector accumulations set to 1.

Quantum yields were determined using L-tryptophan (Φ = 0.14 in water) as the standard reference.[3] The integrated fluorescence intensity of each compound was determined from the emission spectra given. Measurements were performed at five different concentrations. Concentrations were chosen to ensure the absorption value was below 0.1 to avoid re-absorption effects. Integrated fluorescence intensity was plotted as a function of the measured absorbance and a linear fit was calculated. The resultant gradient was then used to calculate the quantum yield, using the equation below:

$$\phi_x = \phi_{ST}\left(\frac{Grad_{ST}}{Grad_x}\right)\left(\frac{\eta_x^2}{\eta_{ST}^2}\right)$$

Subscript *ST* signifies the quantities associated with the quantum yield standard. Subscript X signifies the quantities associated with the novel compound. $Grad_X$ is the determined gradient associated with the novel compound. $Grad_{ST}$ is the determined gradient associated with quantum yield standard. η is the refractive index of the solvent used in the fluorescence measurements. η = 1.333 for water, 1.361 for ethanol and 1.331 for methanol.

Table S1. Photophysical Data of α-Amino Acids 8a–m.

amino acid	λ_{Abs} (nm)[a]	ε (cm^{-1} M^{-1})	λ_{Em} (nm)[a]	Φ_F[b]	brightness (cm^{-1} M^{-1})
**8a**	254	24200	314	0.12	2930
**8b**	284	14100	341	0.83	11740
**8c**	282	10300	342	0.27	2800
**8d**	256, 289	30900	356	0.18	5562
**8e**	280	2900	310	0.19	540
**8f**	252	20000	321	0.11	2200
**8g**	261	32100	330	0.08	2568
**8h**	262	23300	312, 371	0.15	3490
**8i**	289	19700	323	0.002	394
**8j**	248, 288	13800	333	0.38	5240
**8k**	262	14100	328	0.24	3430
**8l**	300	17000	384	0.73	12460
**8m**	265	18100	320	0.11	1900

[a]Spectra were recorded at concentrations of 1–5 μM in methanol. [b]Quantum yields (Φ_F) were determined in methanol using anthracene and L-tryptophan as standards.

Absorption and Emission Spectra for 8a (5 μM in methanol). Excitation at 256 nm.

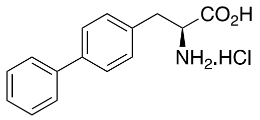

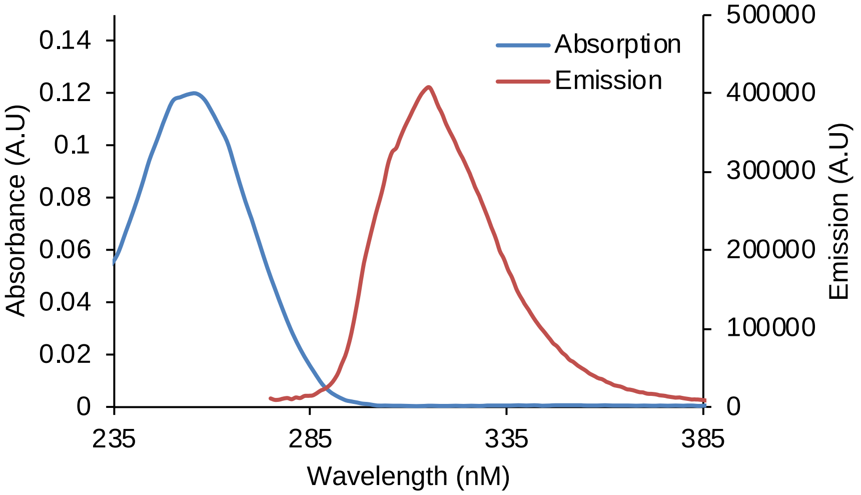

Absorption and Emission Spectra for 8b (1 µM in methanol). Excitation at 284 nm.

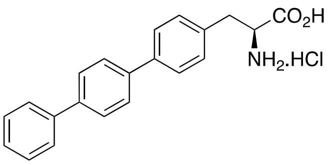

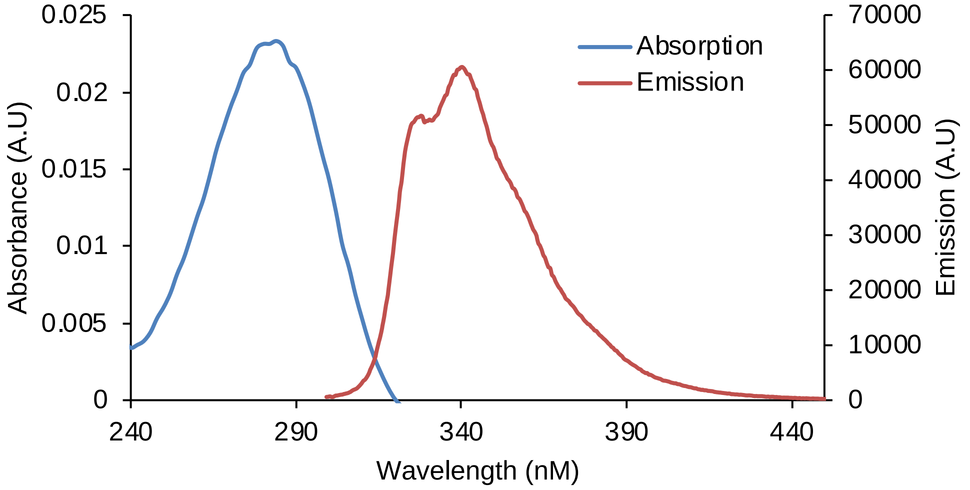

Absorption and Emission Spectra for 8c (2 µM in methanol). Excitation at 286 nm.

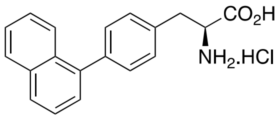

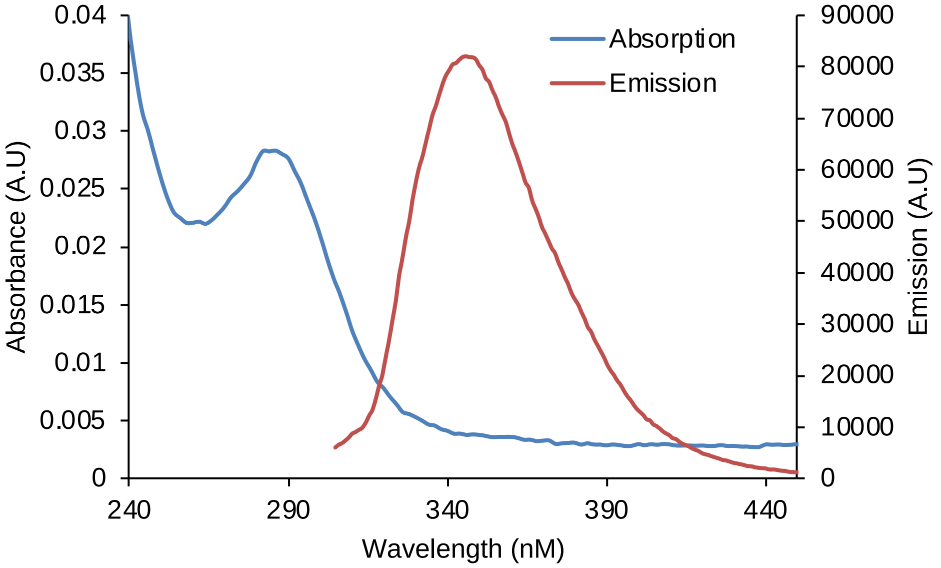

Absorption and Emission Spectra for 8d (4 µM in methanol). Excitation at 275 nm.

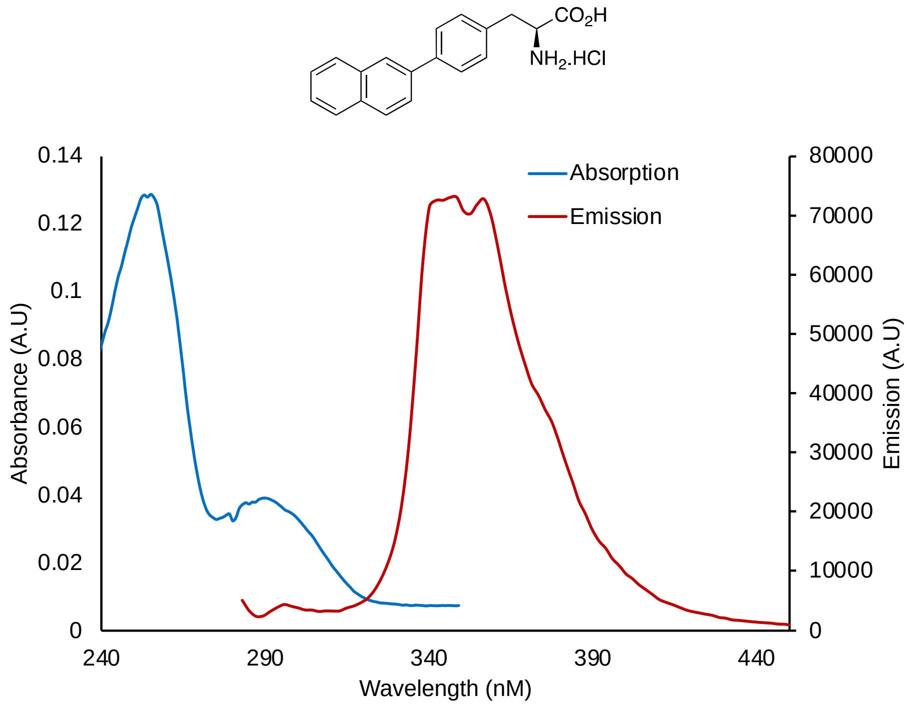

Absorption and Emission Spectra for 8e (2 µM in methanol). Excitation at 250 nm.

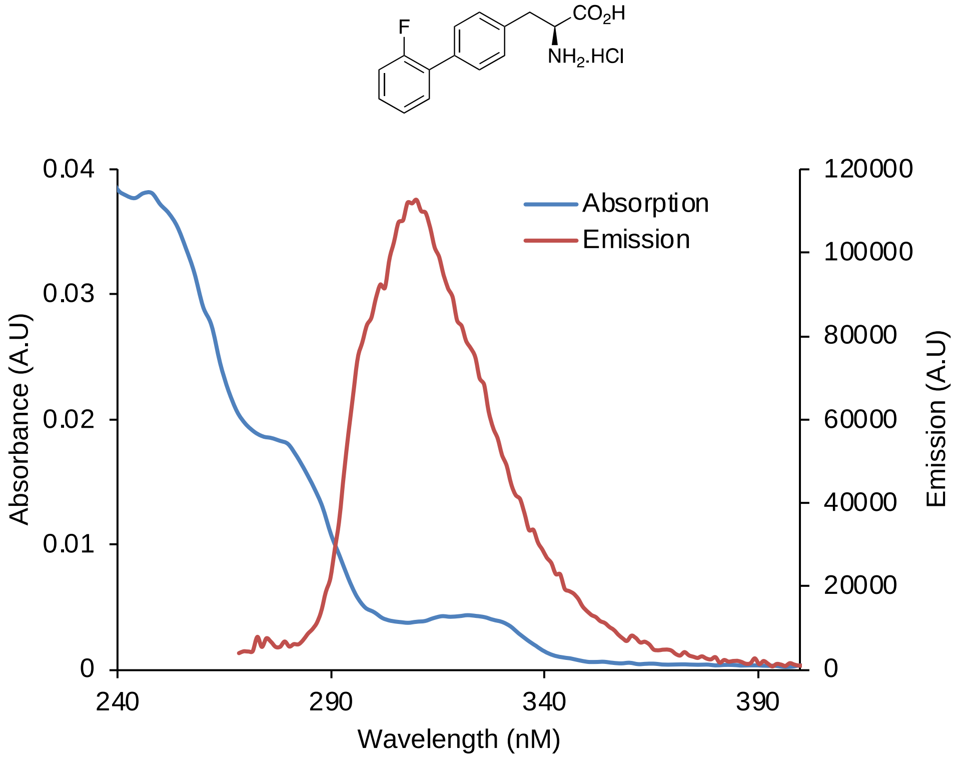

Absorption and Emission Spectra for 8f (5 µM in methanol). Excitation at 250 nm.

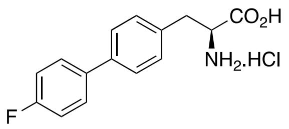

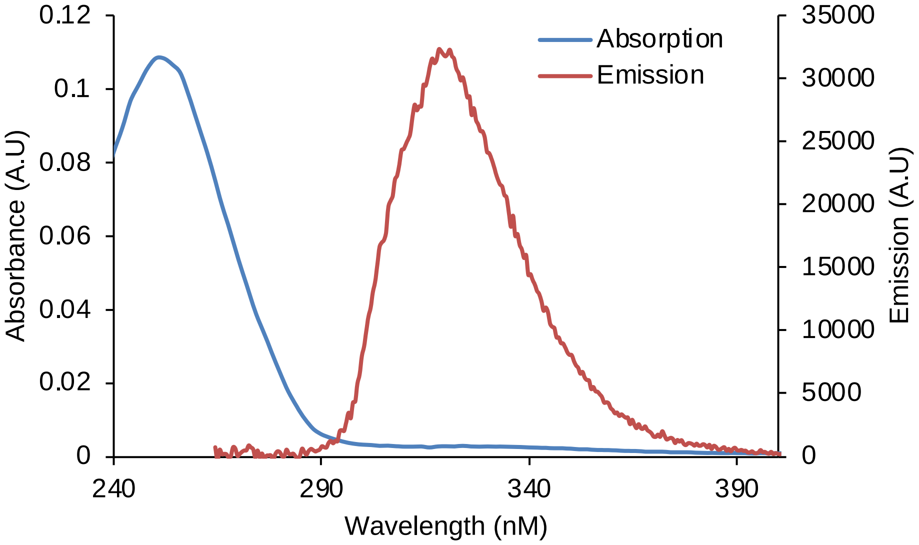

Absorption and Emission Spectra for 8g (4 µM in methanol). Excitation at 275 nm.

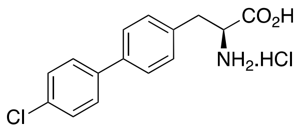

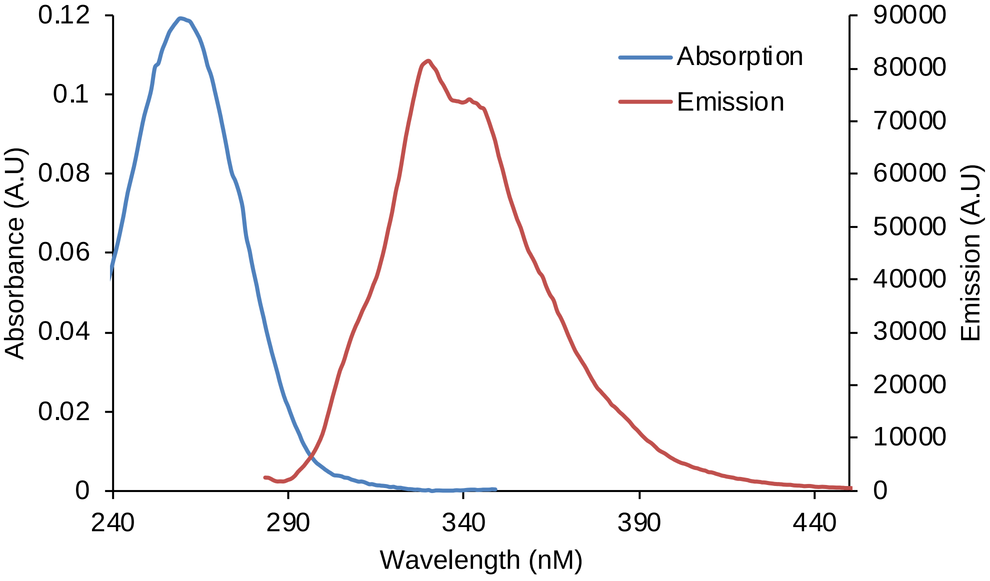

Absorption and Emission Spectra for 8h (2 µM in methanol). Excitation at 262 nm.

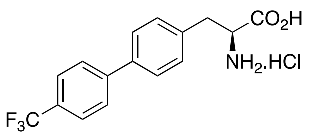

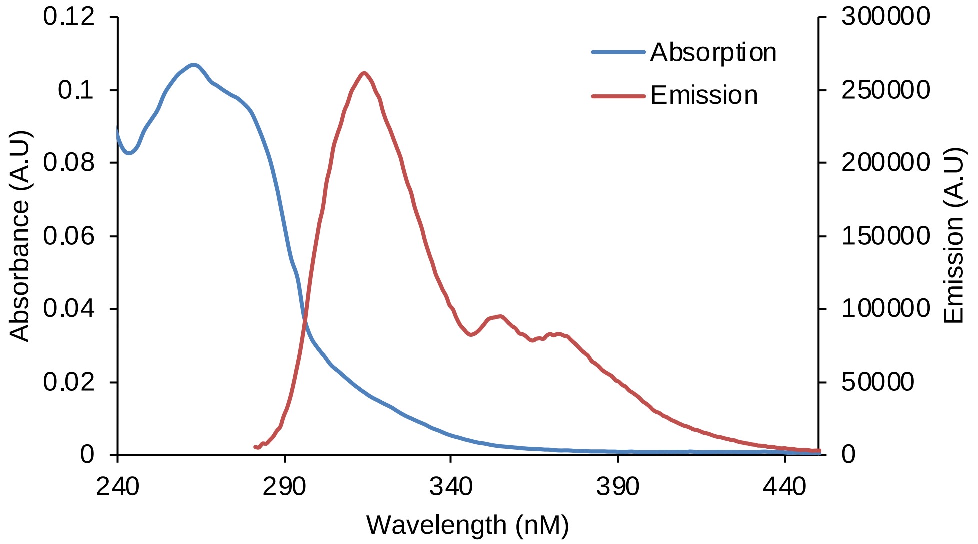

Absorption and Emission Spectra for 8i (5 µM in methanol). Excitation at 275 nm.

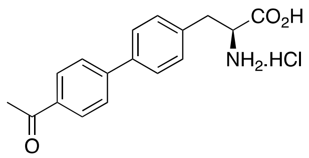

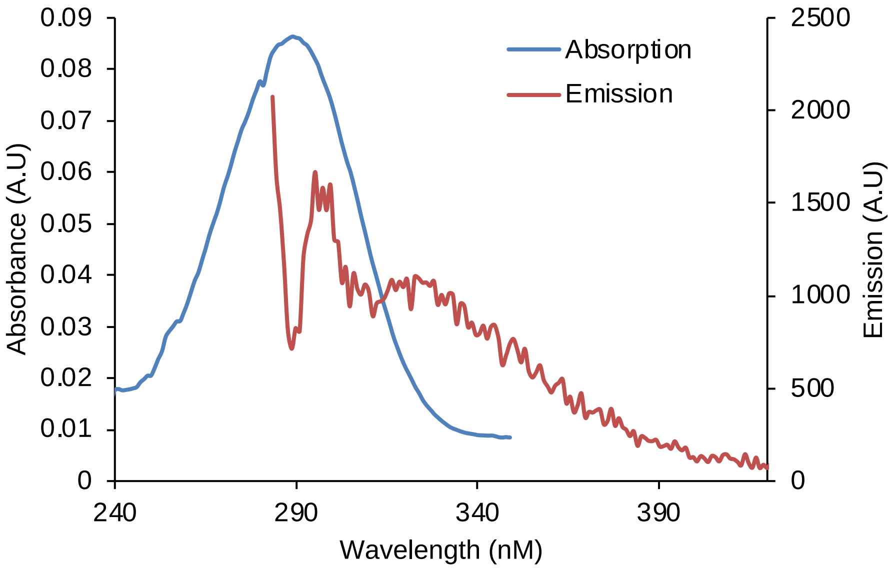

Absorption and Emission Spectra for 8j (5 μM in methanol). Excitation at 250 nm.

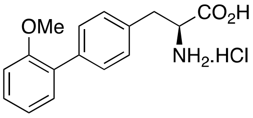

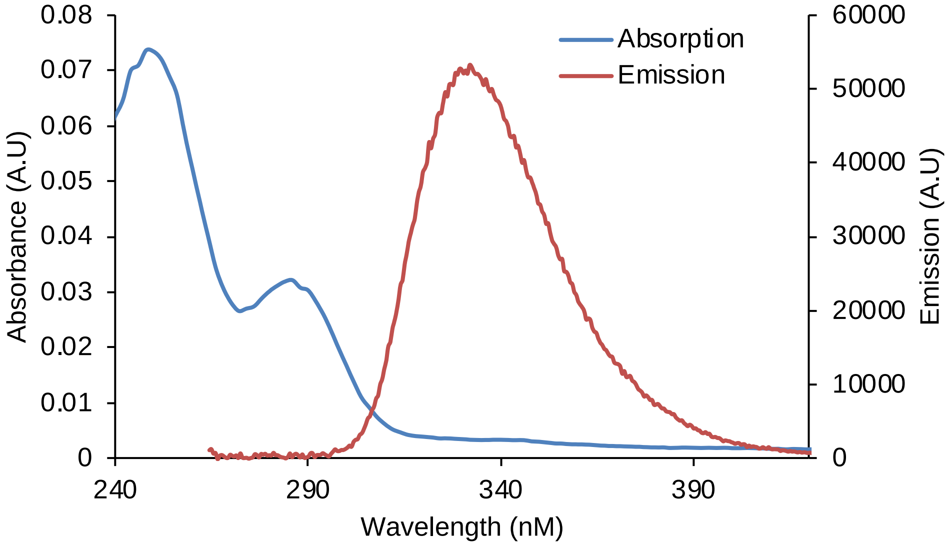

Absorption and Emission Spectra for 8k (5 μM in methanol). Excitation at 275 nm.

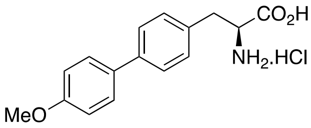

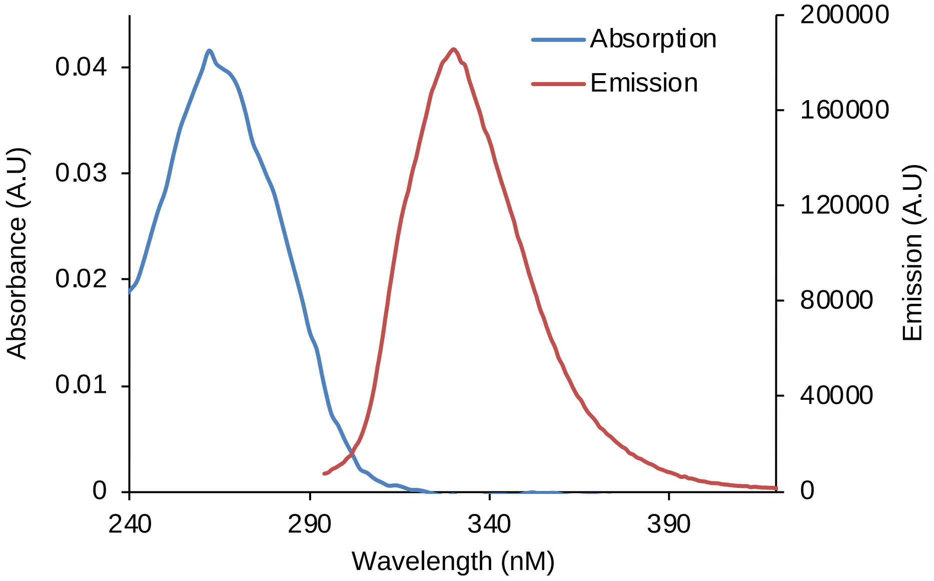

Absorption and Emission Spectra for 8l (5 µM in methanol). Excitation at 300 nm.

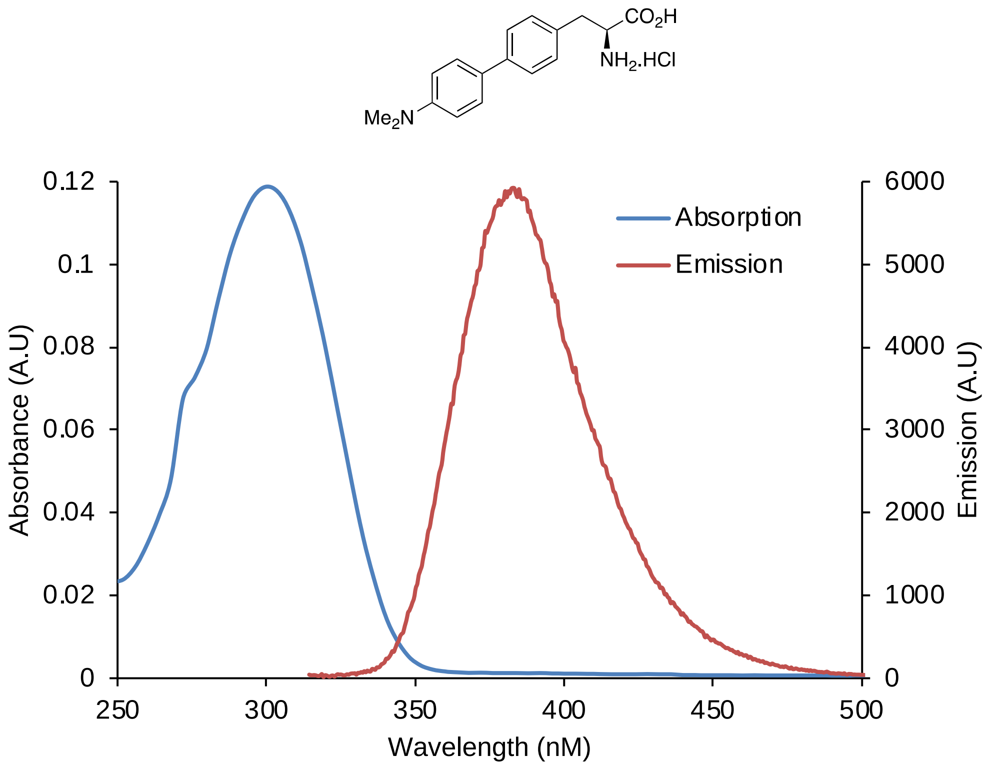

Absorption and Emission Spectra for 8m (5 µM in methanol). Excitation at 265 nm.

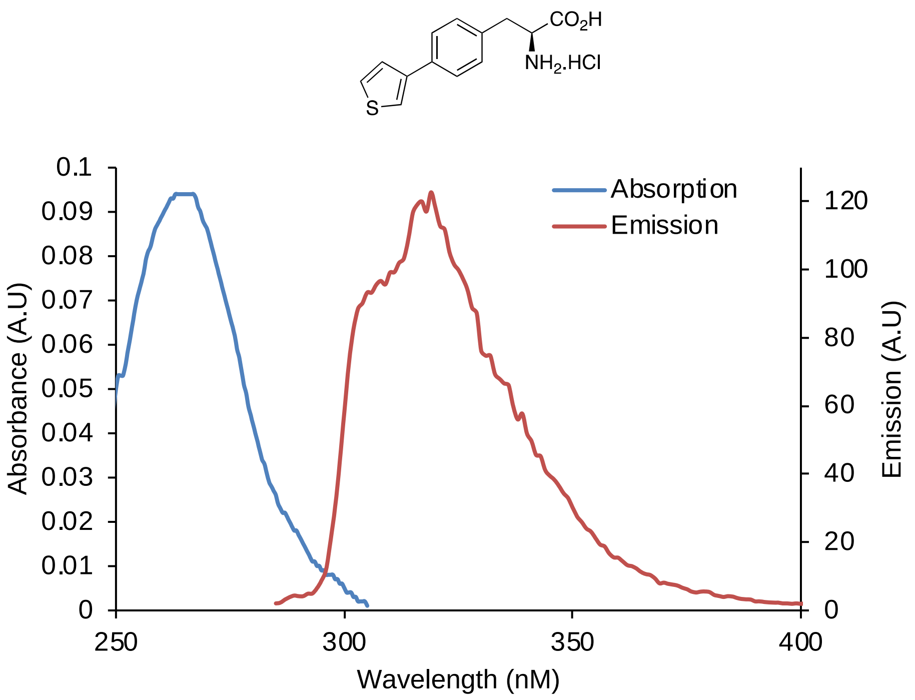

Additional Photophysical Data for 8l.

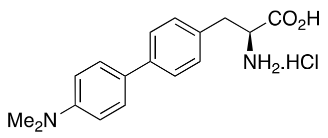

Solvatochromic Study (5 µM):

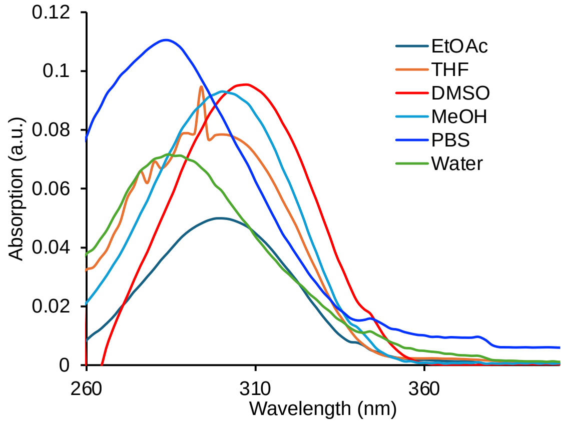

Emission spectra showing relative and normalised intensities: The quantum yields were also measured in a non-polar (EtOAC) and polar solvent (PBS). The quantum yields for both were found to be 0.13.

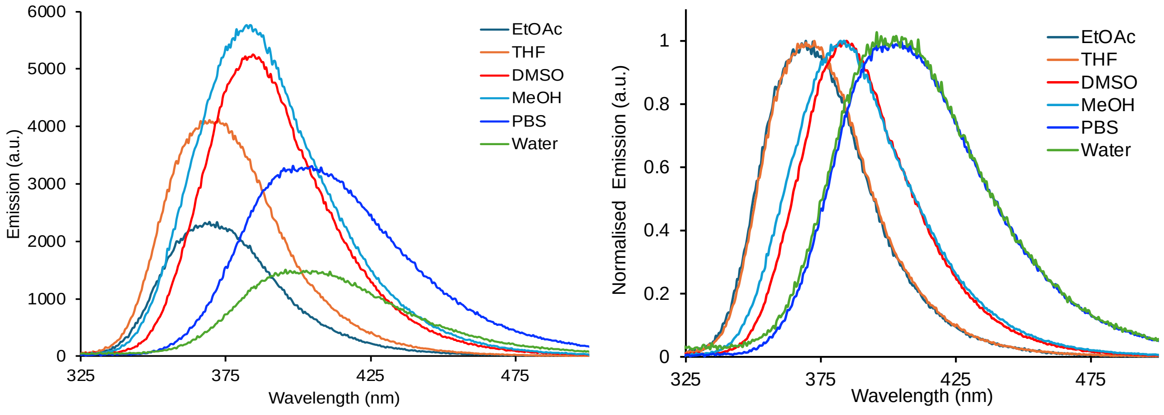

Lippert-Mataga Plot:

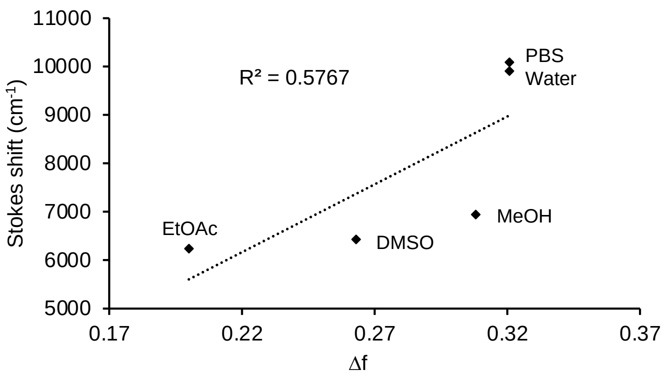

pH Study with 8l under acidic conditions (5 μM)

Absorption Spectrum:

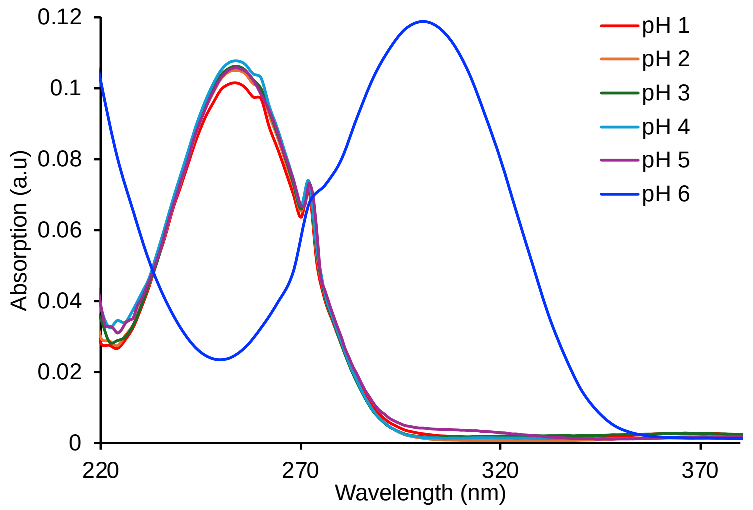

Emission Spectrum:

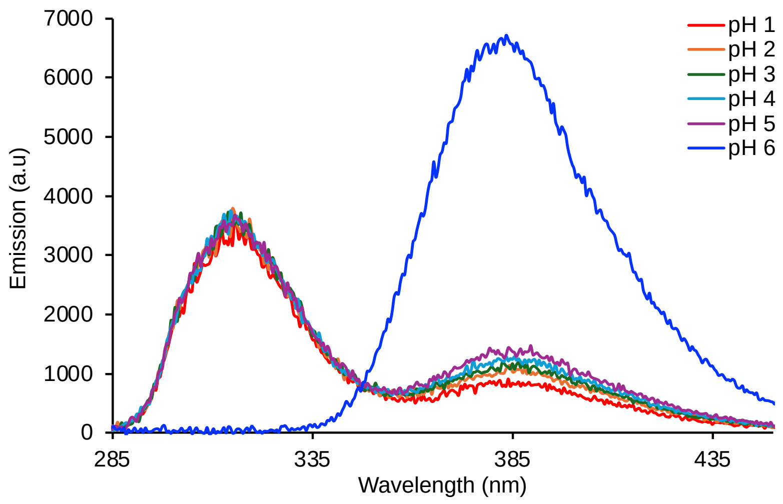

pH Study with 8l under neutral and basic conditions (using triethylamine, 5 μM)

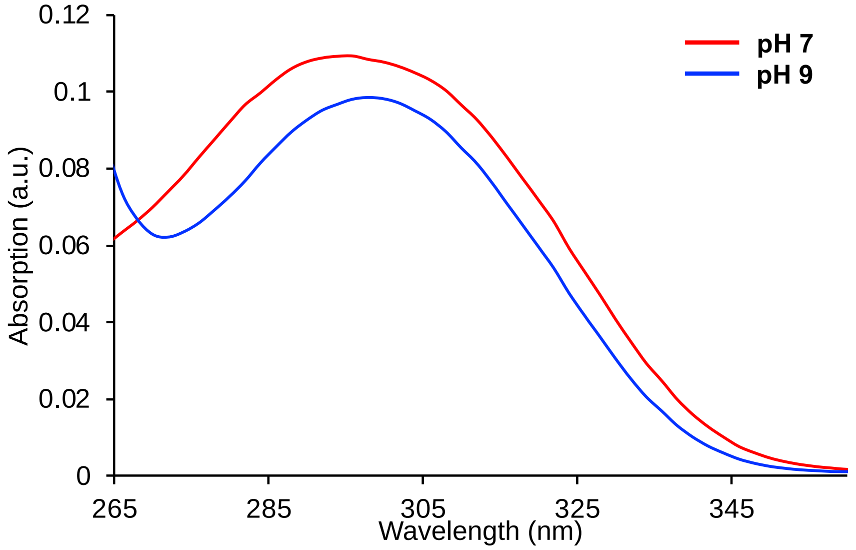

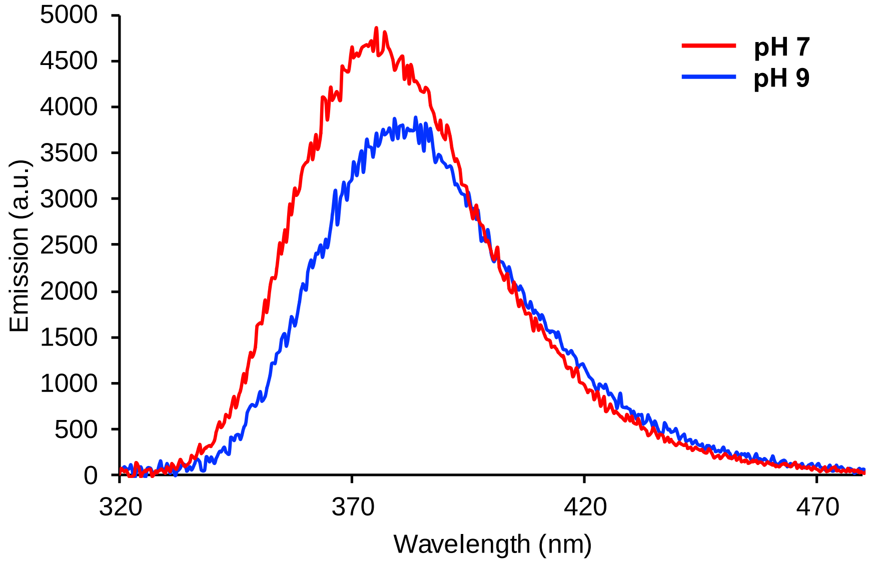

Viscosity Study with 8l (5 μM)

Absorption Spectra:

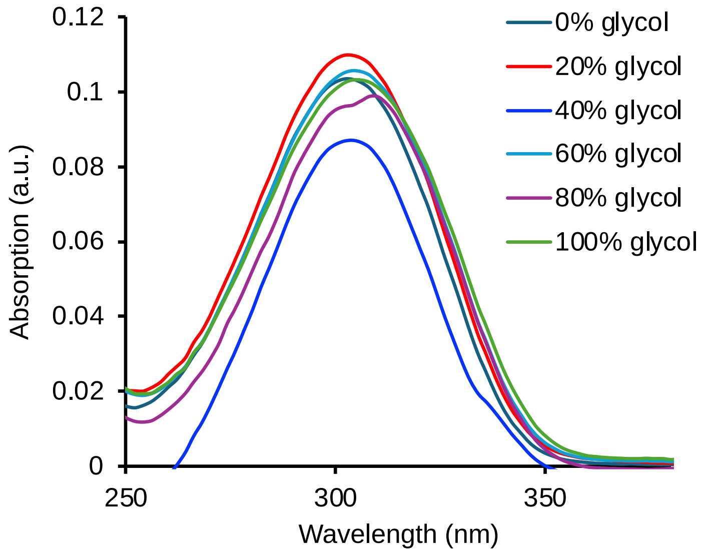

Emission Spectra:

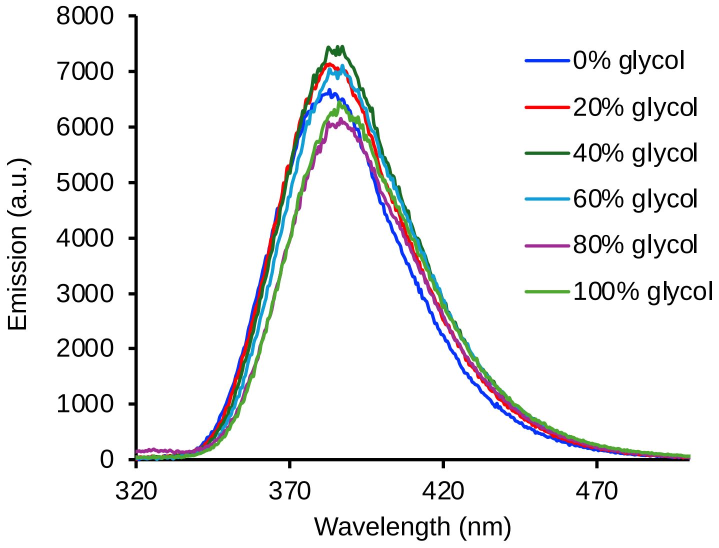

Aggregation Study with 8l

Absorption Spectra:

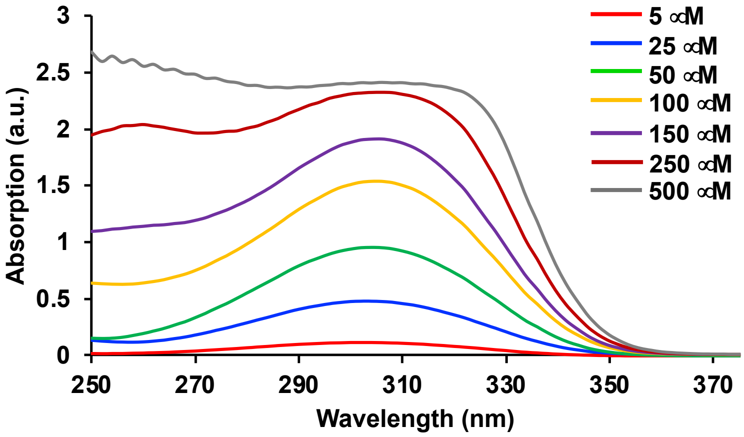

Emission Spectra:

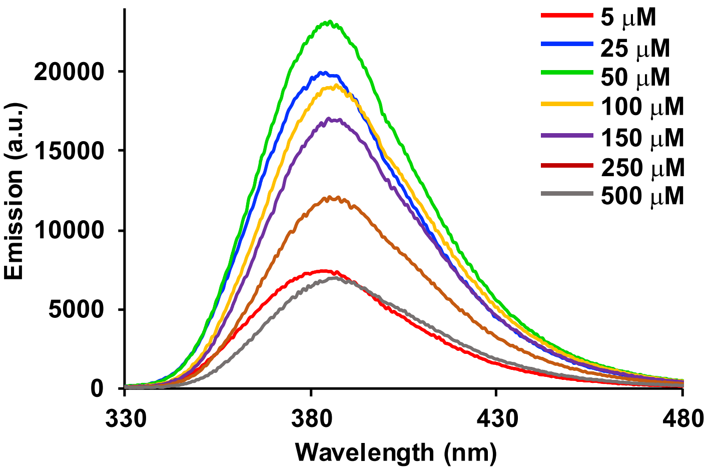

4. Computational Data

Quantum mechanical calculations were performed in Gaussian 5. The structure of amino acid **8l** was fully optimised (no imaginary frequencies) using density functional theory (DFT) at the B3LYP/6-31G(d,p) level.[4] HOMO and LUMO visualisation were achieved following full population analysis.

Optimised structure and total energy of **8l**:

E = −919.79494259

C −0.4171000000 0.2537000000 0.0156000000

C 0.4347000000 −0.7981000000 0.3830000000

C 1.8133000000 −0.6024000000 0.4325000000

C 2.3573000000 0.6417000000 0.1139000000

C 1.5167000000 1.6921000000 −0.2537000000

C 0.1372000000 1.5021000000 −0.3020000000

C −1.8640000000 0.0512000000 −0.0359000000

C −2.3969000000 −1.1372000000 −0.5558000000

C −3.7763000000 −1.3273000000 −0.6051000000

C −4.6383000000 −0.3371000000 −0.1345000000

C −4.1158000000 0.8470000000 0.3852000000

C −2.7372000000 1.0429000000 0.4340000000

H 0.0091000000 −1.7796000000 0.6404000000

H 2.4731000000 −1.4324000000 0.7249000000

H 1.9424000000 2.6740000000 −0.5078000000

H −0.5221000000 2.3319000000 −0.5978000000

H −1.7203000000 −1.9186000000 −0.9334000000

H −4.1850000000 −2.2614000000 −1.0178000000

H −4.7927000000 1.6292000000 0.7591000000

H −2.3288000000 1.9762000000 0.8499000000

C 3.8815000000 0.8548000000 0.1679000000

H 4.1638000000 1.5951000000 −0.5513000000

H 4.1612000000 1.1837000000 1.1469000000

C 4.5969000000 −0.4708000000 −0.1522000000

H 4.1714000000 −0.9018000000 −1.0343000000

N 4.4337000000 −1.4002000000 0.9749000000

H 4.6331000000 −2.3317000000 0.6705000000

H 5.0637000000 −1.1475000000 1.7093000000

C 6.0956000000 −0.2026000000 −0.3835000000

O 6.7375000000 0.8828000000 0.2908000000

H 6.2861000000 1.0569000000 1.1199000000

O 6.7572000000 −0.9352000000 −1.1639000000

N −6.0932000000 −0.5407000000 −0.1863000000

C −6.6517000000 −0.4321000000 1.1691000000

H −6.6741000000 0.5961000000 1.4644000000

H −7.6460000000 −0.8274000000 1.1779000000

H −6.0416000000 −0.9860000000 1.8517000000

C −6.7007000000 0.4820000000 −1.0500000000

H −6.1752000000 0.5204000000 −1.9812000000

H −7.7262000000 0.2343000000 −1.2290000000

H −6.6430000000 1.4357000000 −0.5683000000

5. Peptide Synthesis

Analysis: High resolution mass spectrometry (HRMS) was performed on an Agilent 6200 series TOF/65000 series Q-TOF High Resolution Mass Spectrometer using ESI$^-$ mode.

Synthesis: Peptides were synthesised on a CEM Liberty Blue peptide synthesis instrument using the Fmoc/tBu protecting group strategy and standard DIC/OxymaPure activation, using Rink amide resin. Cleavage from the resin was performed using 95% TFA, 2.5% H_2O and 2.5% TIPS. Purification was performed on a Dionex P680 semi-preparative HPLC system using either a Phenomenex Luna C18, 5 µm, 150 × 10 mm column at a flow rate of 3 mL/min. Gradients were run using a binary solvent system consisting of solution A (H_2O + 0.1% TFA) and B (MeCN + 0.1% TFA). Reverse-phase HPLC analysis was performed on a Shimadzu system with a UV-Vis detector monitoring at 214 nm and 280 nm. The column used was a Phenomenex, Aeris, 5 µm, peptide XB-C18, 150 × 4.6 mm at a flow rate of 1 mL/min. Gradients were run using a binary solvent system consisting of solution A (5% MeCN in H_2O + 0.1% TFA) and B (5% H_2O in MeCN + 0.1% TFA). Peptide content was analysed on a Thermo Scientific NanoDrop One UV-Vis spectrophotometer.

H-8l-Gly-Gly-Leu-Ser-Lys-Ile-Val-Lys(dnp)-Gly-NH$_2$ (10)

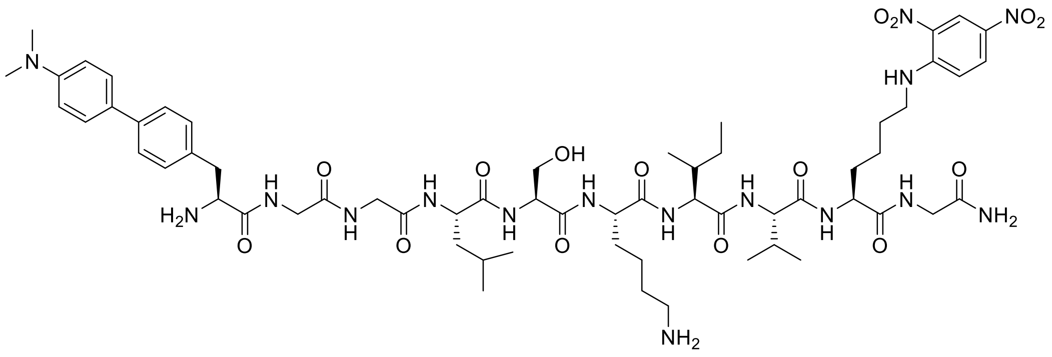

H-Gly-Gly-Leu-Ser(O^{t}Bu)-Lys(N_ε-Boc)-Ile-Val-Lys(dnp)-Gly-resin was synthesised through standard microwave-assisted SPPS, using Rink Amide MBHA resin (0.33 mmol/g) on a 0.1 mmol scale. After the synthesis was complete, the resin was washed with DMF (3 × 3 mL). (2*S*)-2-[(9*H*-Fluoren-9-ylmethoxycarbonyl)amino]-3-(4''-dimethylaminobiphen-4'-yl)propanoic acid (**9**) was subsequently incorporated *via* manual coupling to give peptide **10**. H-Gly-Gly-Leu-Ser(O^{t}Bu)-Lys(N_ε-Boc)-Ile-Val-Lys(dnp)-Gly-resin (0.05 mmol) was coupled to (2*S*)-2-[(9*H*-fluoren-9-ylmethoxycarbonyl)amino]-3-(4''-dimethylaminobiphen-4'-yl)propanoic acid (**9**) (38 mg, 0.075 mmol, 1.5 eq), DIPEA (26 µL, 0.15 mmol, 3 eq), PyBOP (52 mg, 0.1 mmol, 2 eq) in DMF (1.5 mL) for 3 h at room temperature. Fmoc-deprotection was achieved by suspending the resin in 20% morpholine in DMF (1 mL), which was gently mixed for 1 h. Full deprotection and cleavage was subsequently achieved with 95% TFA, 2.5%

water and 2.5% triisopropylsilane (5 mL total volume) which was added to the resin and mixed for 2 h. The reaction mixture was evaporated under a flow of nitrogen gas followed by precipitation of the peptide in cold diethyl ether (50 mL). The precipitate was dissolved in 1:1 H_2O/MeCN and lyophilised to give a yellow solid. The crude material was purified using a gradient of 10 to 60% solution B.

MS (ESI) *m/z*: $[M - H]^-$ Calcd for $C_{61}H_{91}N_{16}O_{15}$ 1287.9; Found 1287.9.

HPLC trace for peptide **10**, indicating retention time and purity:

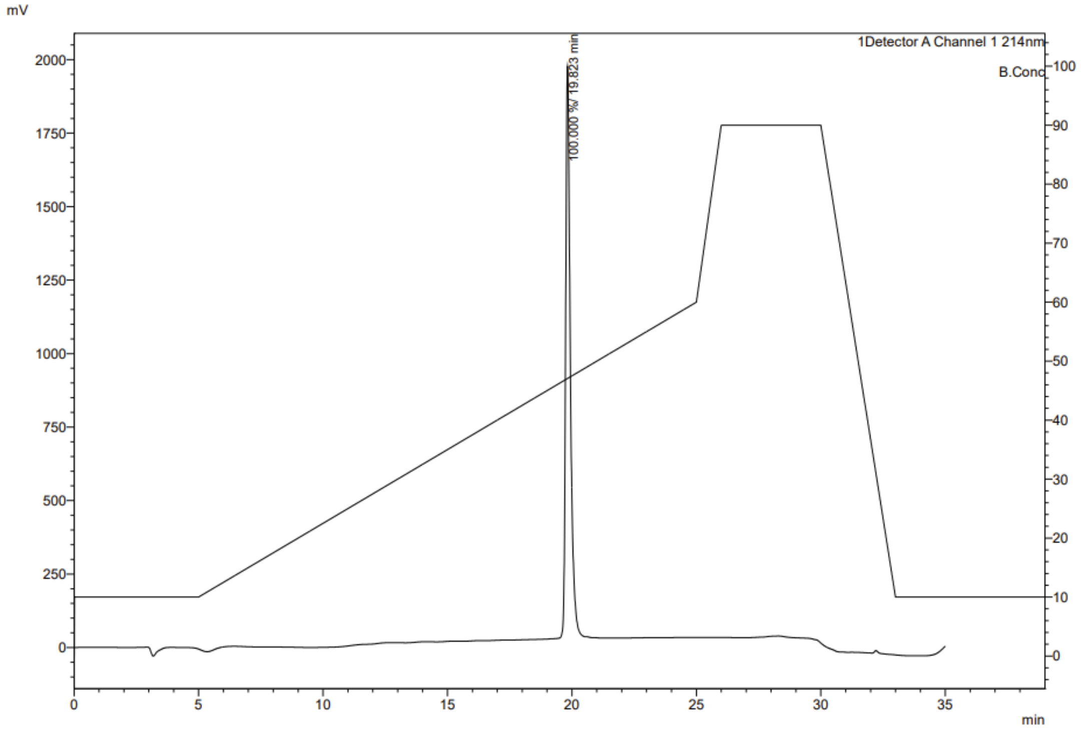

Retention time 19.8 min, purity >99% (20-minute gradient).

6. Determination of Förster Distance for Amino acid 8l/Lysine(dnp) Pair and Kinetic Parameters for Trypsin Digestion

Determination of Förster distance (R_0) for amino acid 8l/lysine(dnp) pair

Förster distance, R_0, was determined using the following equation[5]:

$$R_0 = 0.211[\kappa^2\, \eta^{-4} Q_D J(\lambda)]^{1/6}$$

where, κ^2 is a factor describing the relative orientation in space between two transition dipoles (typically assumed to be equal to 2/3); η is the refractive index of the medium and Q_D is the quantum yield of the donor in the absence of the acceptor. J(λ) is the overlap integral between the donor emission and acceptor absorption and can be calculated using the following equation:

$$J(\lambda) = \frac{\int_0^\infty F_D(\lambda)\varepsilon_A(\lambda)\lambda^4 d\lambda}{\int_0^\infty F_D(\lambda) d\lambda}$$

where λ is the wavelength and ε_A is the extinction coefficient of the acceptor. J(λ) can also be expressed by the following equation for calculating the spectral overlap in excel[6]:

$$J(\lambda) = \frac{\sum_{\lambda=300,\lambda\in N}^{600} F_D(\lambda)\varepsilon_A\lambda^4}{\sum_{\lambda=300,\lambda\in N}^{600} F_D(\lambda)}$$

Emission and absorption spectra of amino acid **8l** and Lys(dnp) were recorded in methanol at a concentration of 5 μM. Förster distance calculation was performed in excel using template provided by Hink and Visser.[7] This gave a Forster distance between the FRET pair of 36.45 Å.

Spectroscopic overlap between absorption of lysine(dnp) and emission of amino acid **8l**:

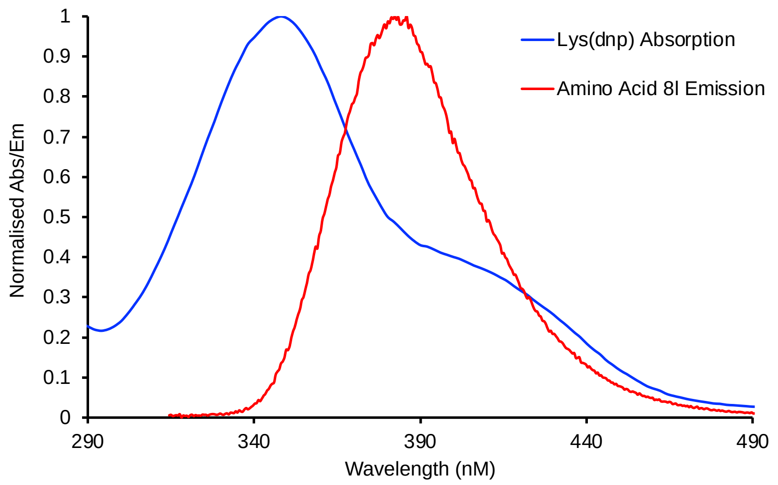

Determination of kinetic parameters for trypsin digestion of peptide 10

Kinetic measurements were performed using a Horiba Duetta Fluorescence and Absorbance spectrometer at 25 °C. A trypsin stock solution (8.11 μM) was prepared in 1 mM HCl and stored on ice. Solutions were prepared containing 3-morpholinopropanesulfonic acid (MOPS) buffer (20 mM, pH 7.0), trypsin (0.01 μM), various concentrations of peptide (0.50–2.50 μM) and water to give a total volume of 2 mL. Emission spectra were recorded at an excitation wavelength of 300 nm every 10 s over a 400 s time period. Emission data was repeated in triplicate for each concentration of peptide. To calculate the catalytic parameters K_M and k_{cat}, average emission intensity at 405 nm over time was plotted for each concentration. Initial velocity was calculated over the first 50 s using linear fitting in Origin. K_M and k_{cat} were subsequently calculated from Lineweaver-Burk plot of the reciprocal initial velocity versus peptide concentration. This gave a K_M values of 0.33 ± 0.073 μM and a k_{cat} value of 0.73 ± 0.057 s^{-1} (n = 3).

Increase in Emission over Time:

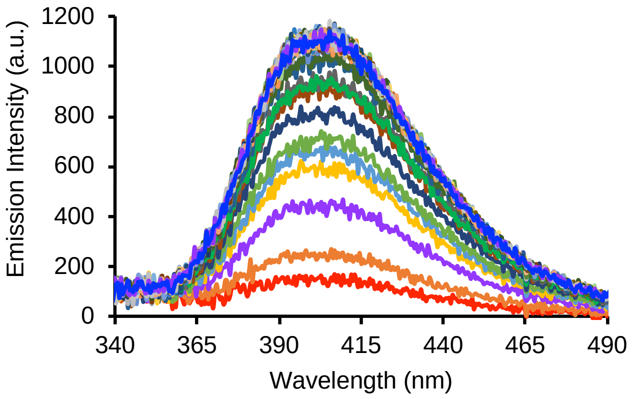

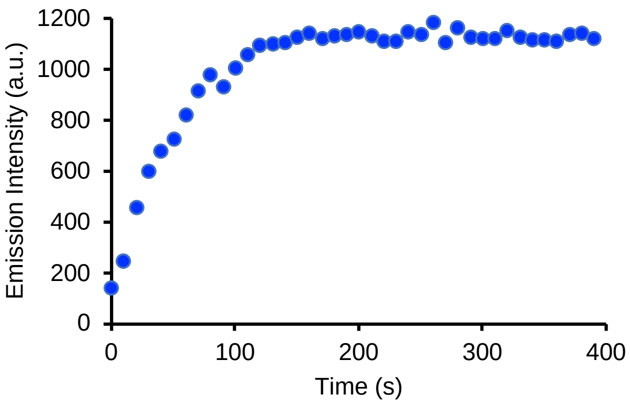

Lineweaver-Burk Plot:

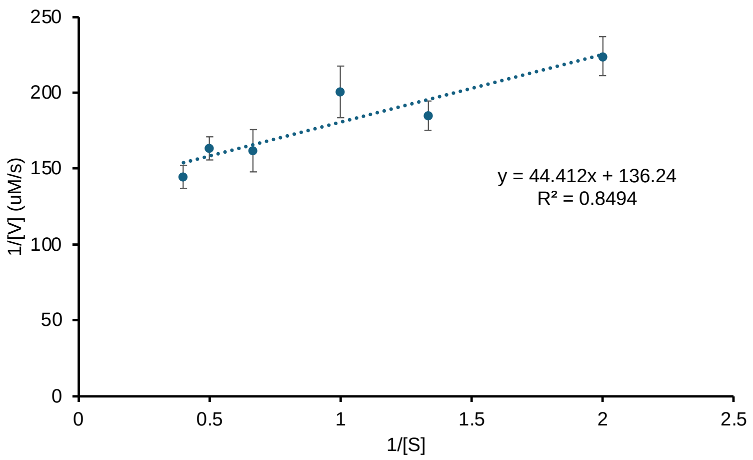

7. References

1. A. J. Ross, H. L. Lang and R. F. W. Jackson, *J. Org. Chem*., 2010, **75**, 245−248.

2. D. Georgiev, B. W. H. Saes, H. J. Johnston, S. K. Boys, A. Healy and A. N. Hulme, *Molecules*, 2016, **21**, 88−95.

3. A. T. R. Williams, S. A. Winfield and J. N. Miller, *Analyst*, 1983, **108**, 1067–1071.

4. W. J. Hehre, R. Ditchfield and J. A. Pople, *J. Chem. Phys*., 1972, **56**, 2257.

5. J. R. Lakowicz, Principles of Fluorescence Spectroscopy, Springer, New York, 2006.

6. M. Poreba, A. Szalek, W. Rut, P. Kasperkiewicz, I. Rutkowska-Wlodarczyk, S. J. Snipas, Y. Itoh, D. Turk, B. Turk, C. M. Overall, L. Kaczmarek, G. S. Salvesen and M. Drag, *Sci. Rep*., 2017, **7**, 43135.

7. Critical Transfer Distance Determination Between FRET Pairs, http://photobiology.info/Experiments/Biolum-Expt.html (accessed September 2024).

8. ^{1}H and $^{13}C\{^{1}H\}$ NMR Spectra for all Compounds

^{1}H NMR (400 MHz, $CDCl_3$)

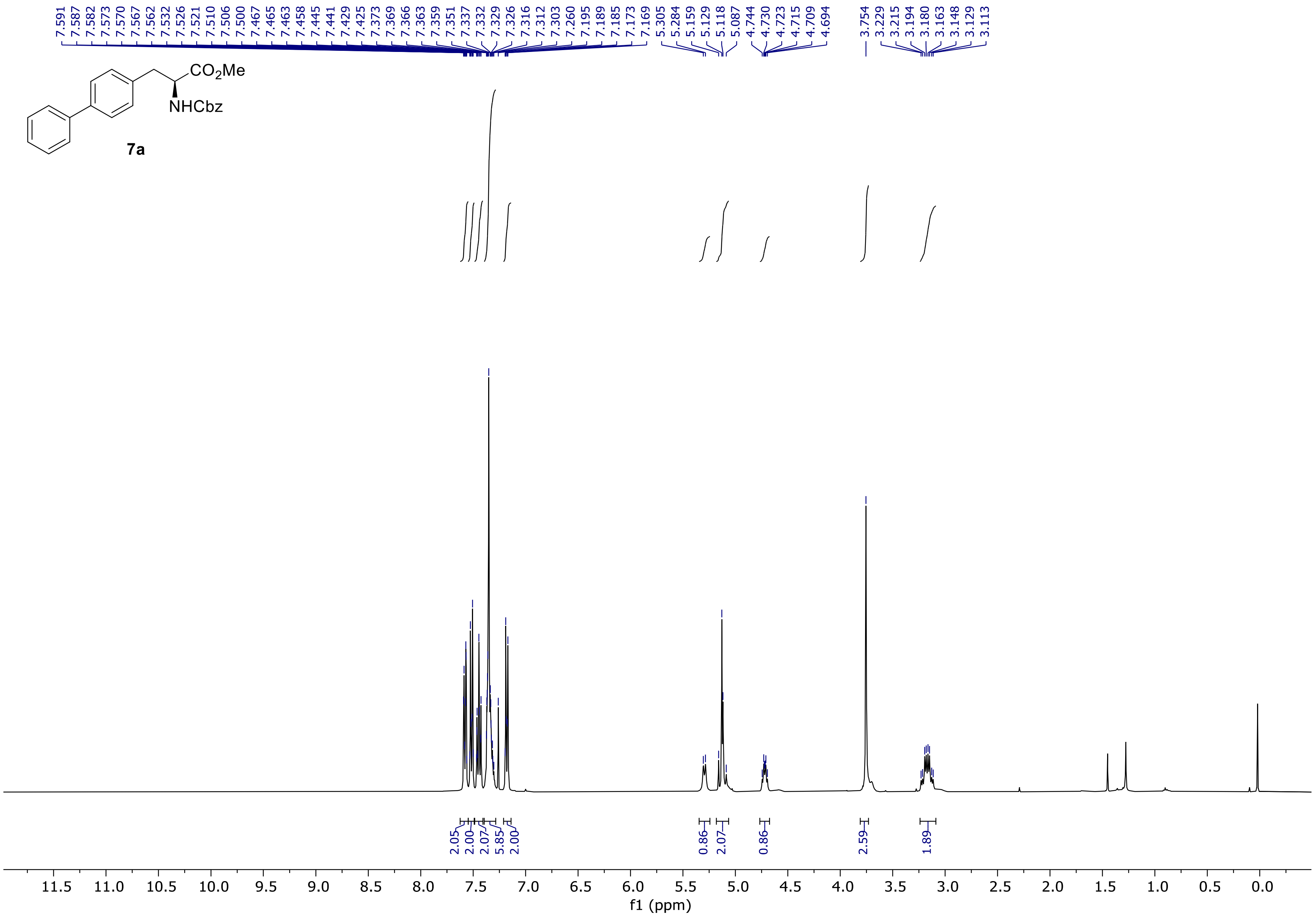

$^{13}C\{^1H\}$ NMR (101 MHz, $CDCl_3$)

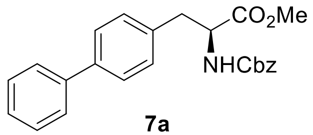

7a

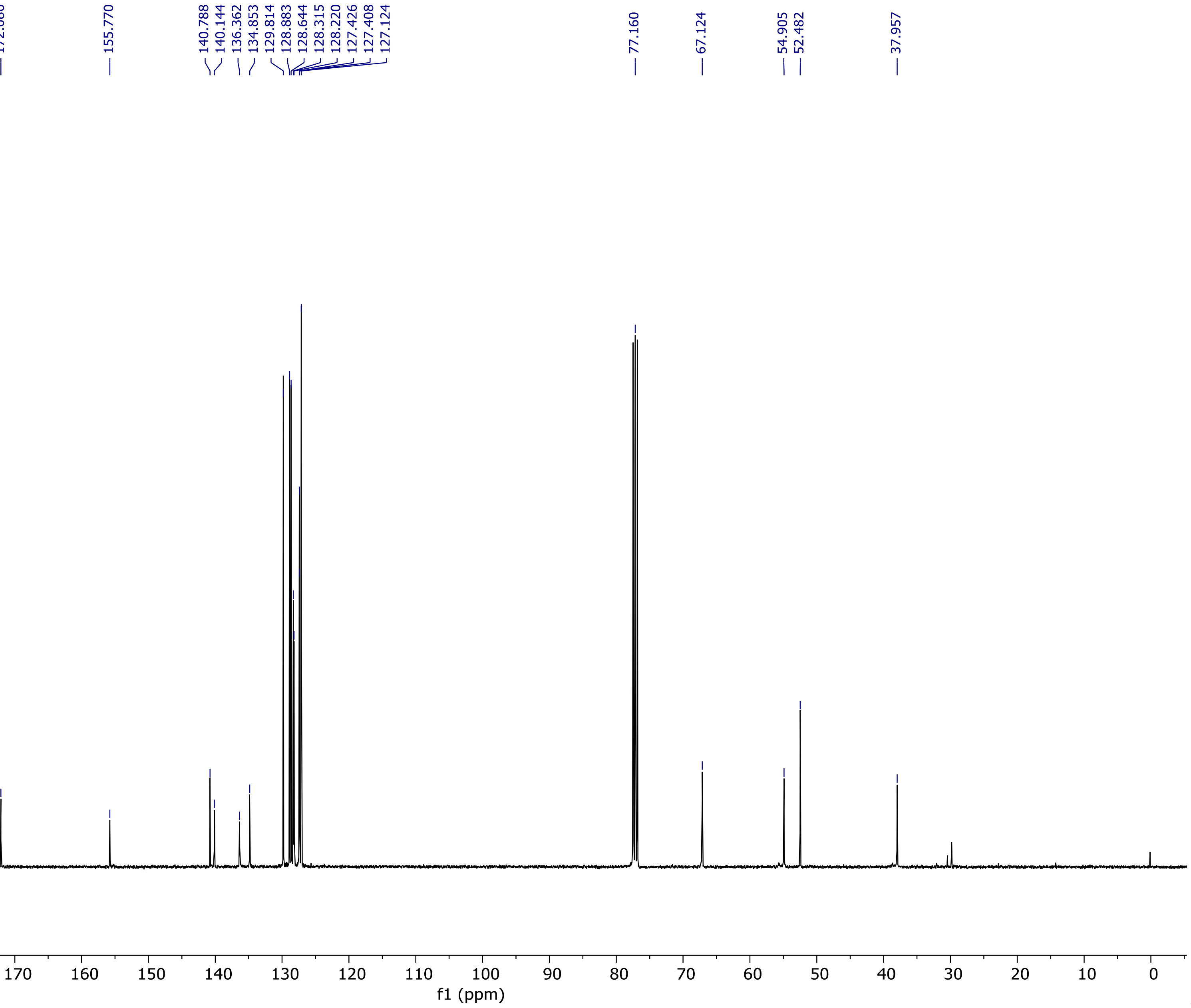

^{1}H NMR (400 MHz, $CDCl_3$)

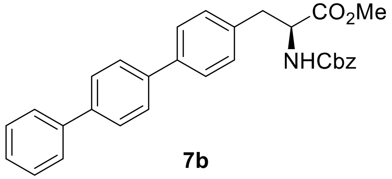

7b

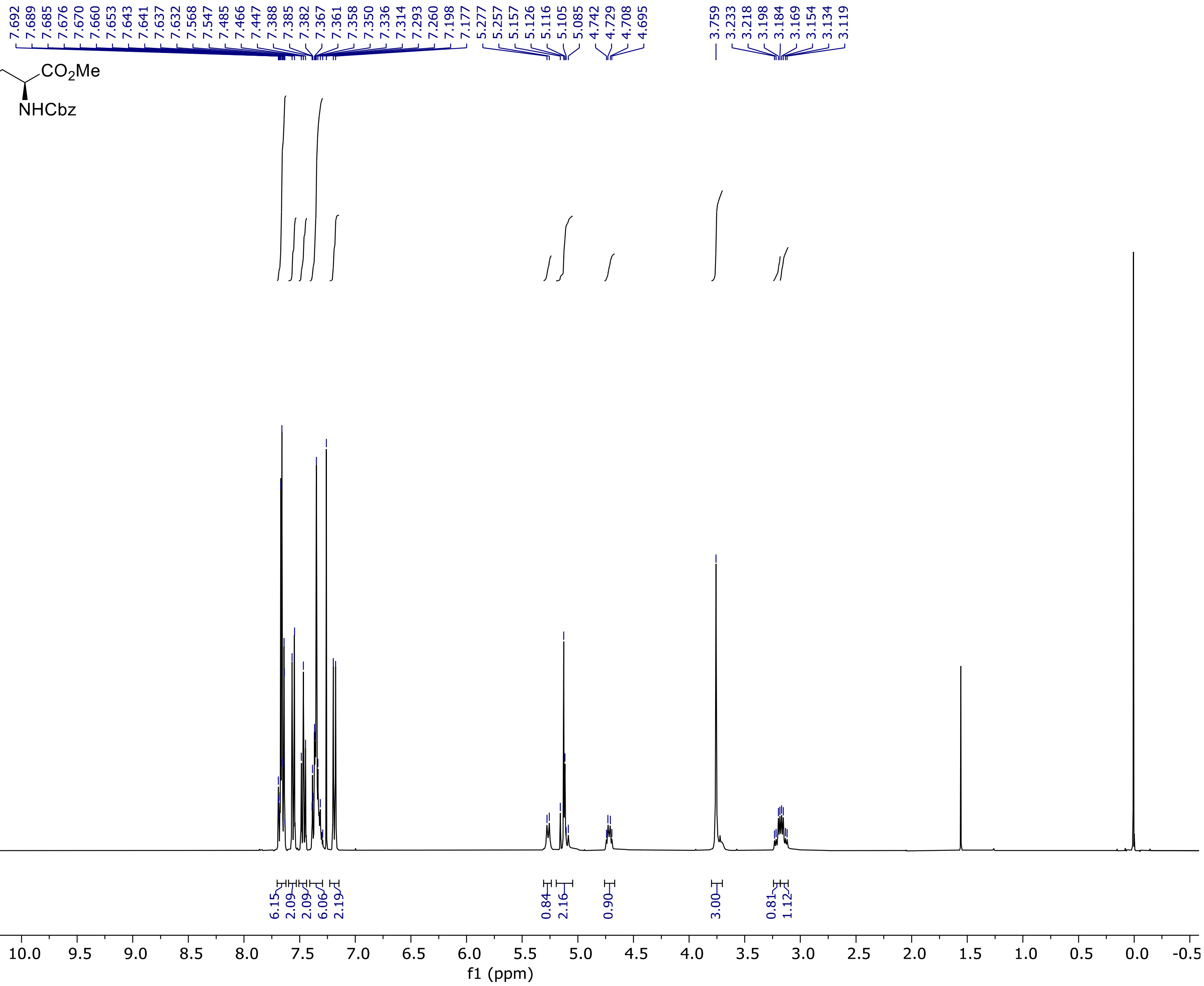

$^{13}C\{^{1}H\}$ NMR (101 MHz, $CDCl_3$)

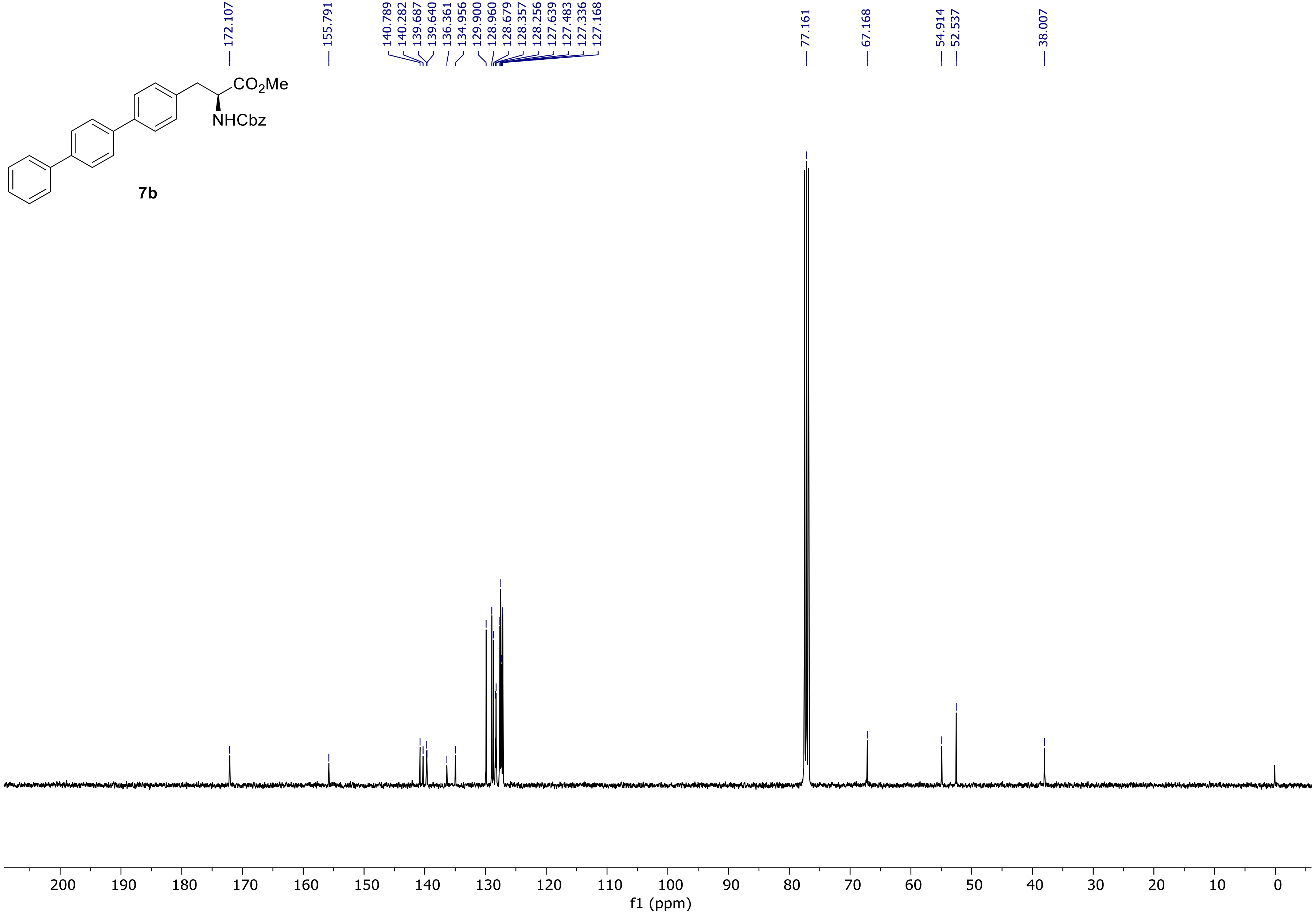

^{1}H NMR (400 MHz, $CDCl_3$)

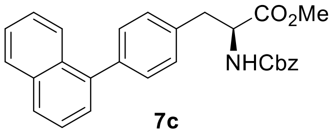

7c

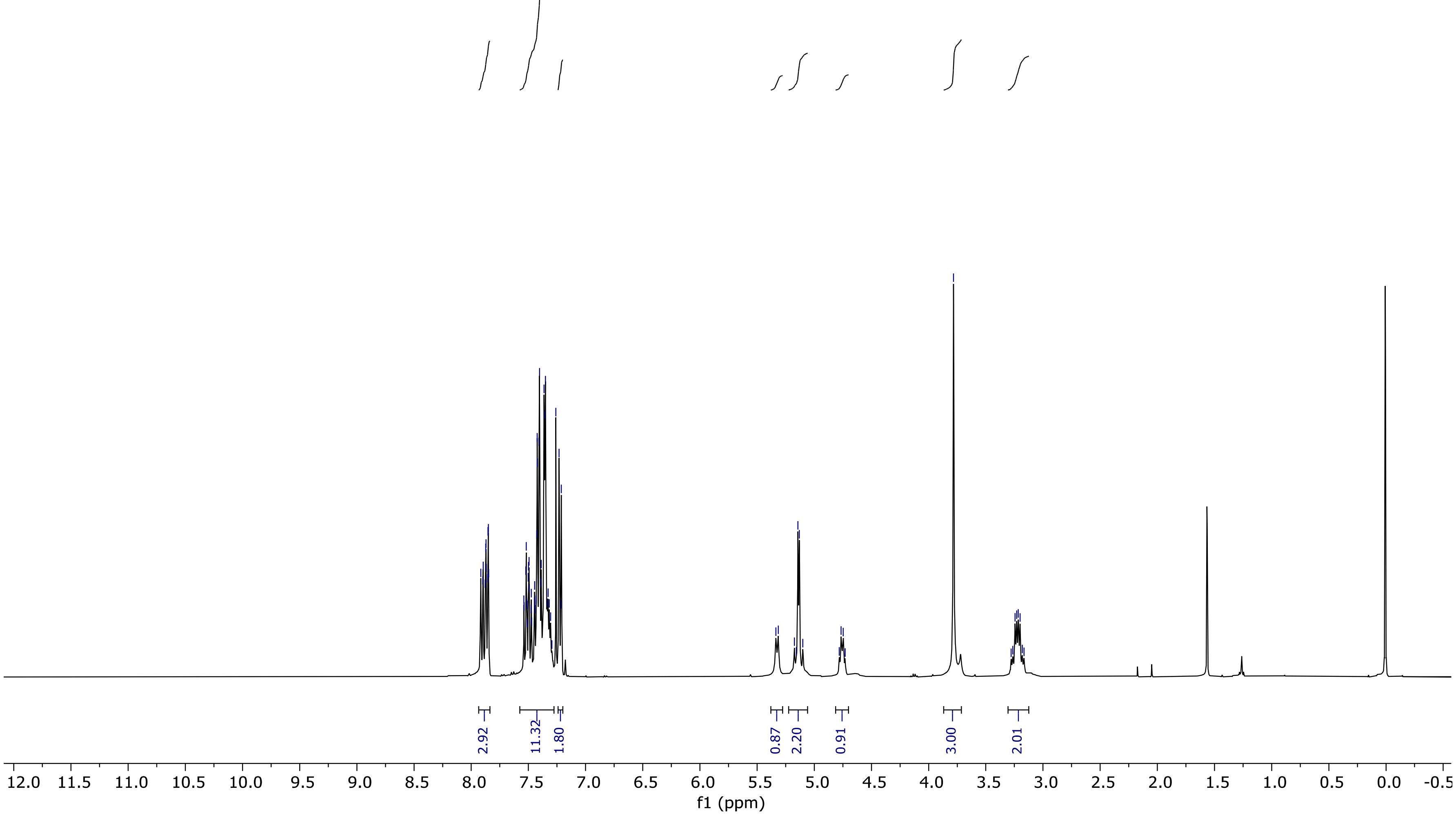

$^{13}C\{^1H\}$ NMR (101 MHz, $CDCl_3$)

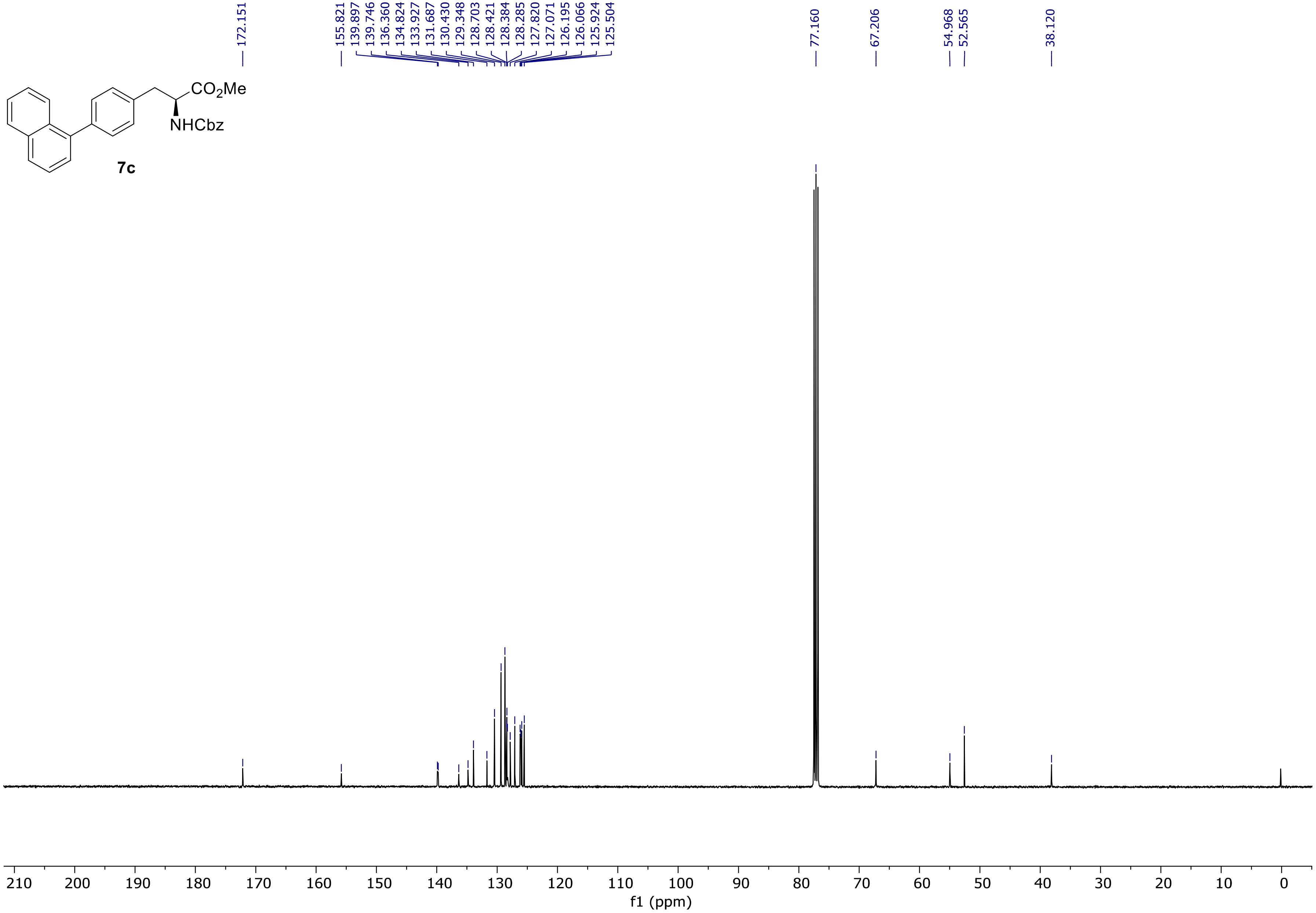

^1H NMR (400 MHz, $CDCl_3$)

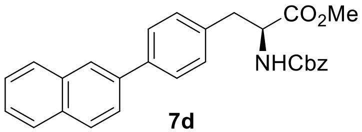

7d

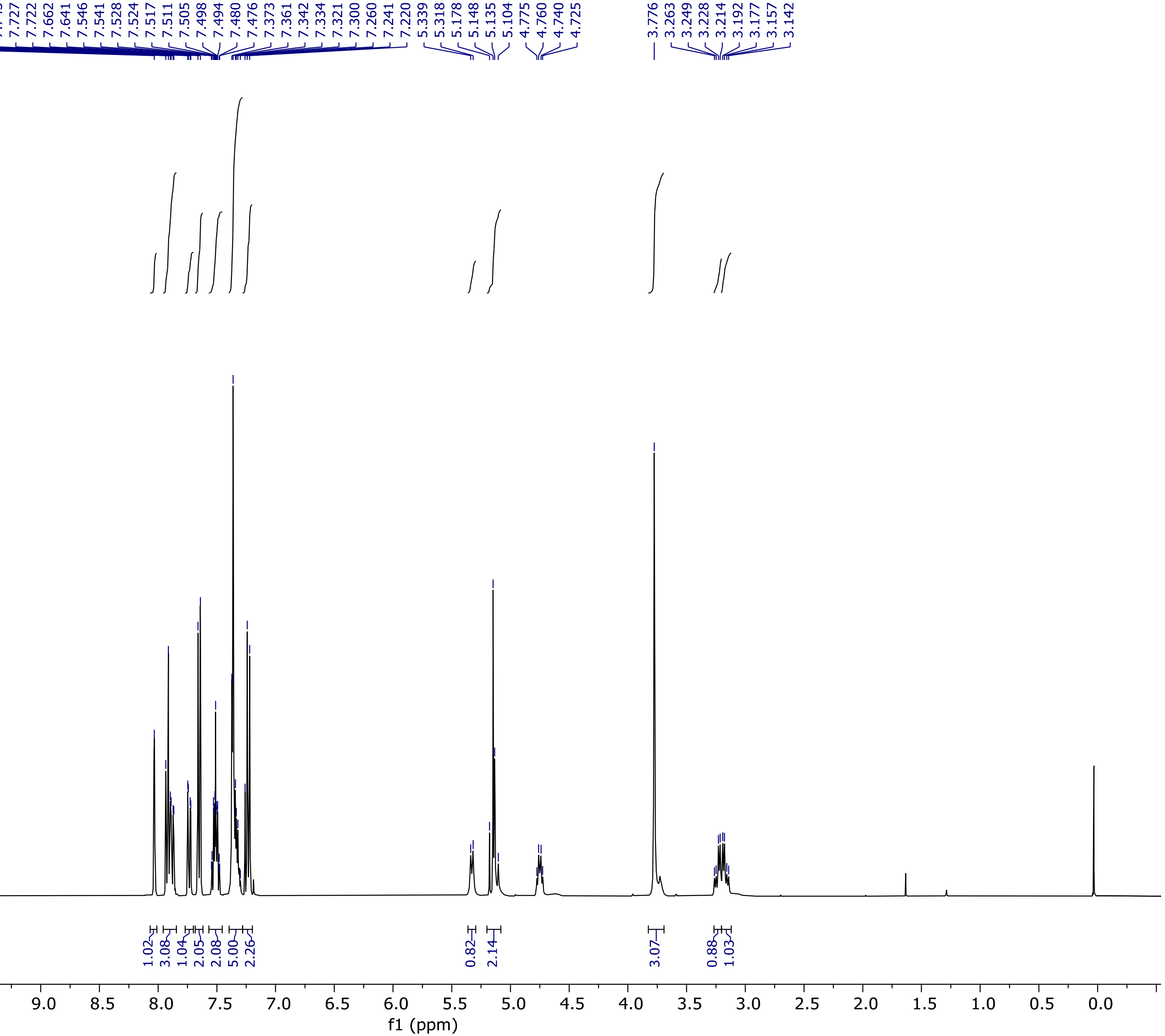

$^{13}C\{^{1}H\}$ NMR (101 MHz, $CDCl_3$)

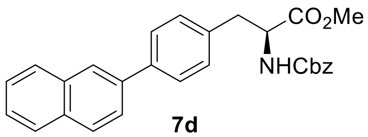

7d

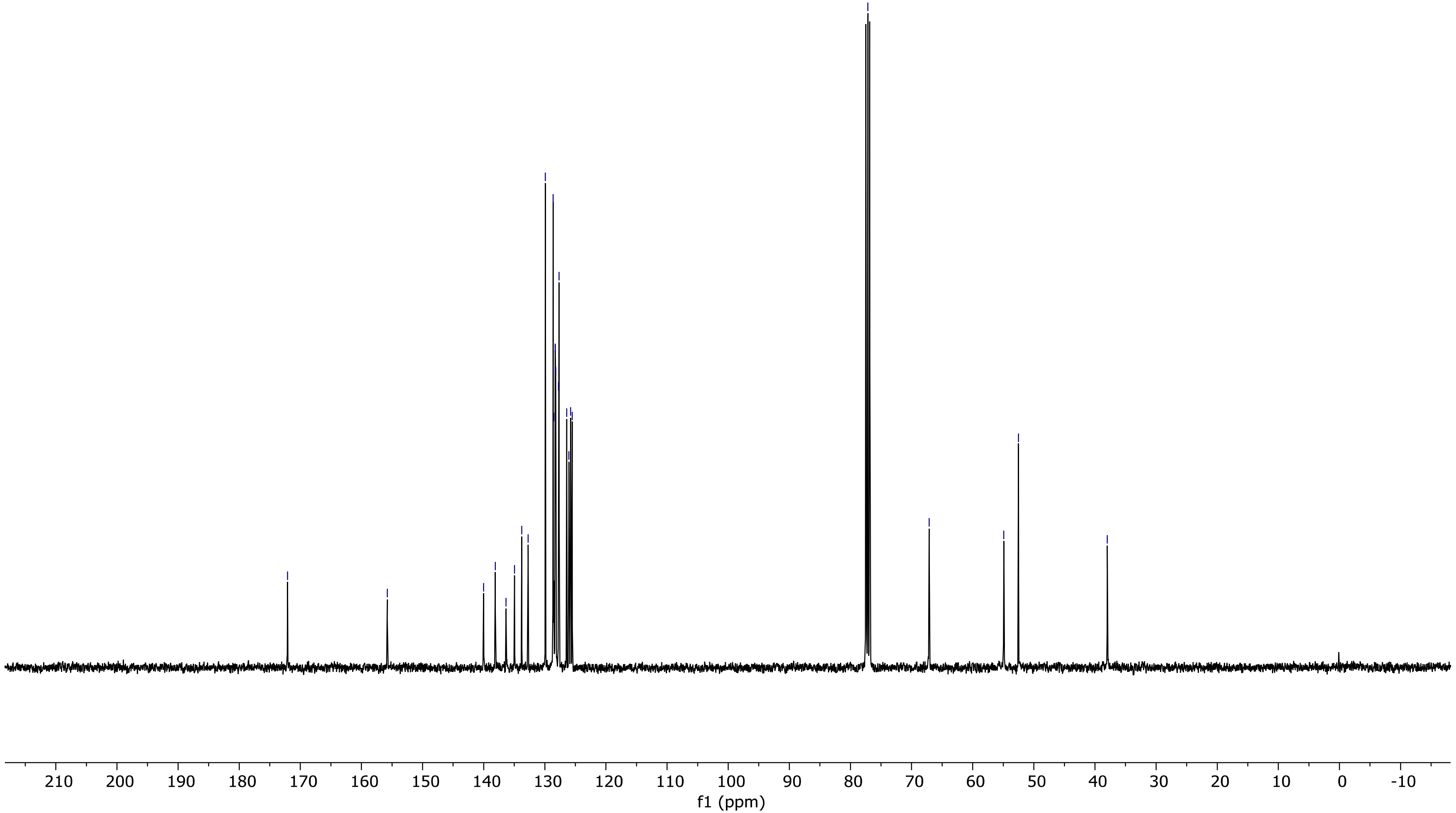

^{1}H NMR (400 MHz, $CDCl_3$)

7e

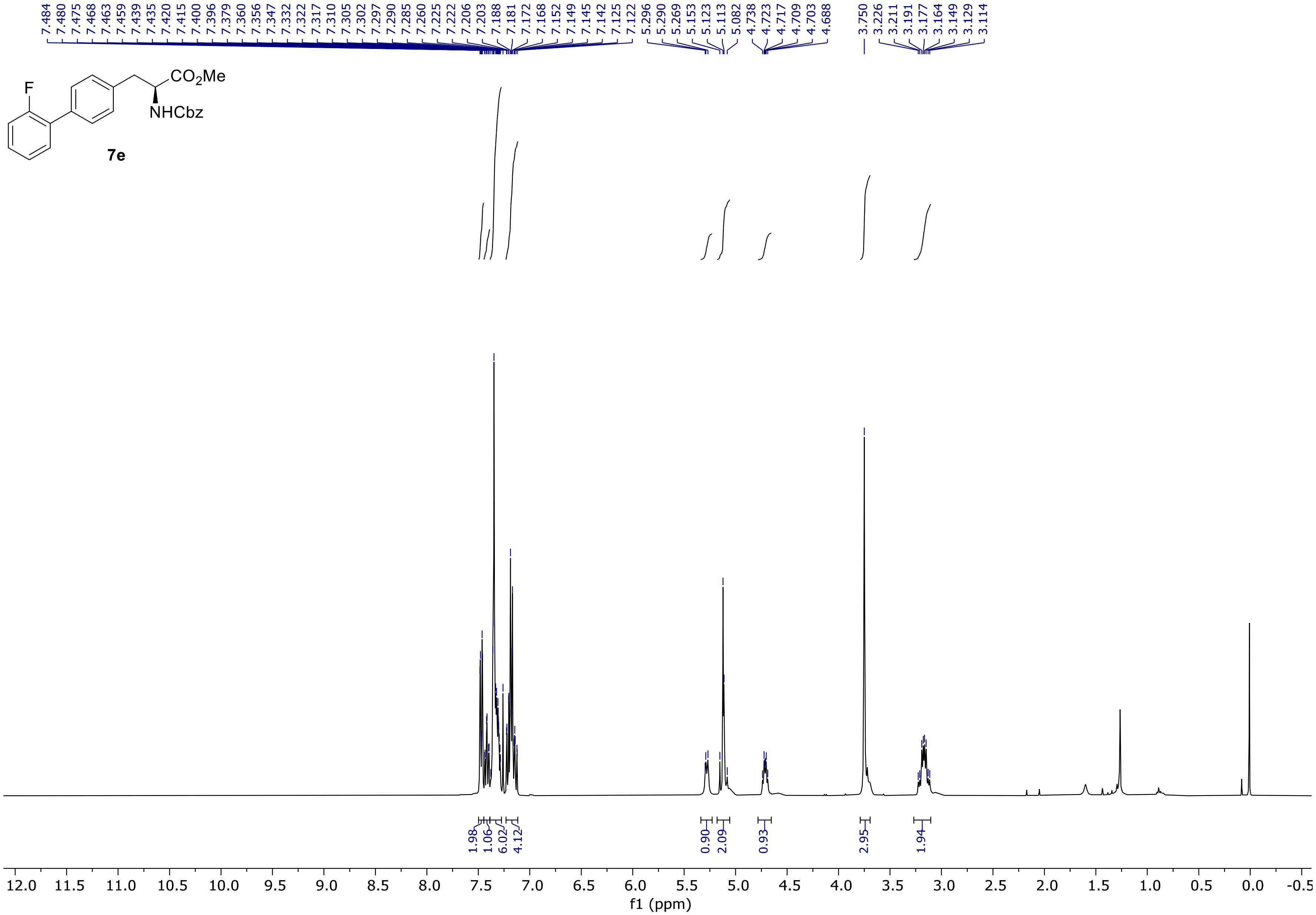

$^{13}C\{^{1}H\}$ NMR (101 MHz, $CDCl_3$)

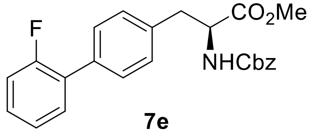

7e

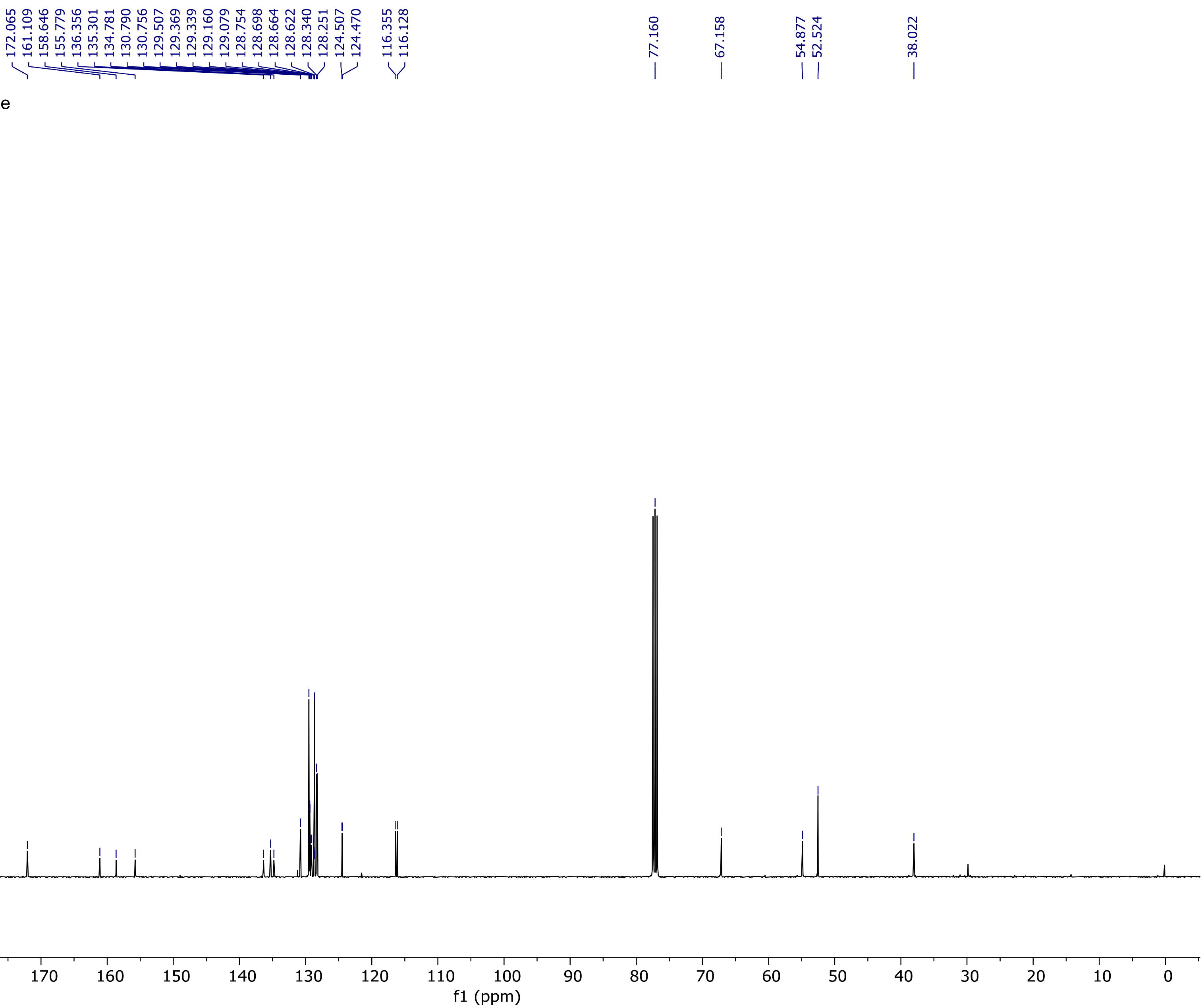

^{1}H NMR (400 MHz, $CDCl_3$)

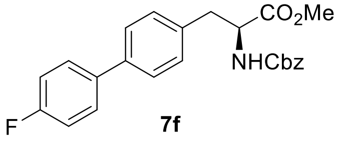

7f

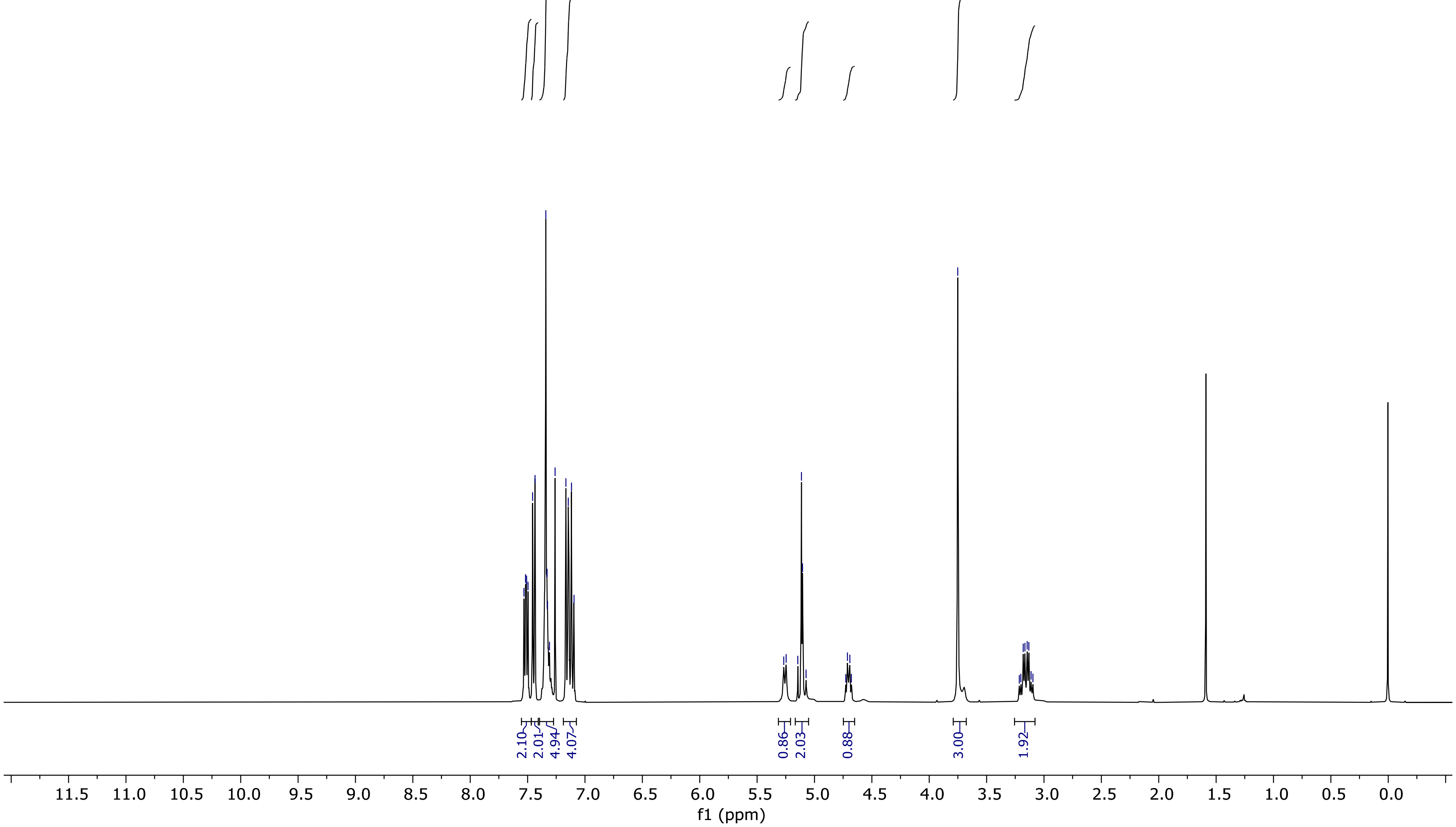

$^{13}C\{^1H\}$ NMR (101 MHz, $CDCl_3$)

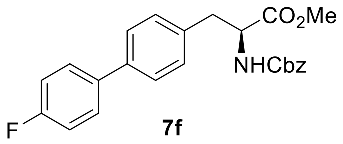

7f

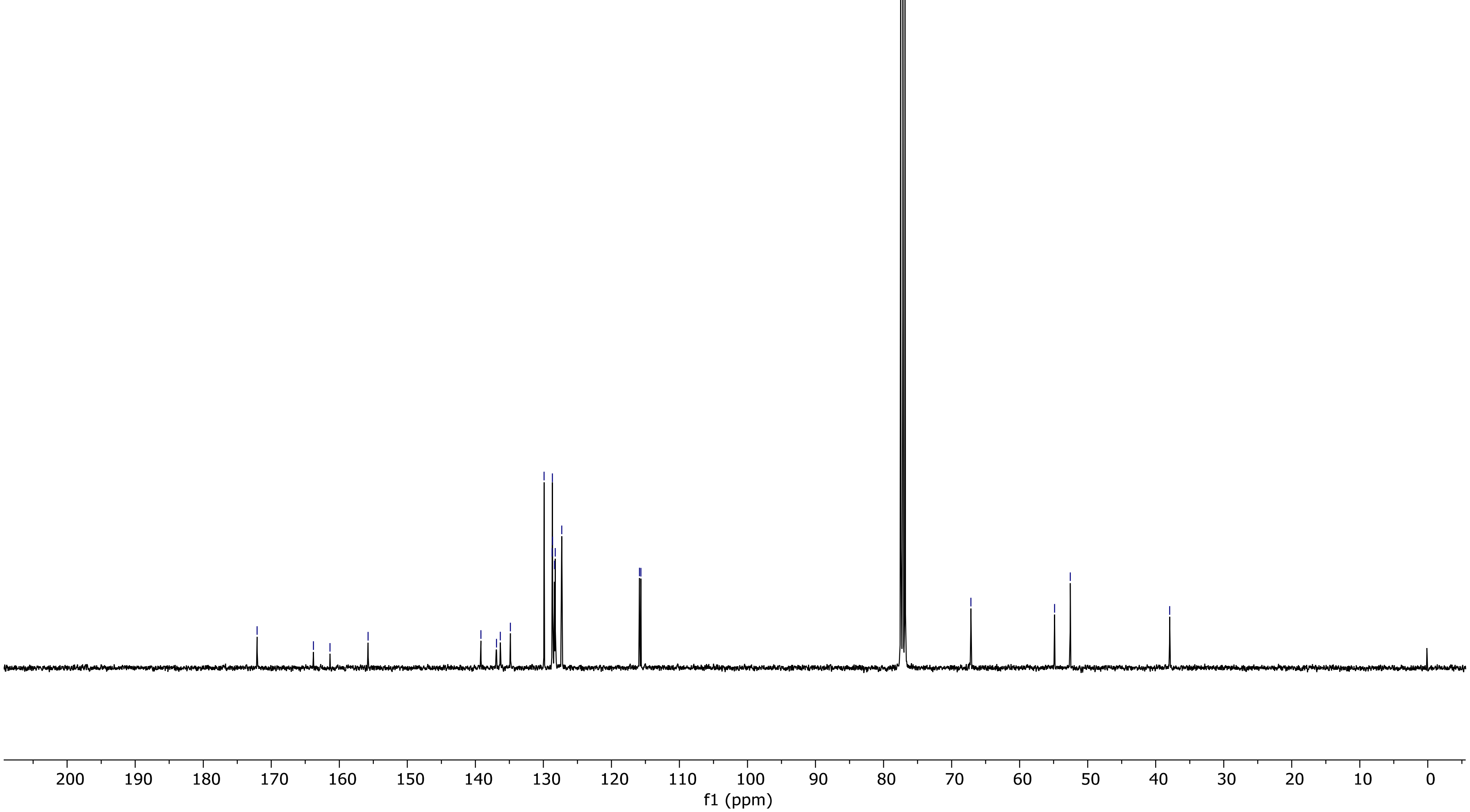

^{1}H NMR (400 MHz, $CDCl_3$)

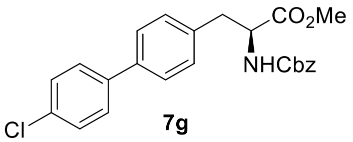

7g

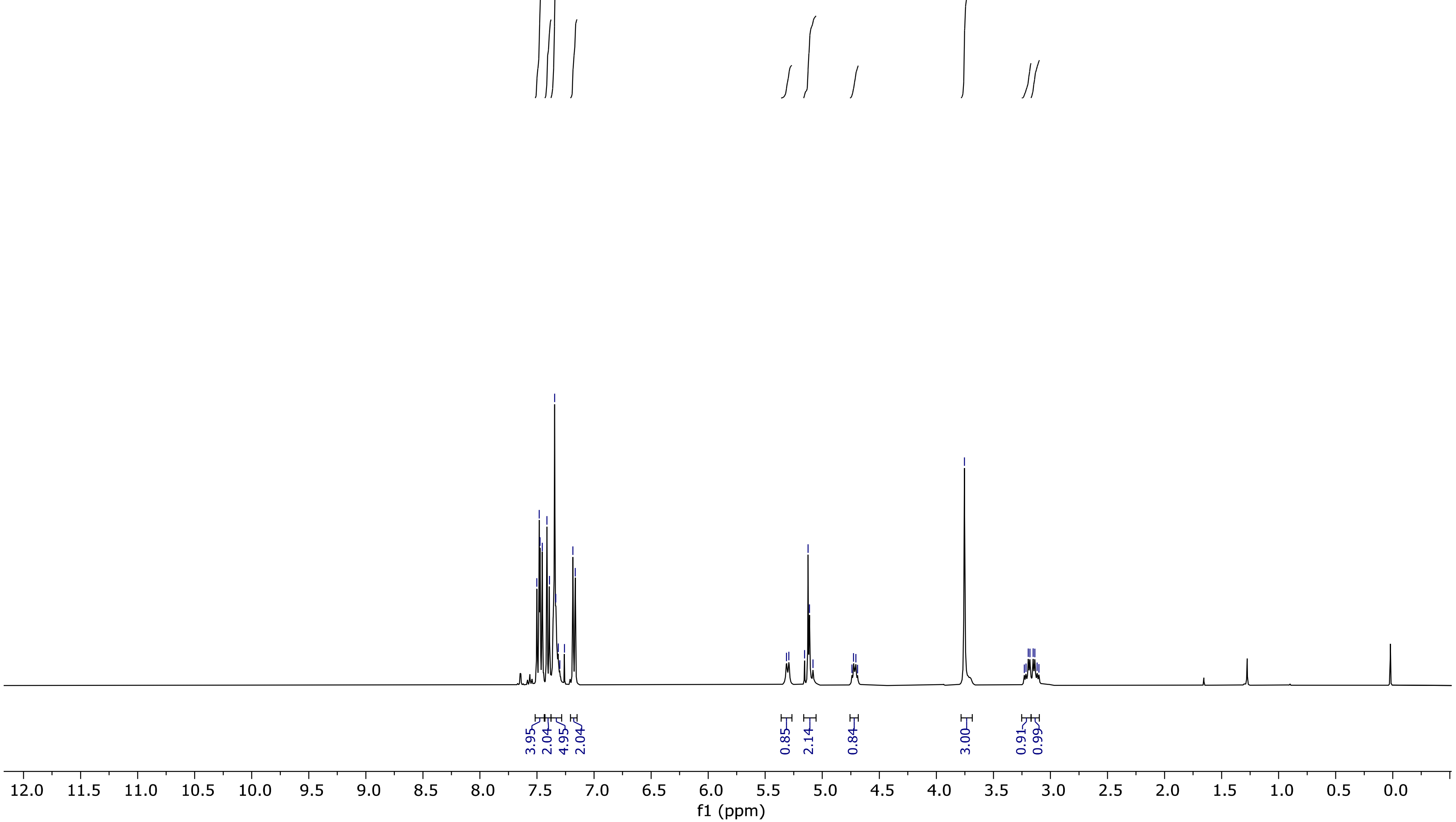

$^{13}C\{^{1}H\}$ NMR (101 MHz, CDCl$_3$)

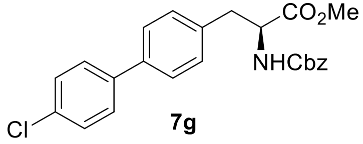

7g

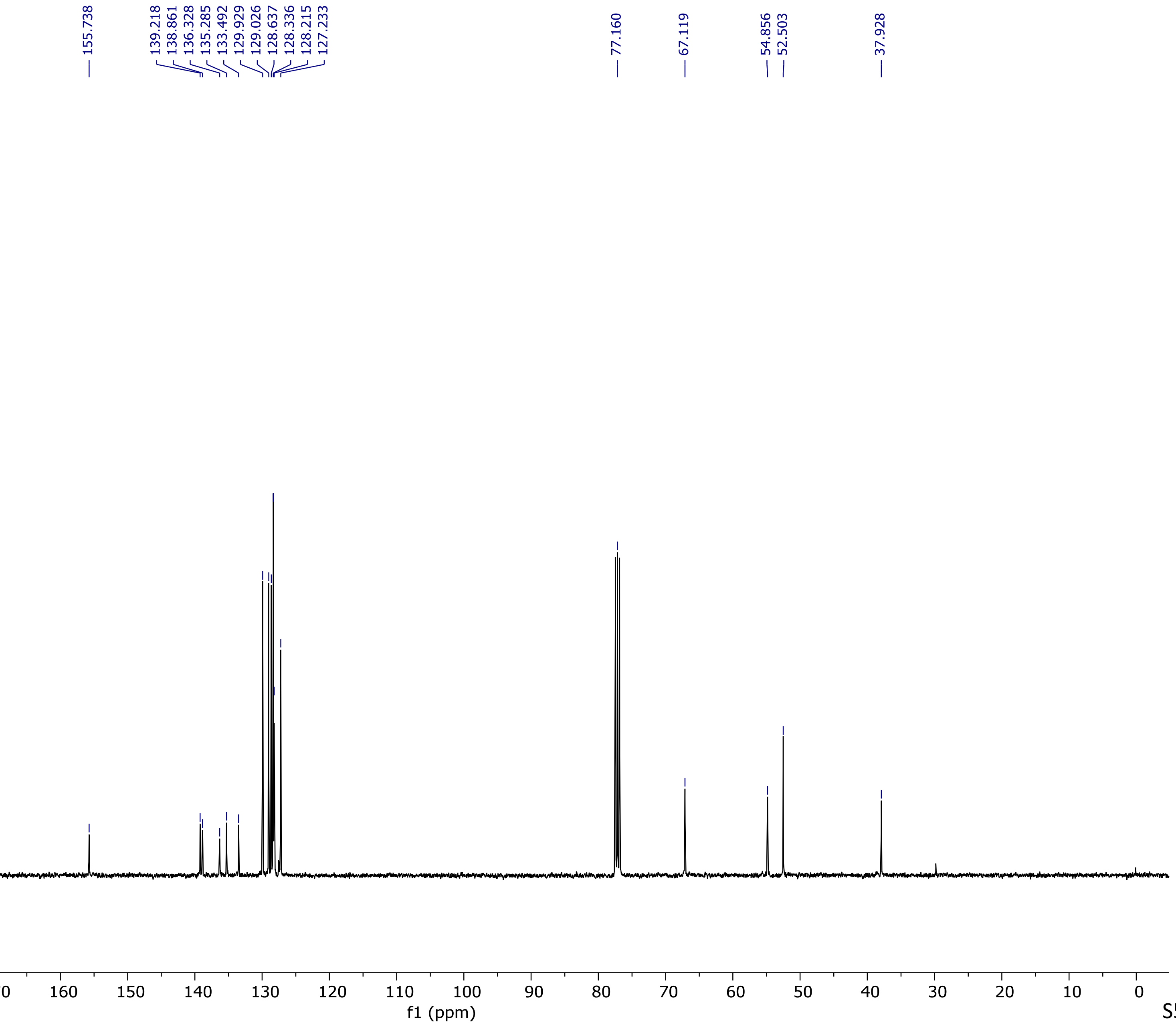

^{1}H NMR (400 MHz, $CDCl_3$)

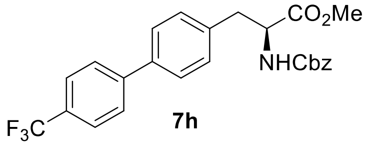

7h

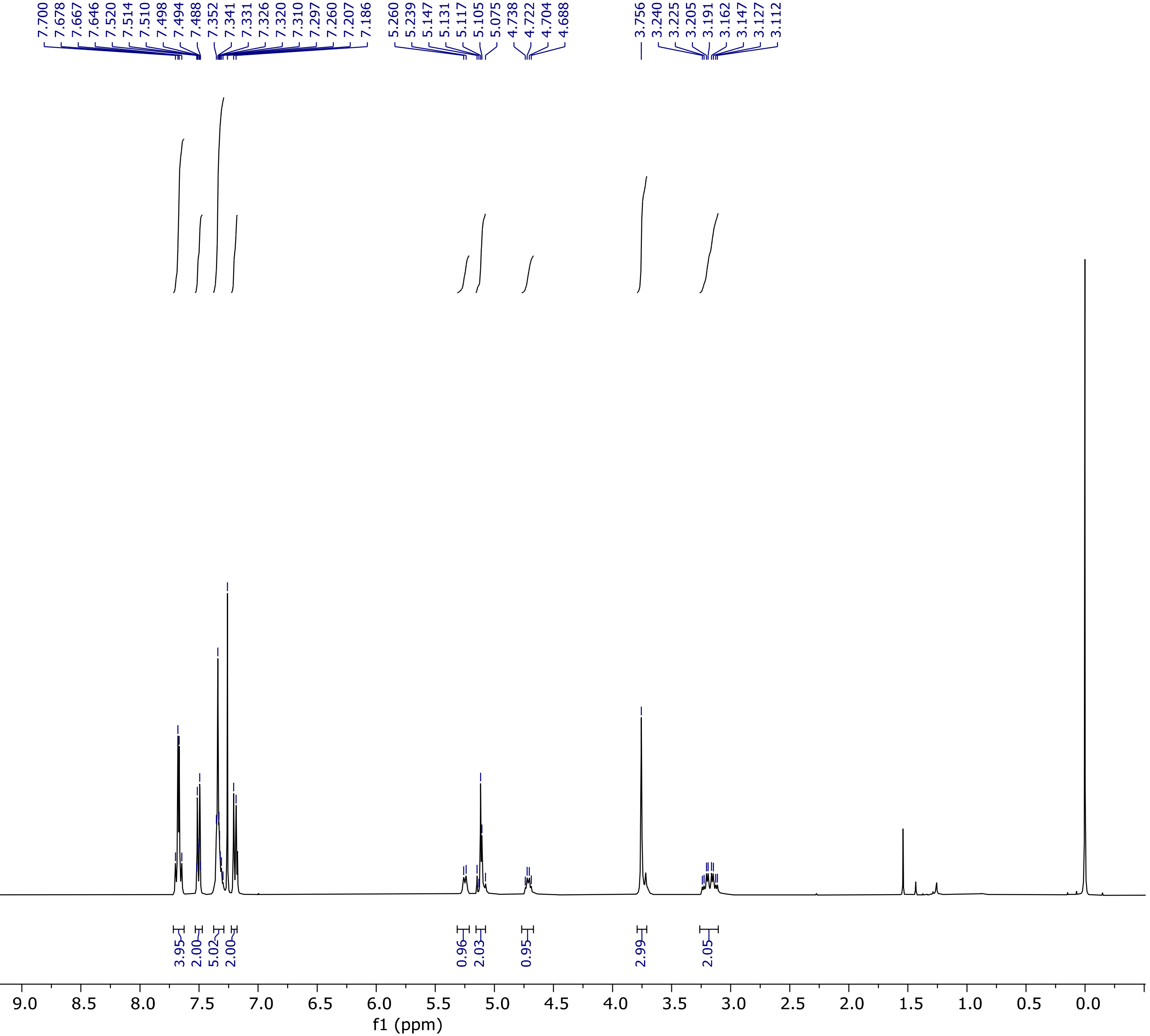

$^{13}C\{^{1}H\}$ NMR (101 MHz, $CDCl_3$)

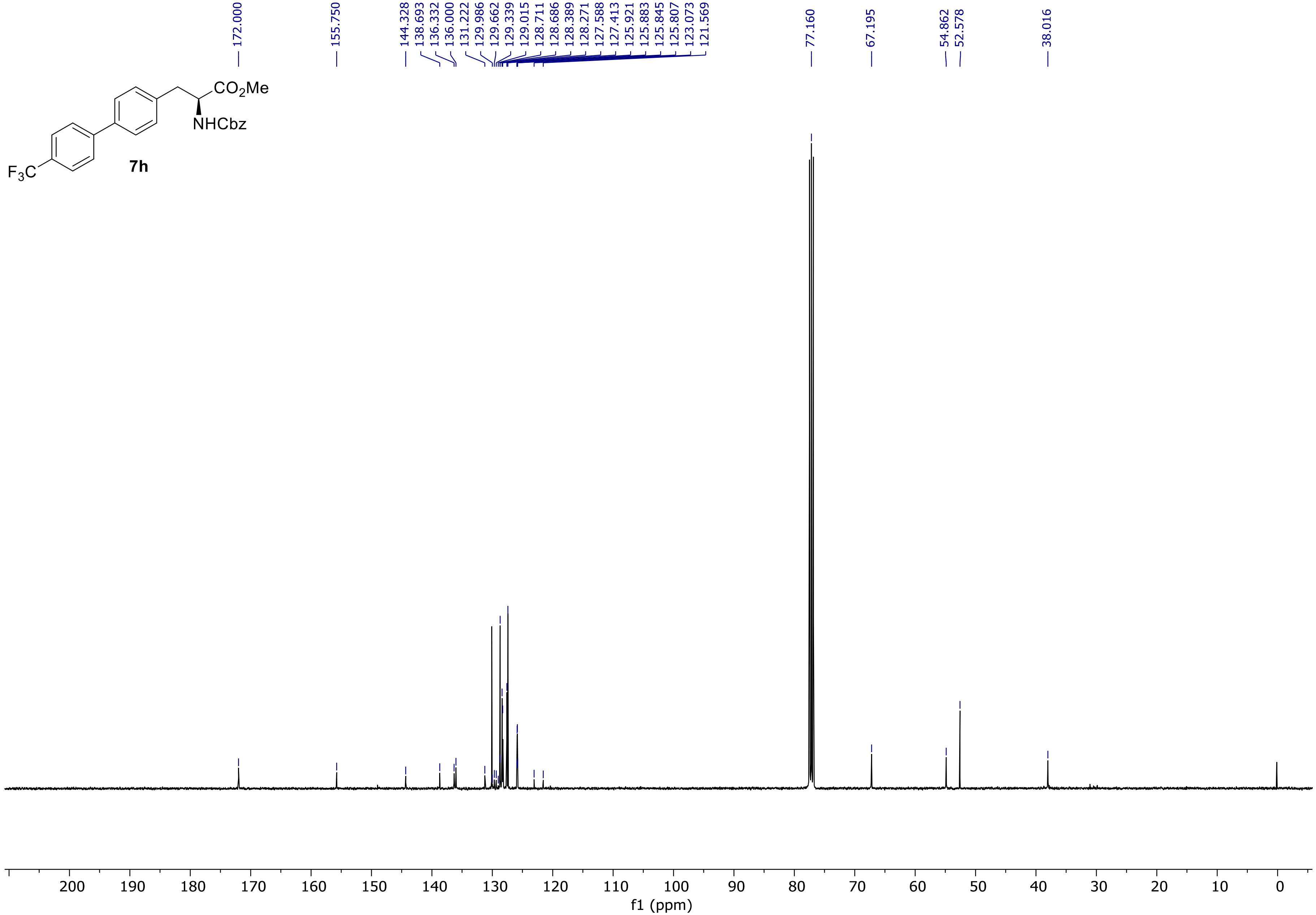

^{1}H NMR (400 MHz, $CDCl_3$)

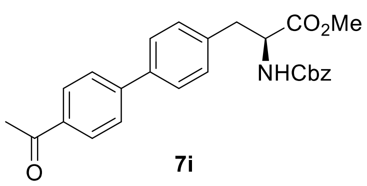

7i

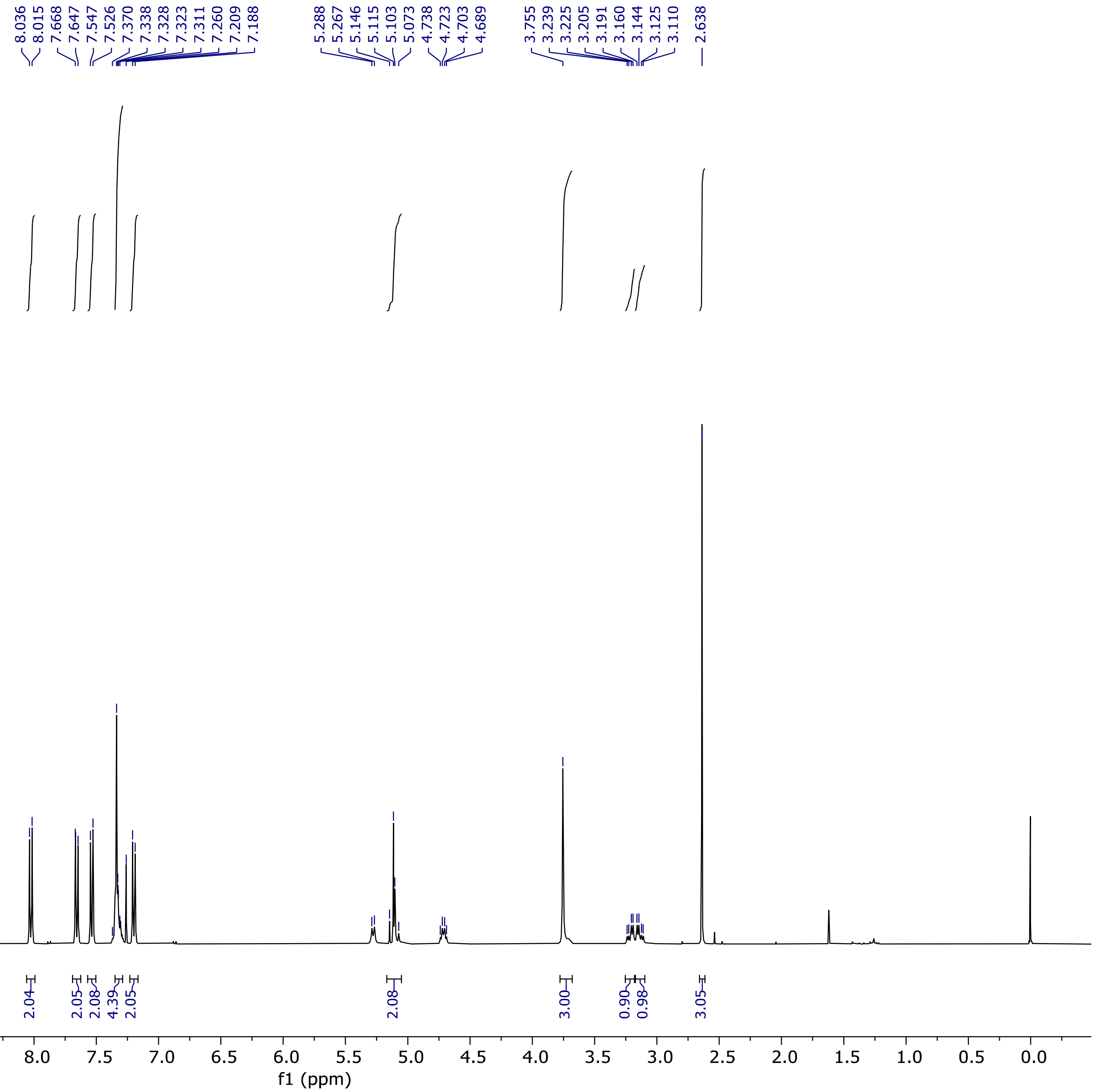

$^{13}C\{^1H\}$ NMR (101 MHz, $CDCl_3$)

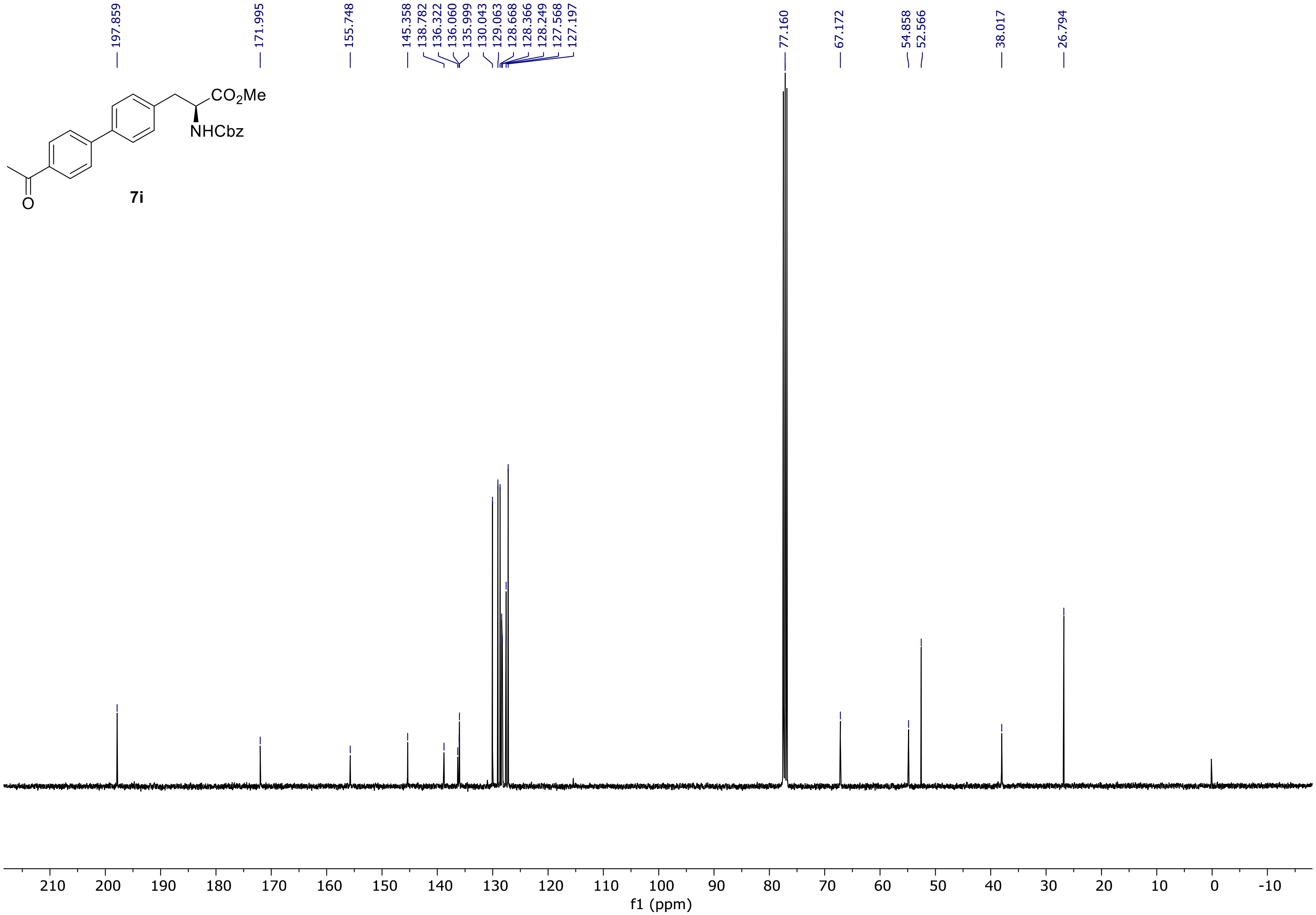

^{1}H NMR (400 MHz, $CDCl_3$)

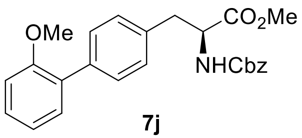

7j

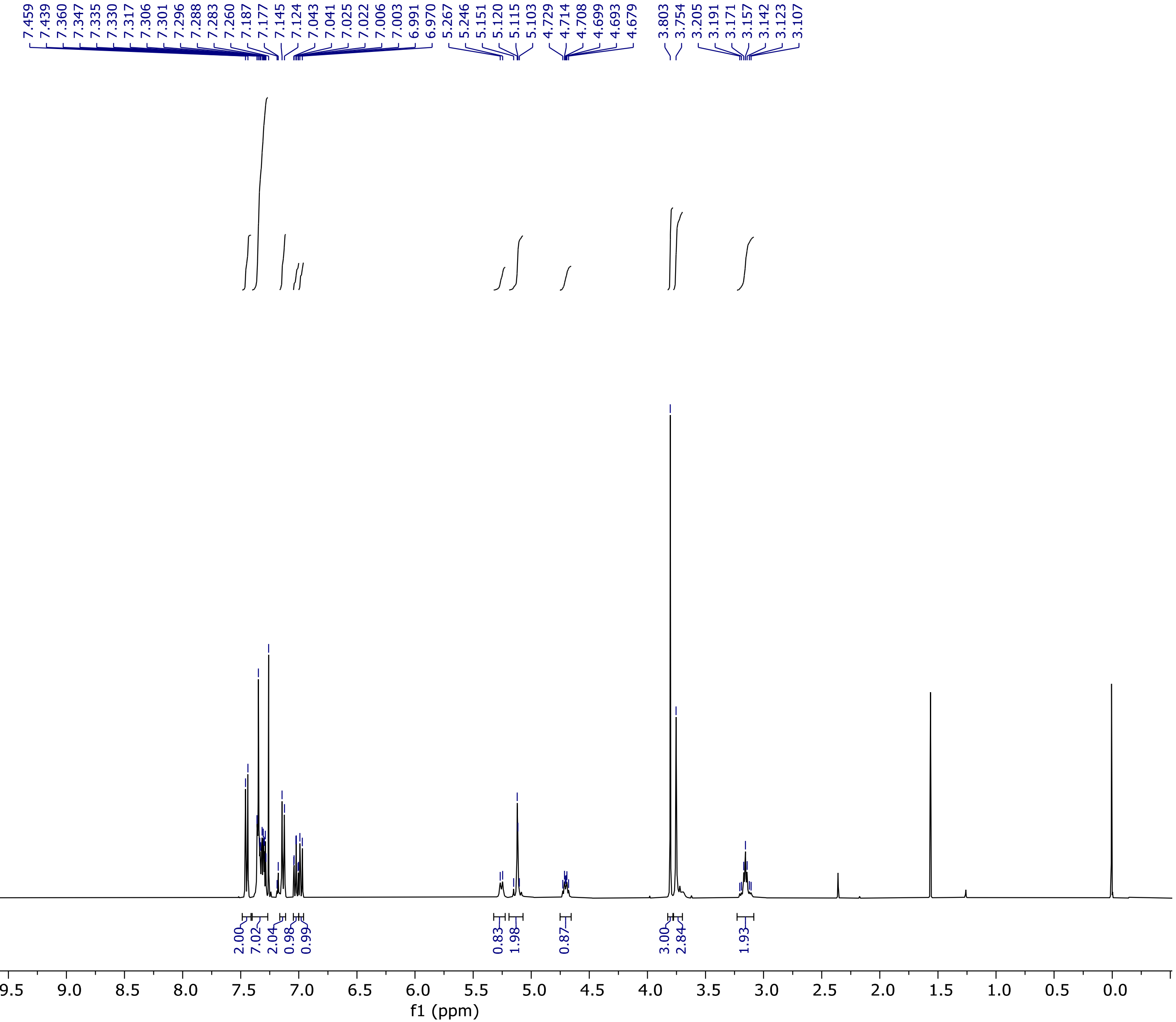

$^{13}C\{^{1}H\}$ NMR (101 MHz, $CDCl_3$)

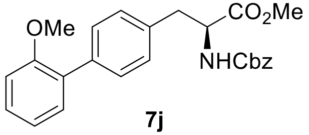

7j

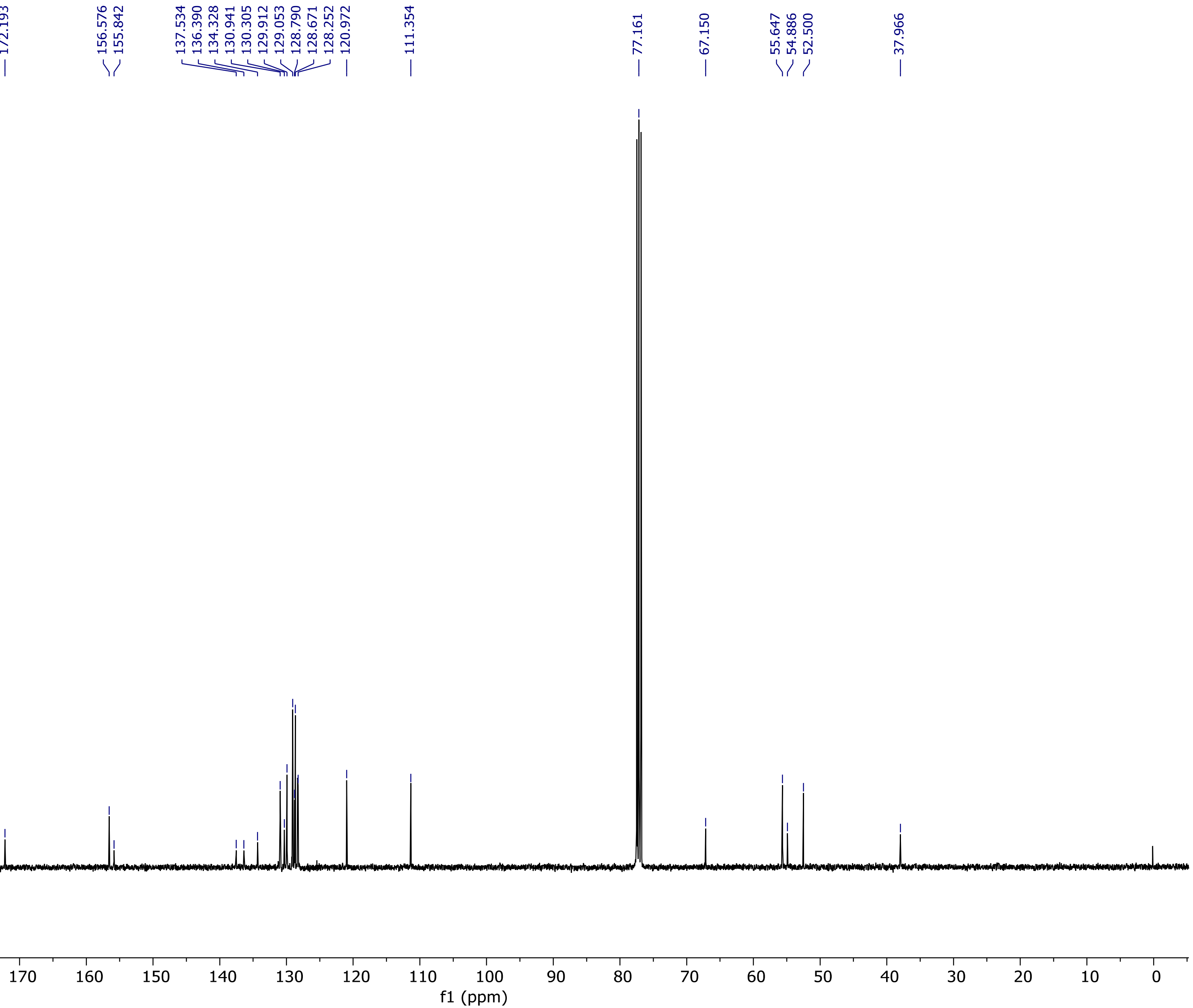

^{1}H NMR (400 MHz, $CDCl_3$)

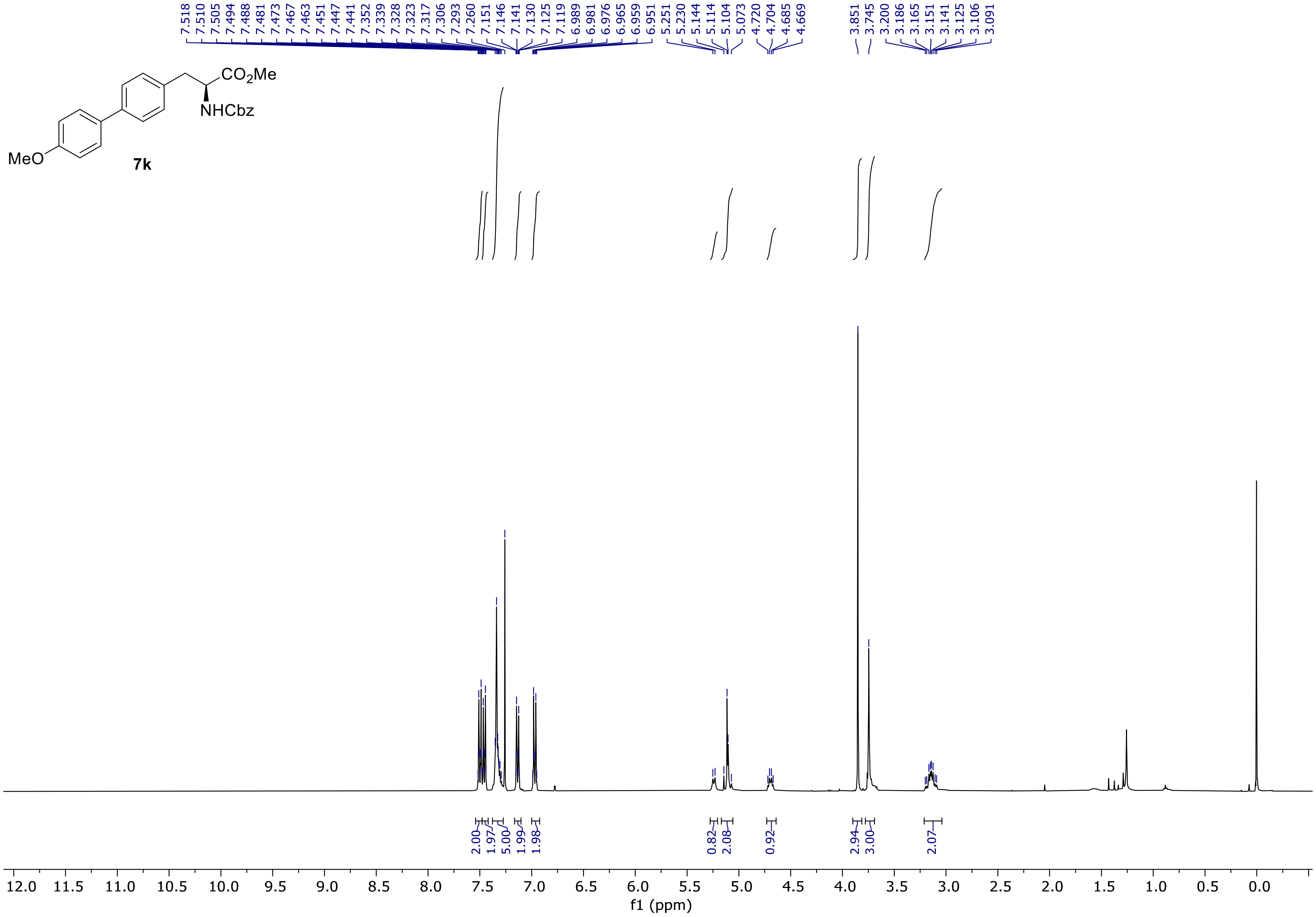

$^{13}C\{^{1}H\}$ NMR (101 MHz, $CDCl_3$)

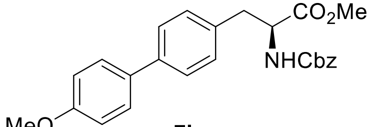

7k

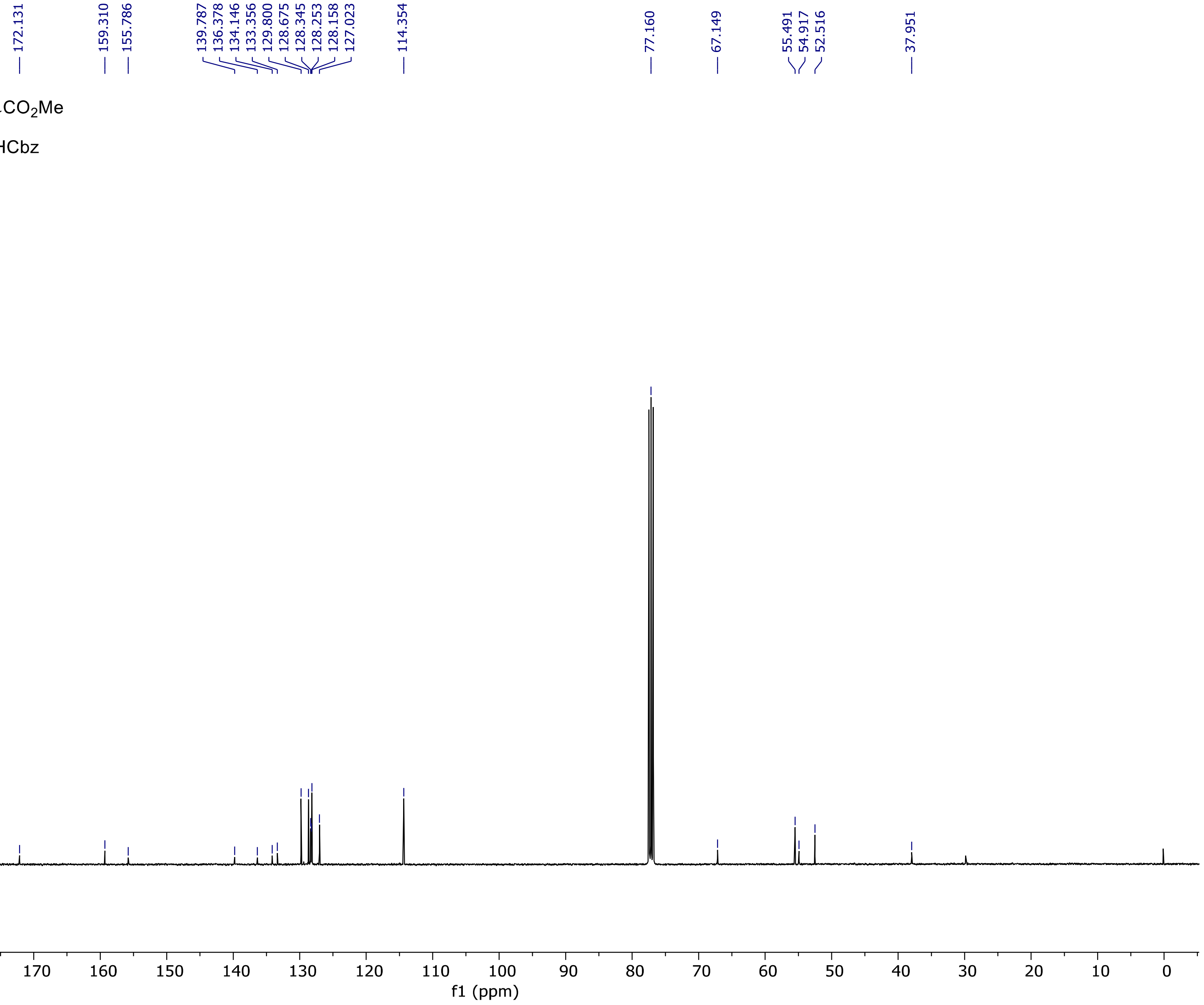

^{1}H NMR (400 MHz, $CDCl_3$)

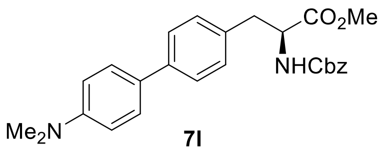

7l

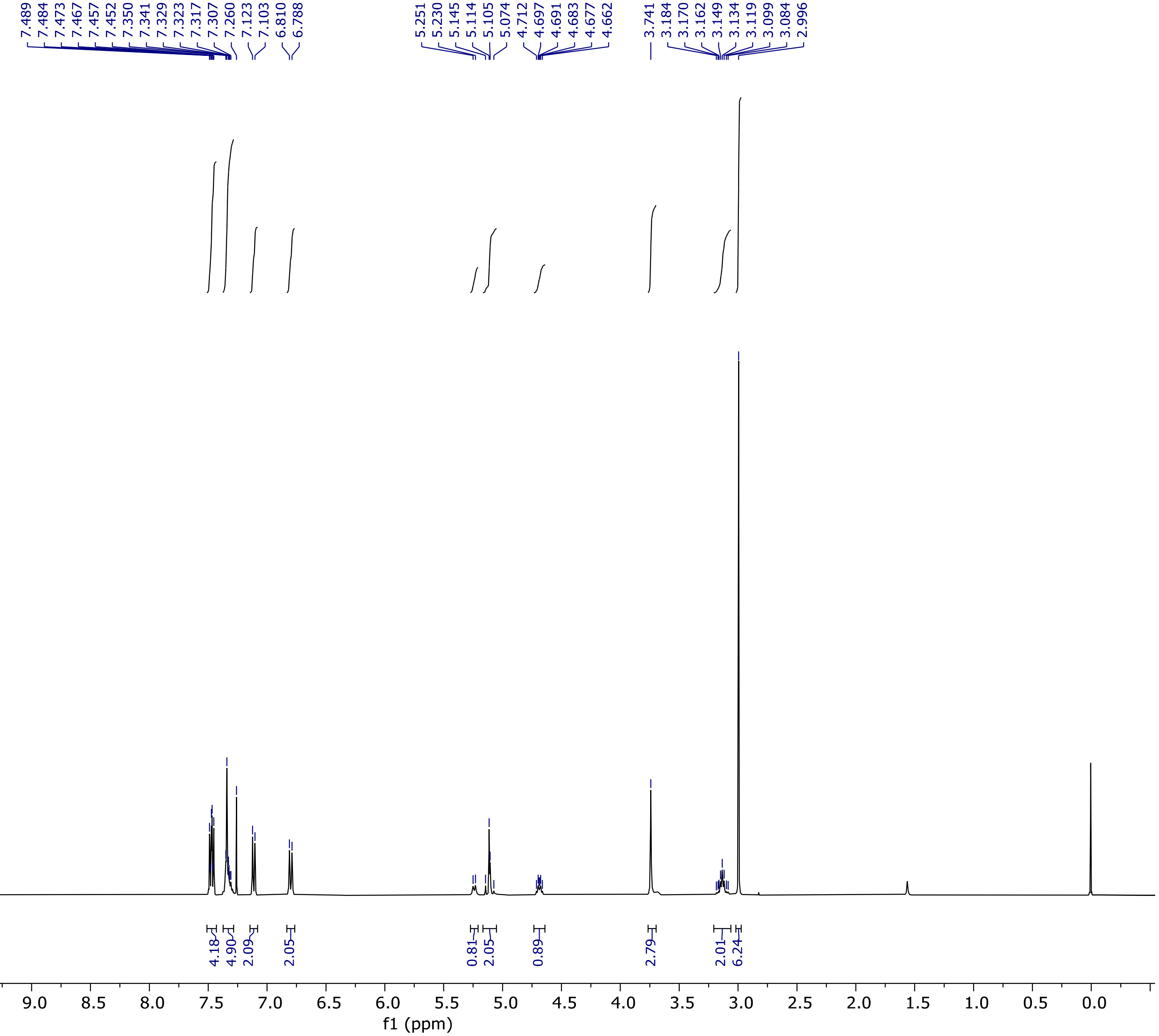

$^{13}C\{^1H\}$ NMR (101 MHz, CDCl$_3$)

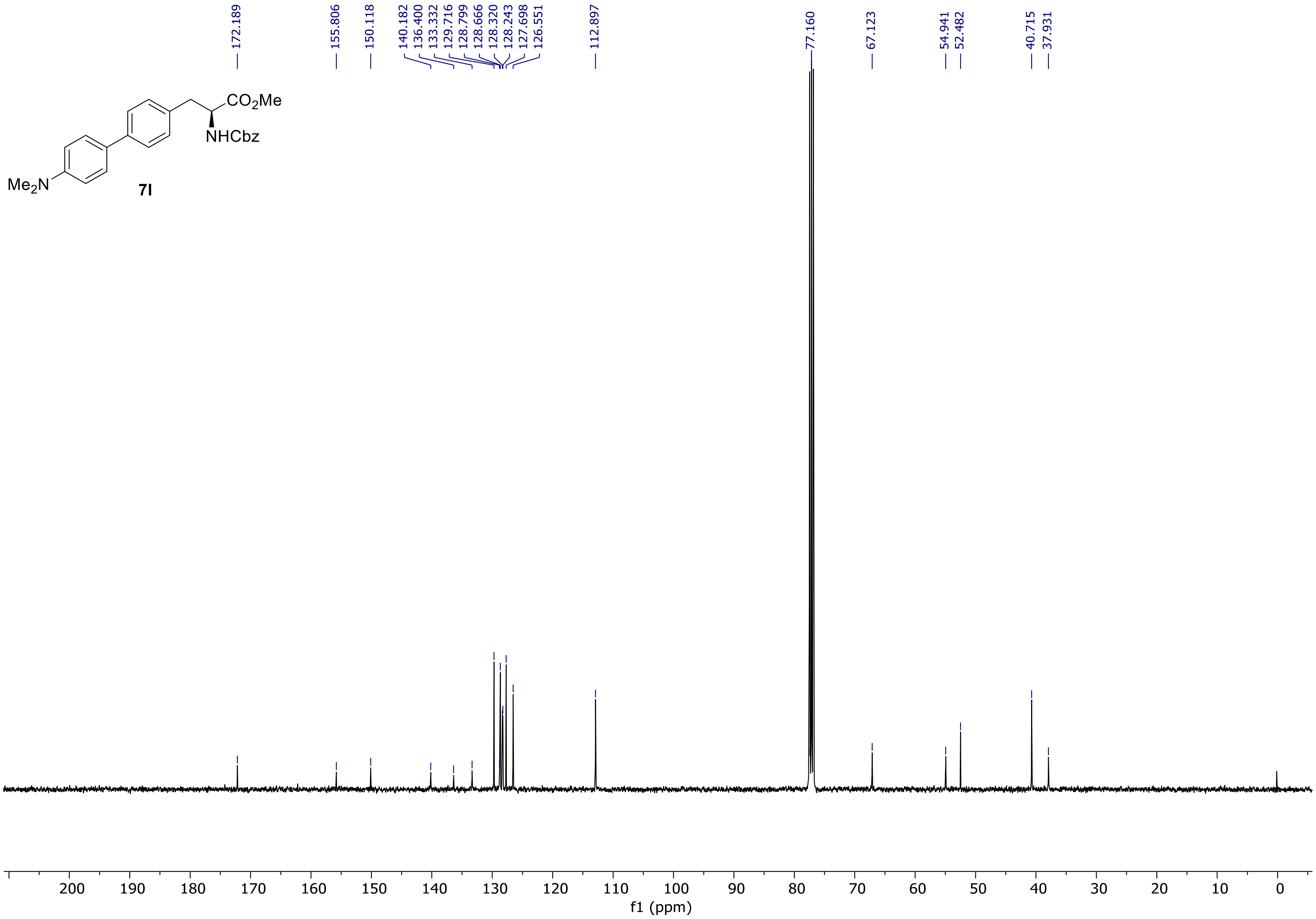

^{1}H NMR (400 MHz, $CDCl_3$)

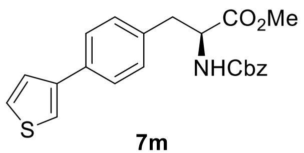

7m

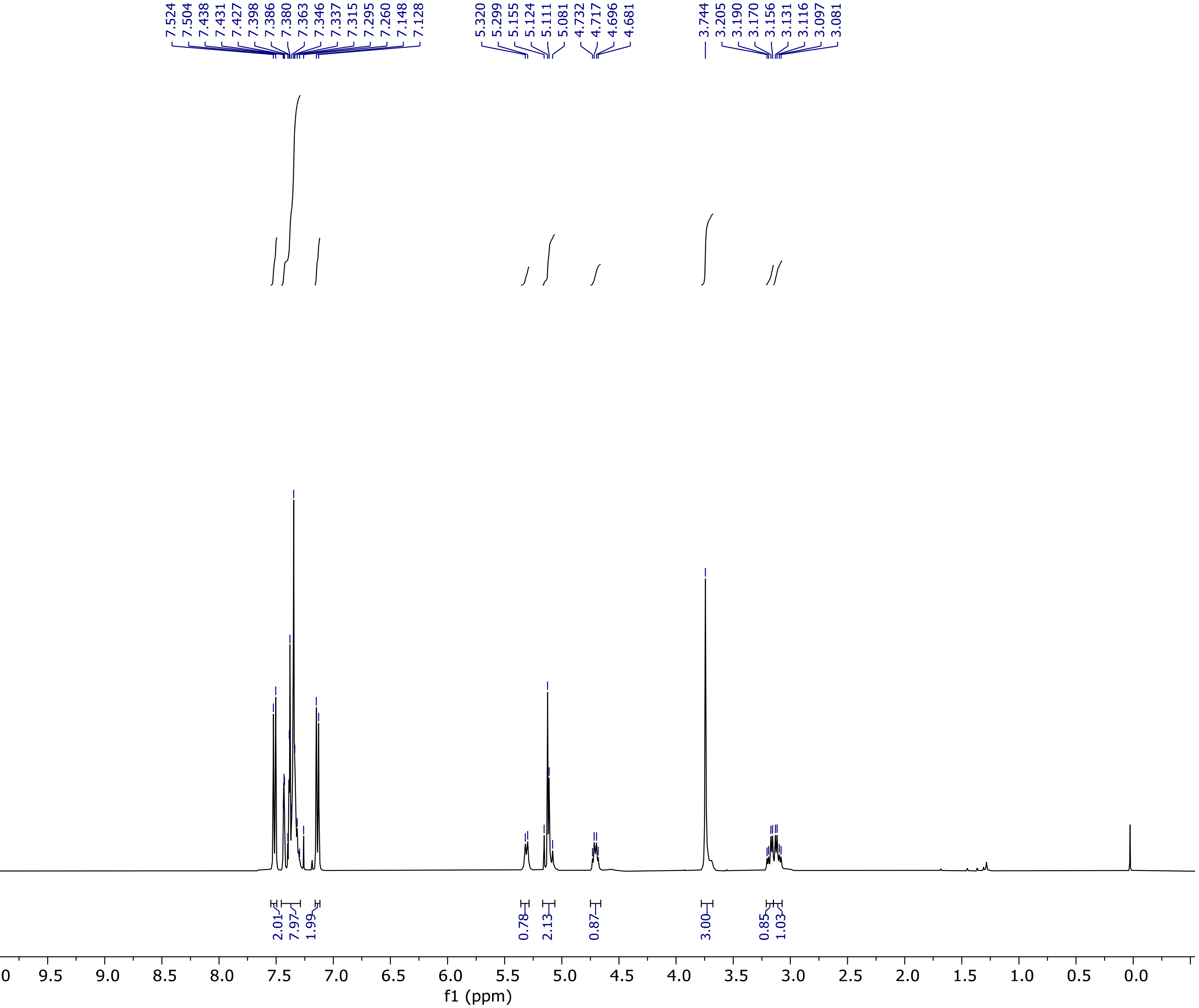

$^{13}C\{^{1}H\}$ NMR (101 MHz, $CDCl_3$)

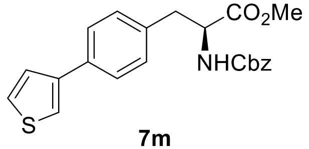

7m

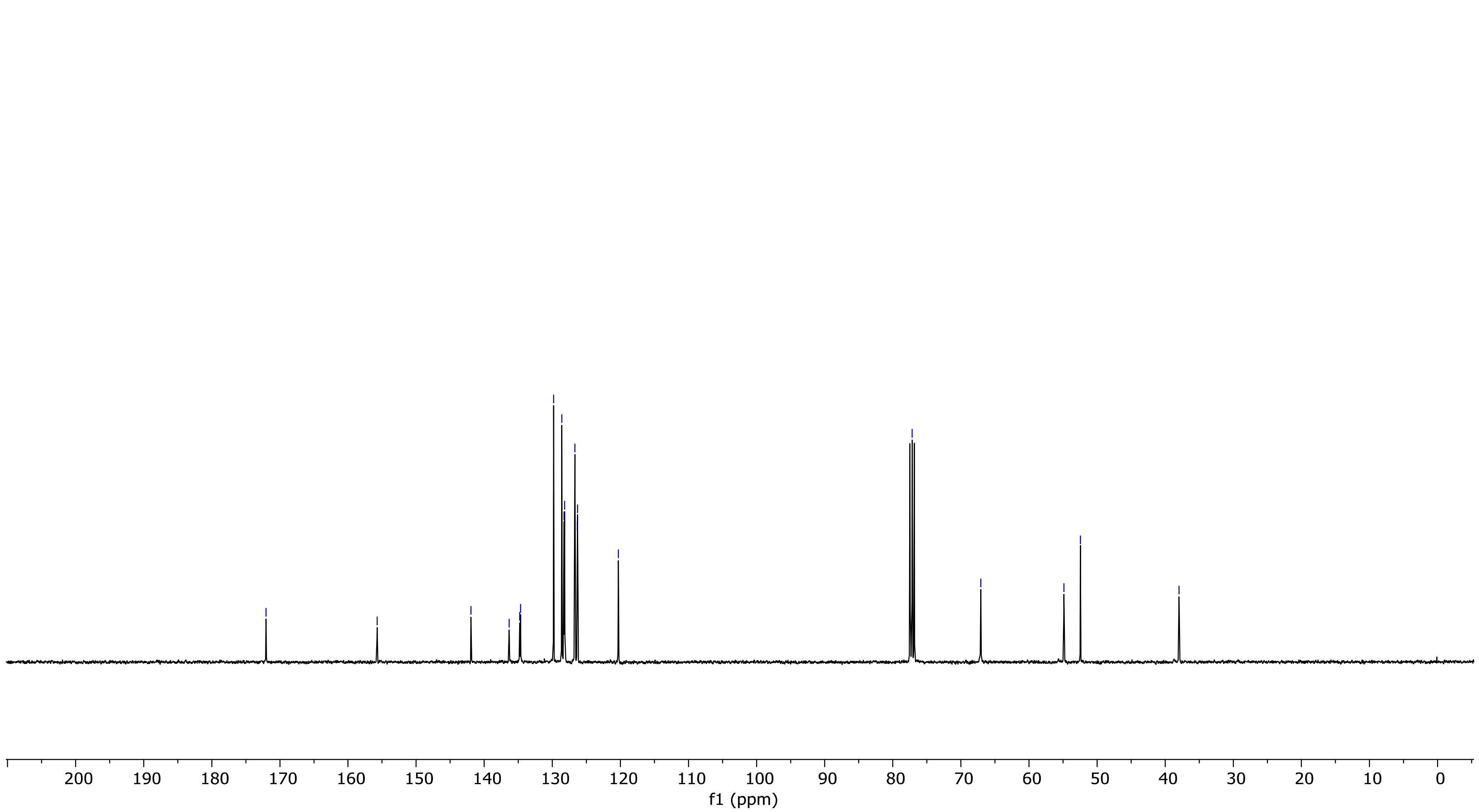

^1H NMR (400 MHz, CD_3OD)

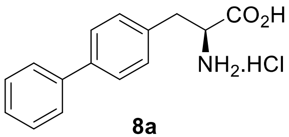

8a

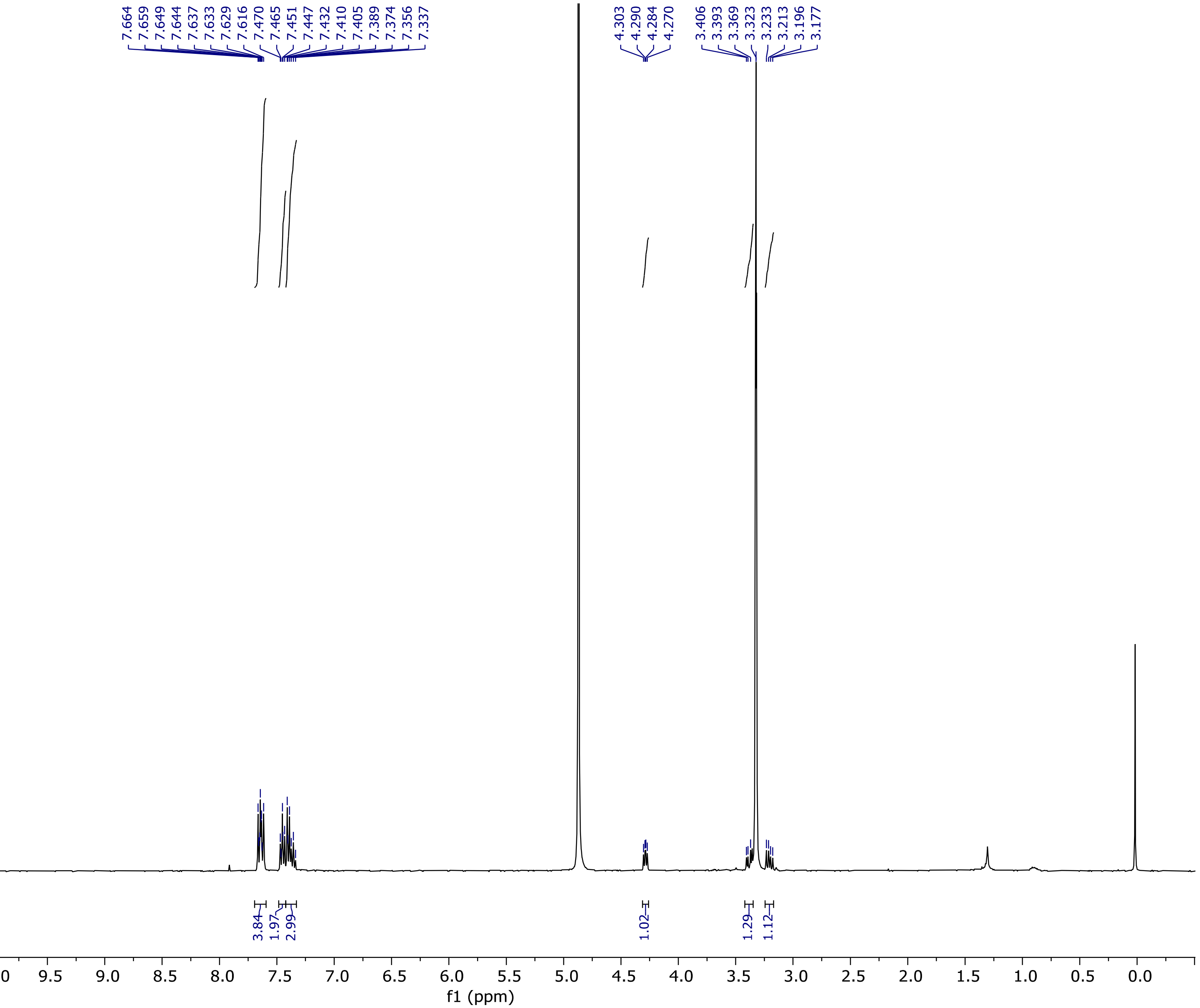

$^{13}C\{^1H\}$ NMR (101 MHz, CD_3OD)

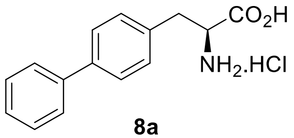

8a

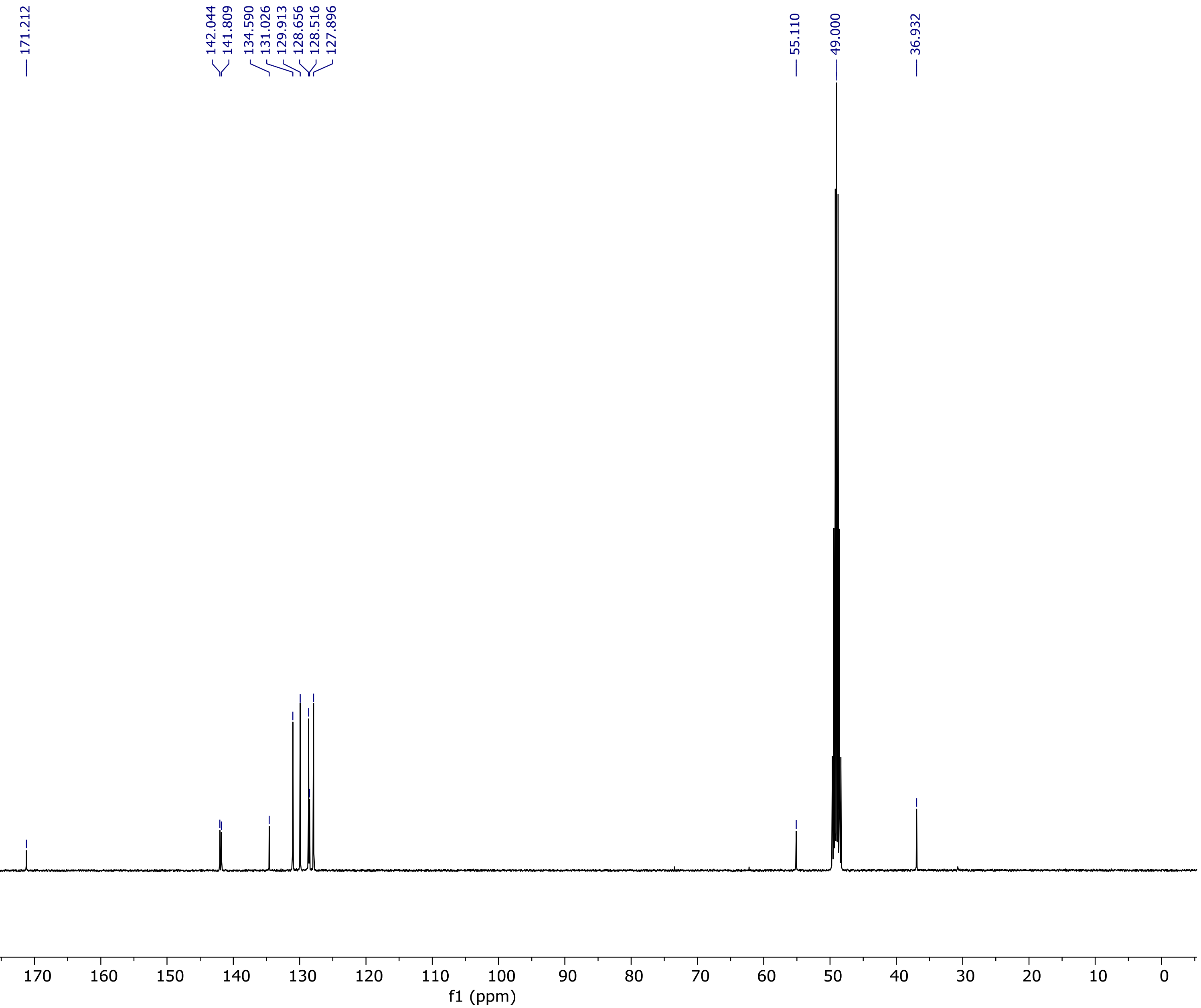

^{1}H NMR (400 MHz, CD_3OD)

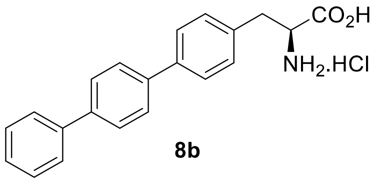

8b

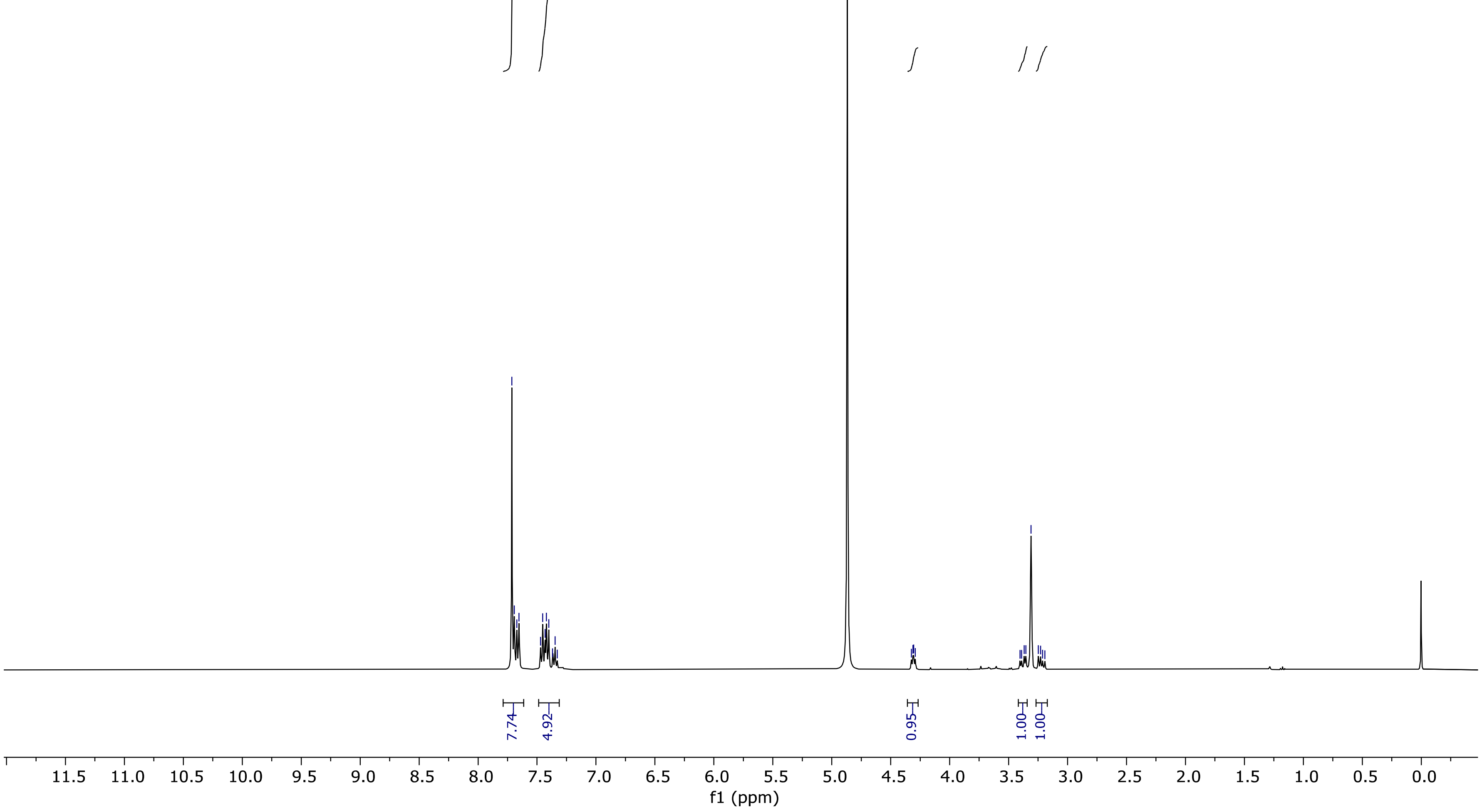

$^{13}C\{^{1}H\}$ NMR (101 MHz, CD_3OD)

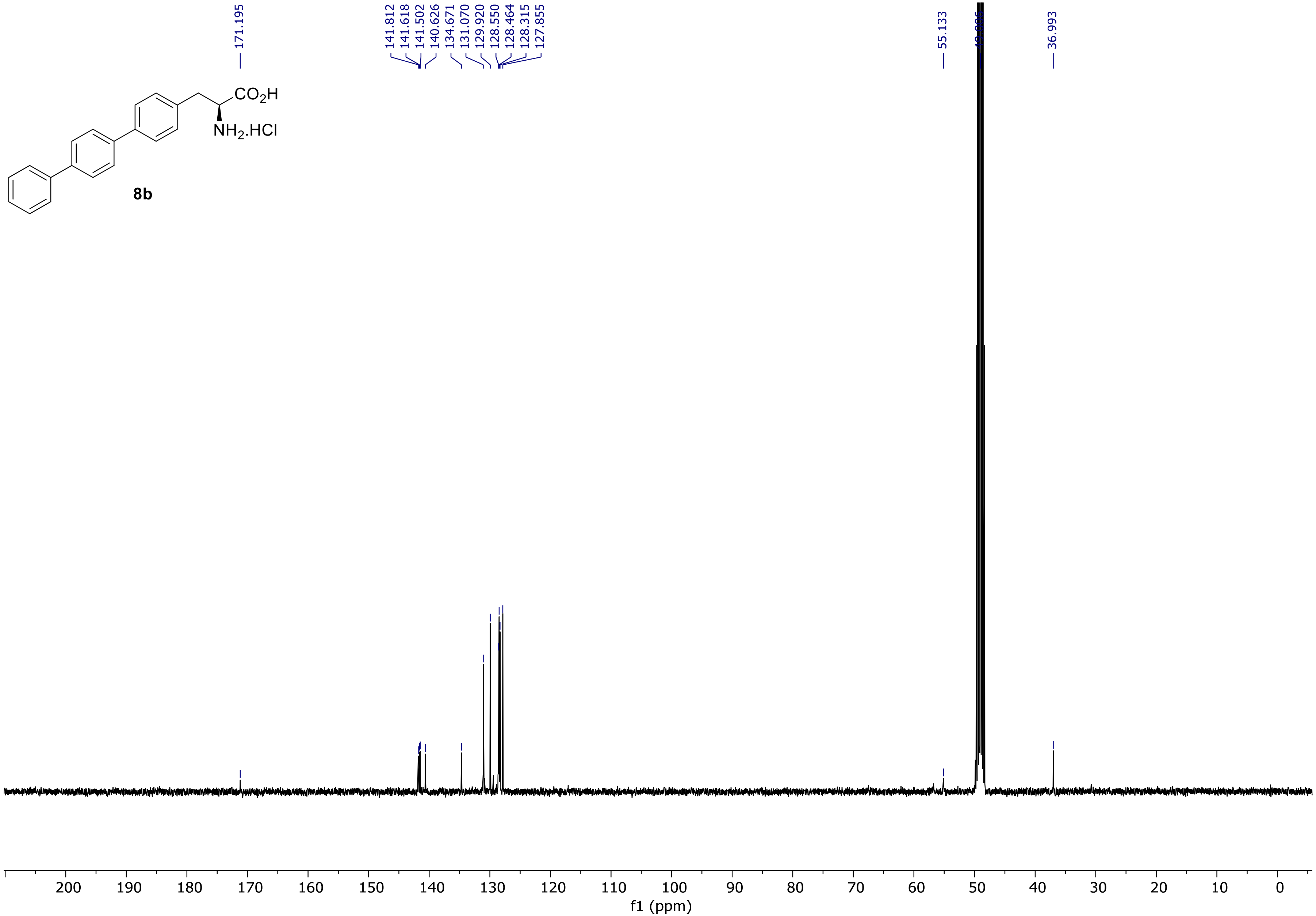

^{1}H NMR (400 MHz, CD_3OD)

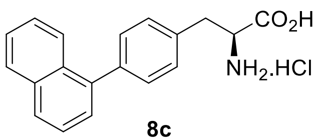

8c

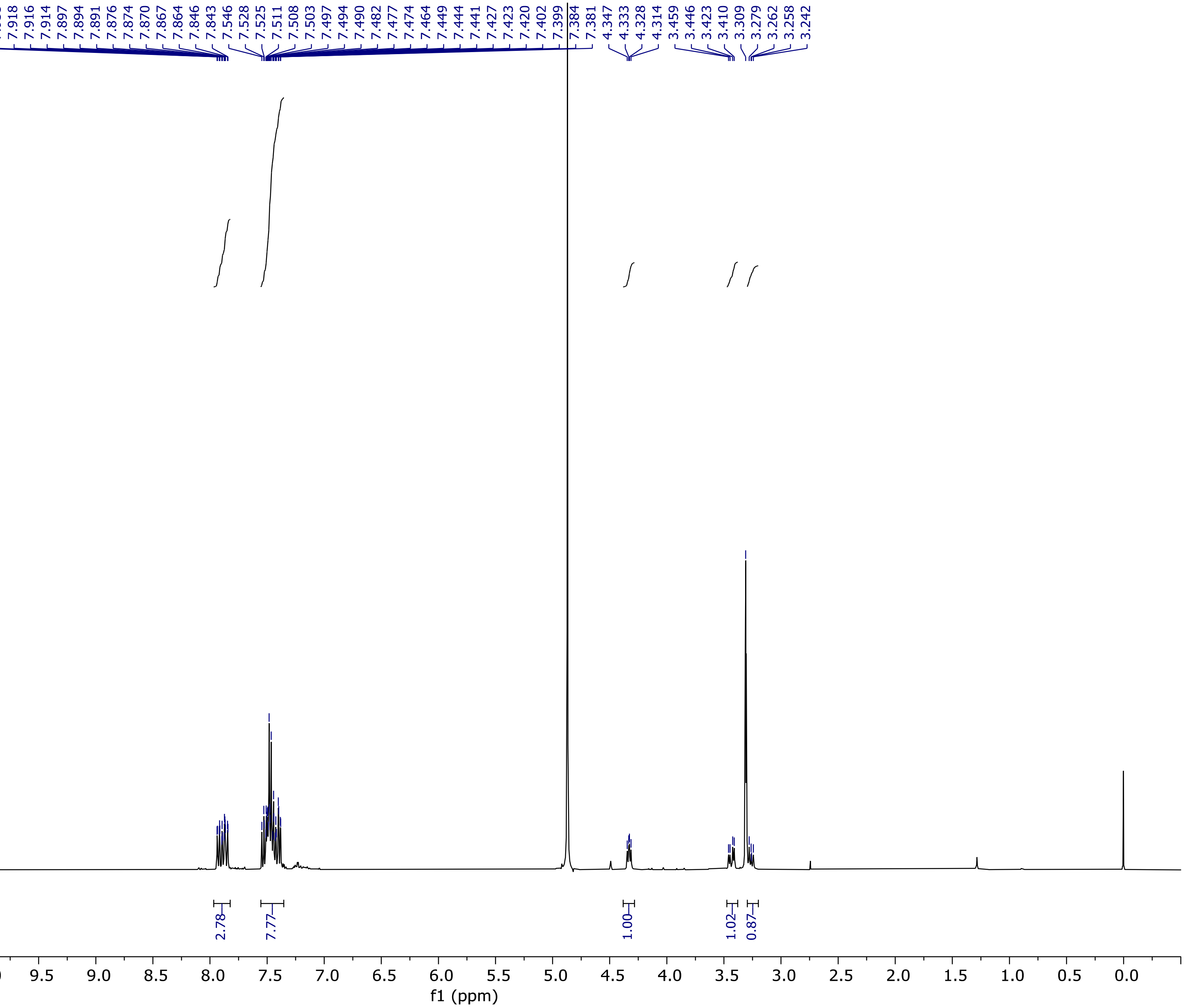

$^{13}C\{^1H\}$ NMR (101 MHz, CD_3OD)

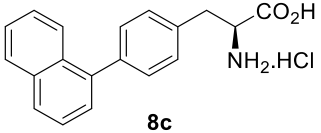

8c

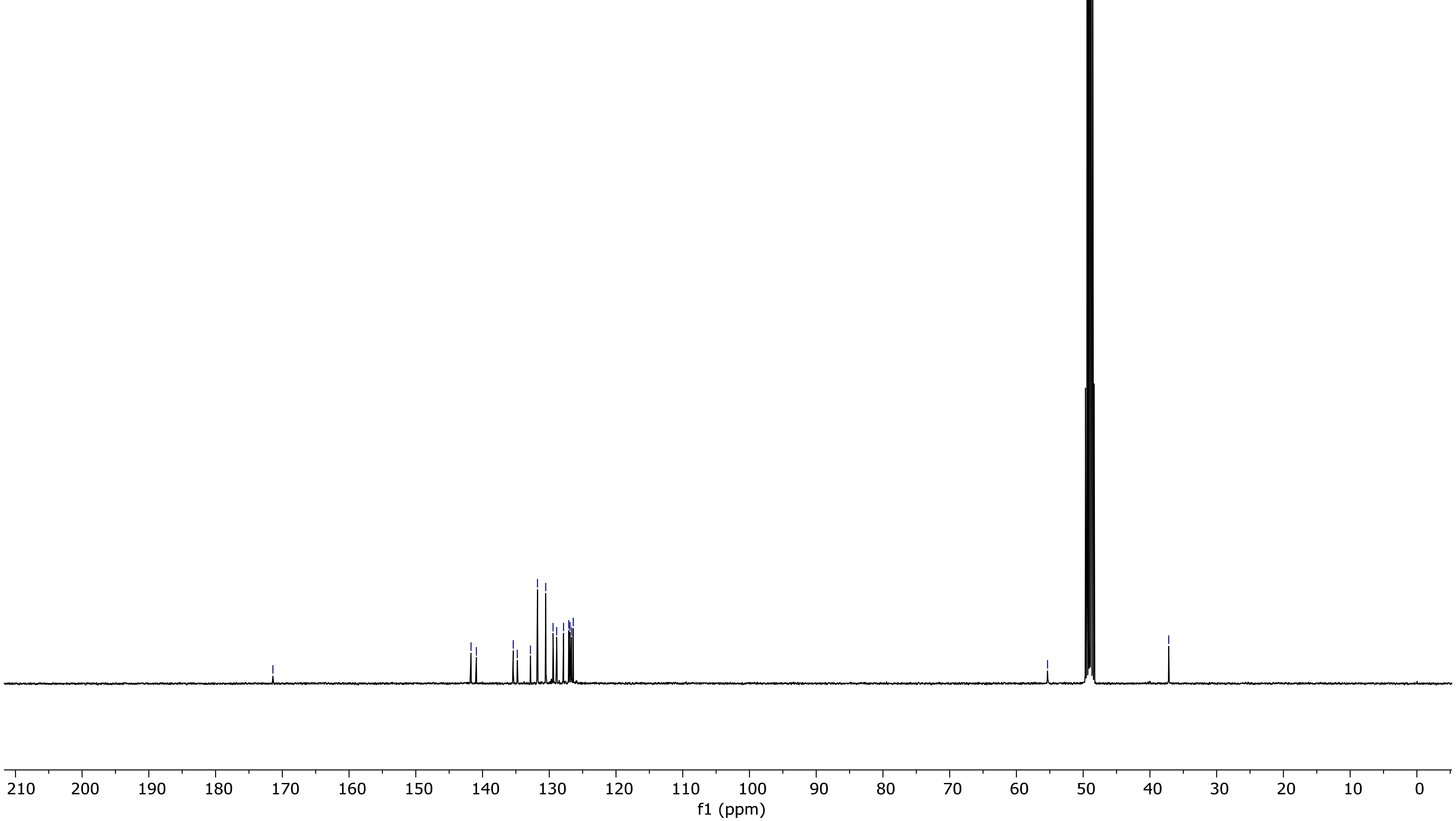

^{1}H NMR (400 MHz, CD_3OD)

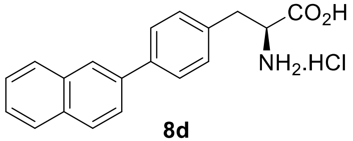

8d

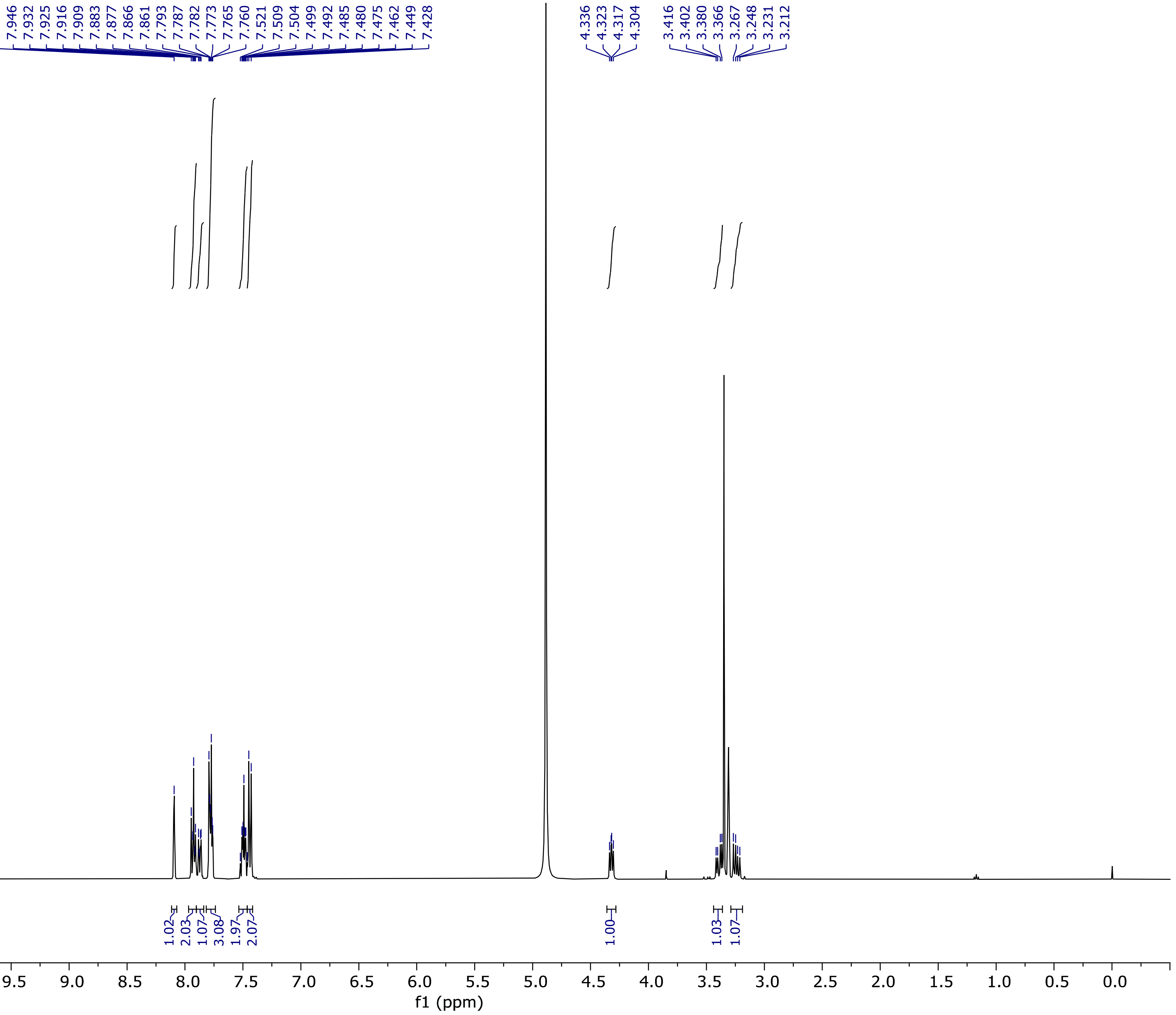

$^{13}C\{^1H\}$ NMR (101 MHz, CD_3OD)

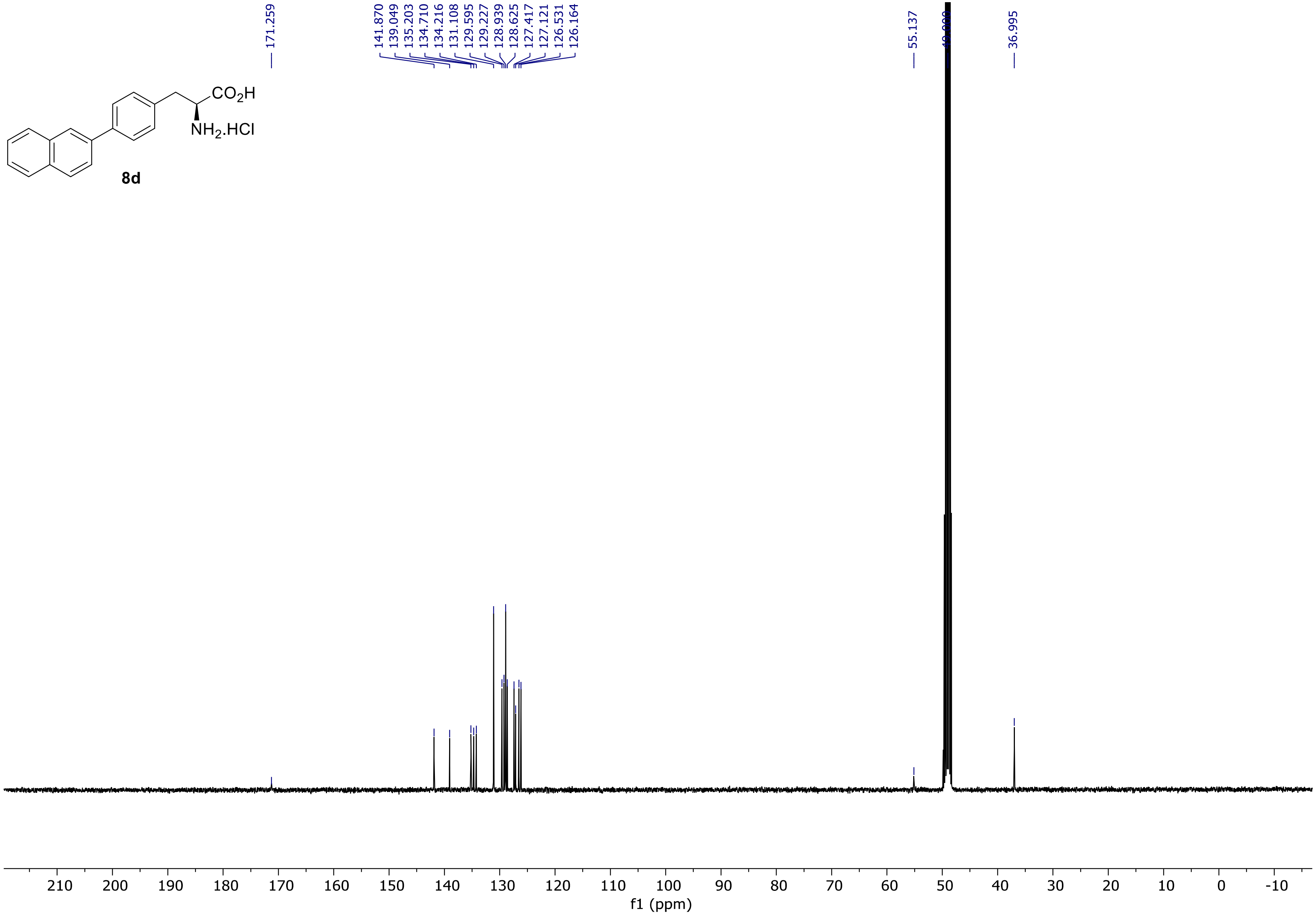

^{1}H NMR (400 MHz, CD_3OD)

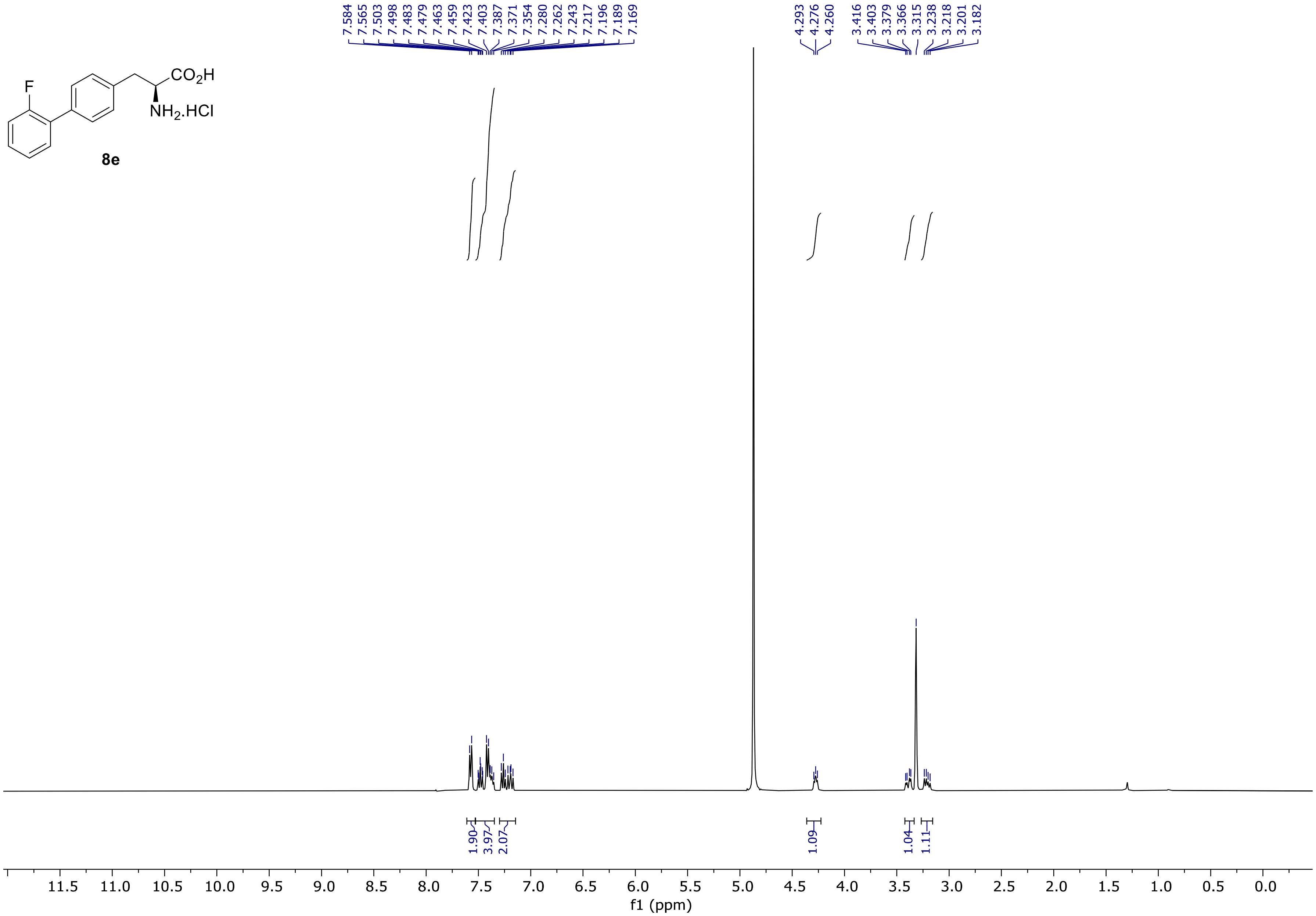

$^{13}C\{^{1}H\}$ NMR (101 MHz, CD_3OD)

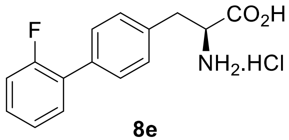

8e

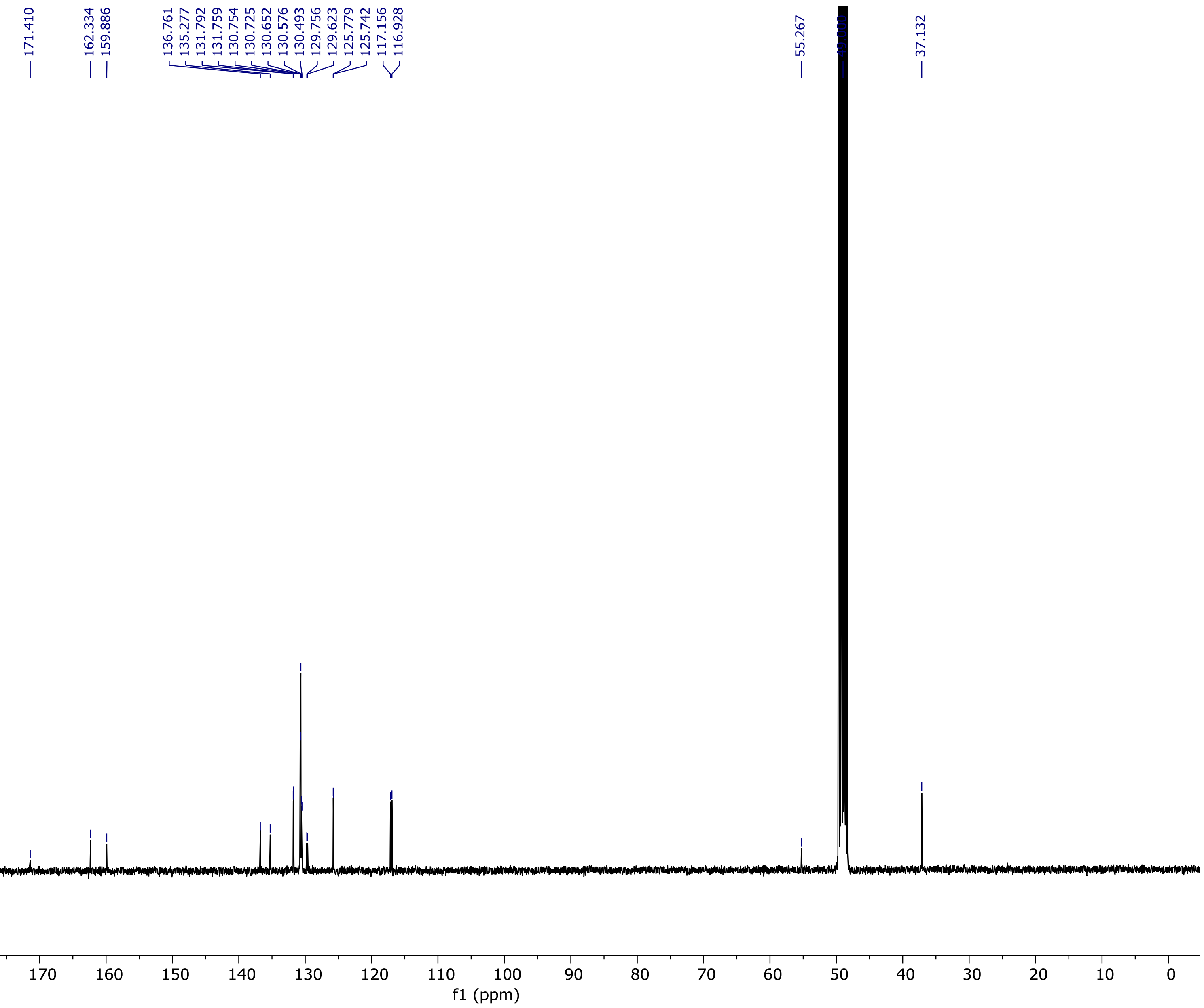

^{1}H NMR (400 MHz, CD_3OD)

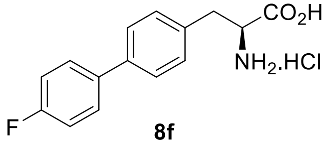

8f

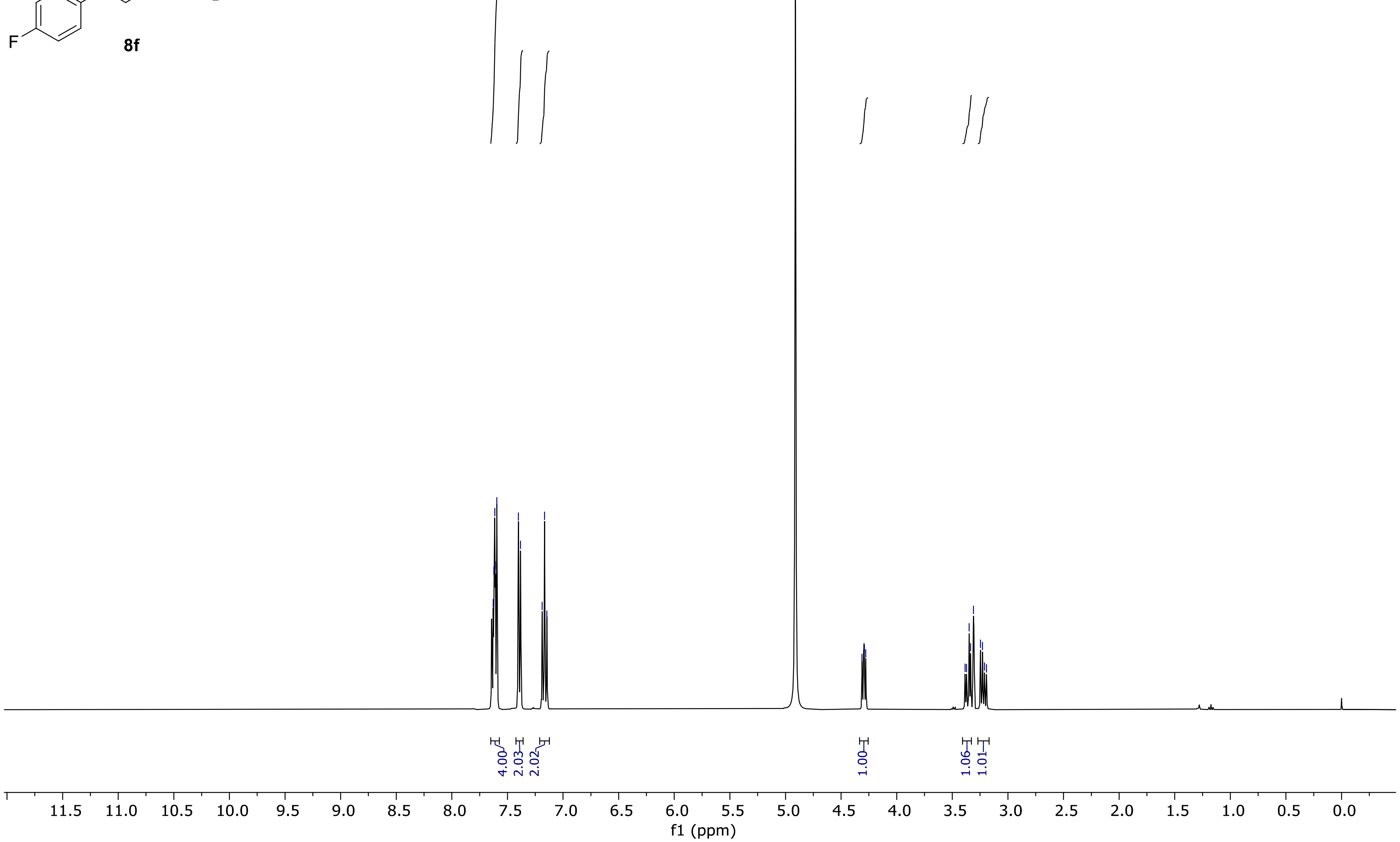

$^{13}C\{^{1}H\}$ NMR (101 MHz, CD_3OD)

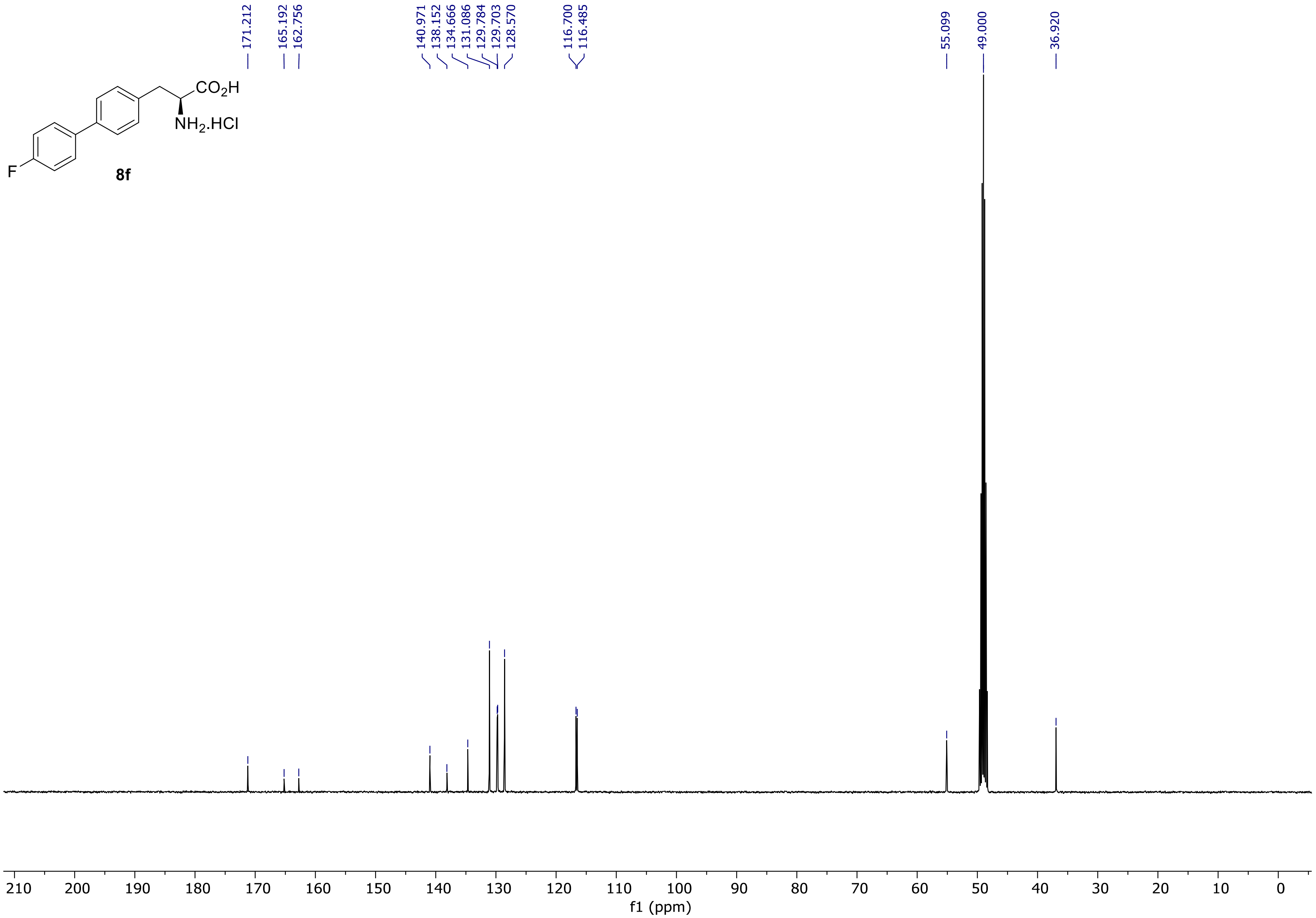

^{1}H NMR (400 MHz, CD_3OD)

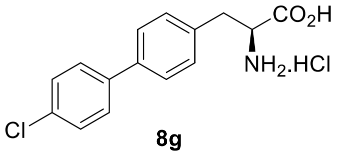

8g

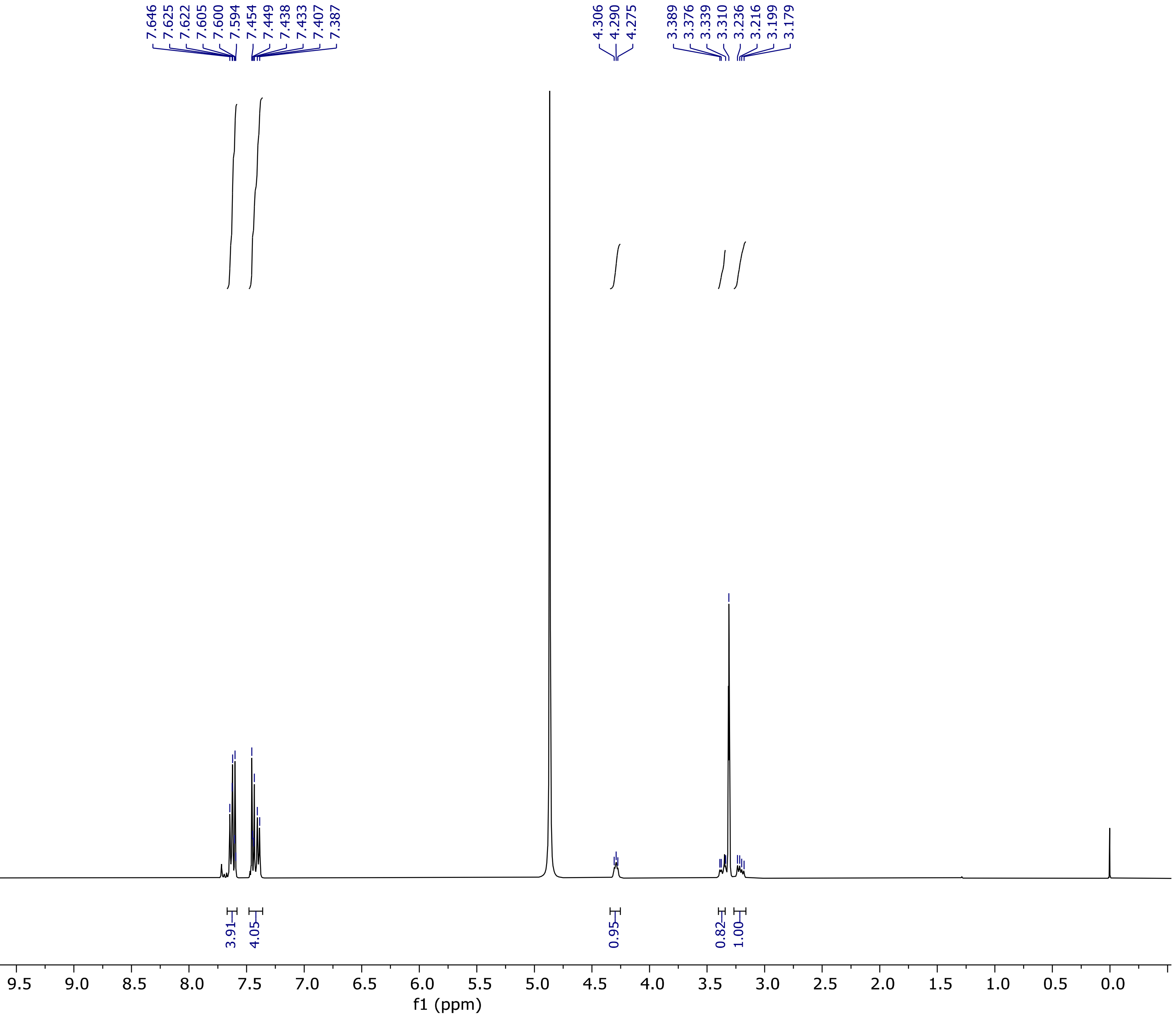

$^{13}C\{^1H\}$ NMR (101 MHz, CD_3OD)

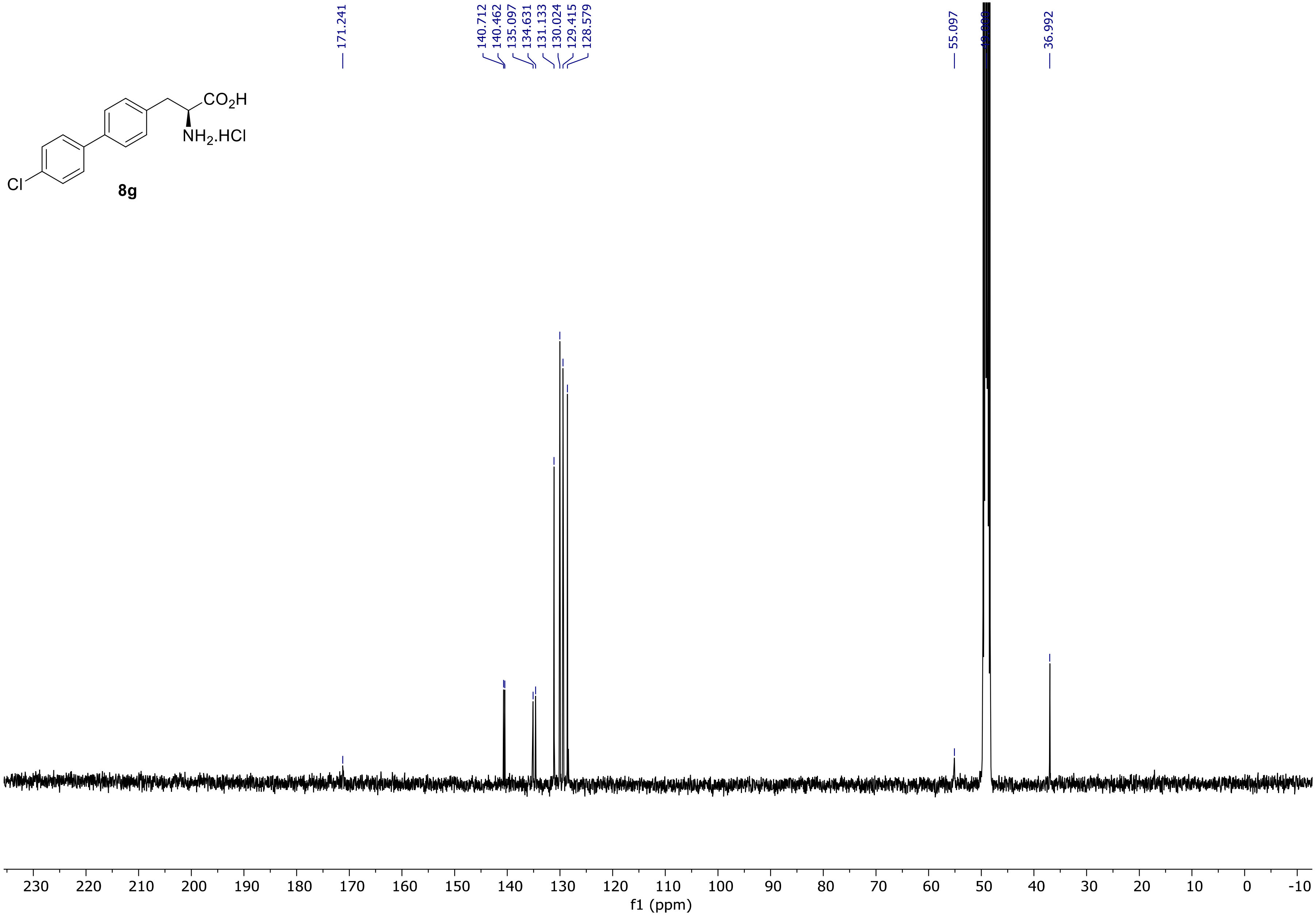

^{1}H NMR (400 MHz, CD_3OD)

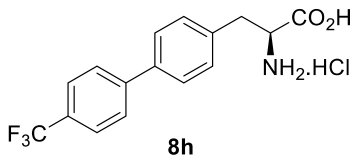

8h

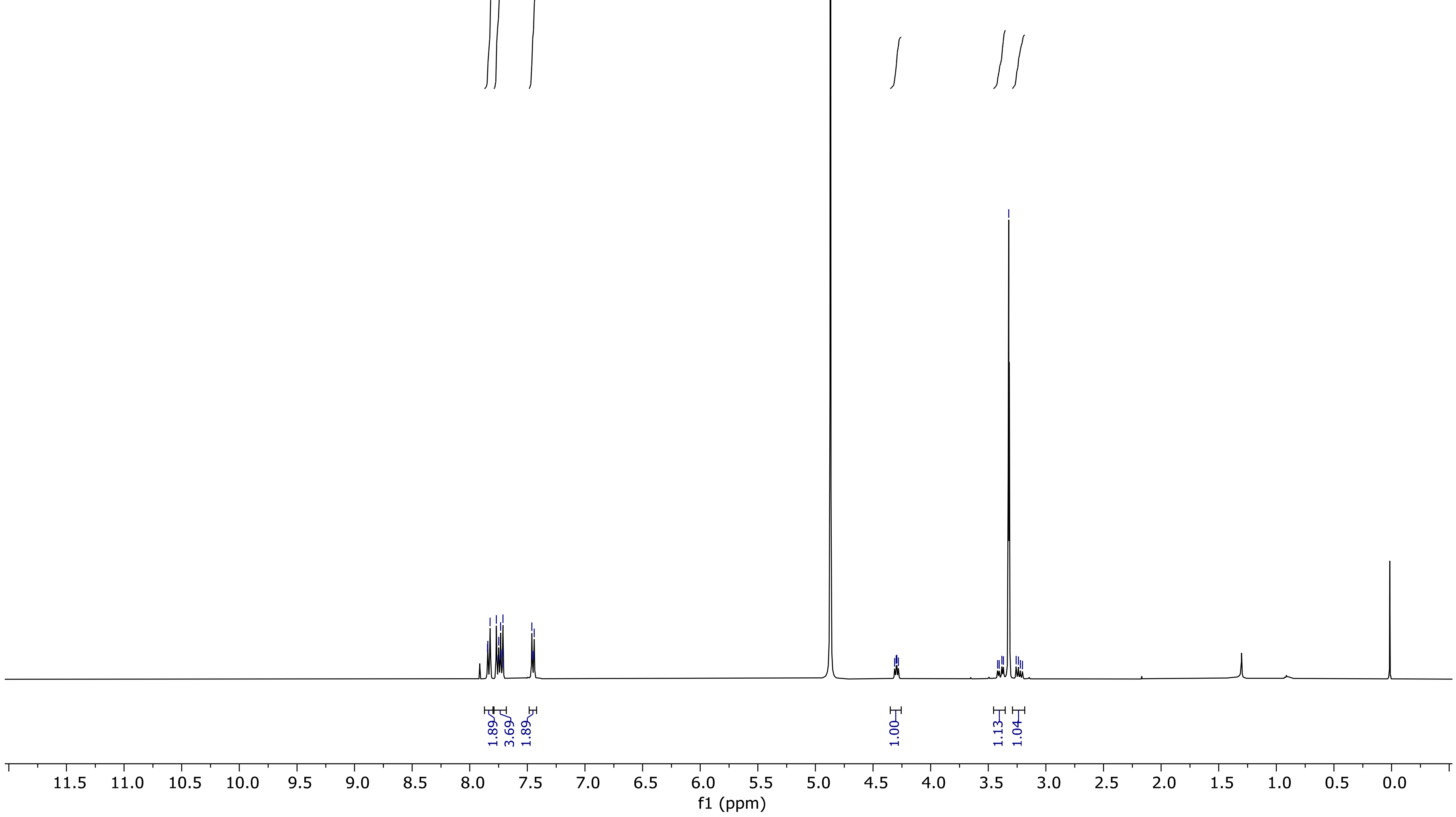

$^{13}C\{^1H\}$ NMR (101 MHz, CD_3OD)

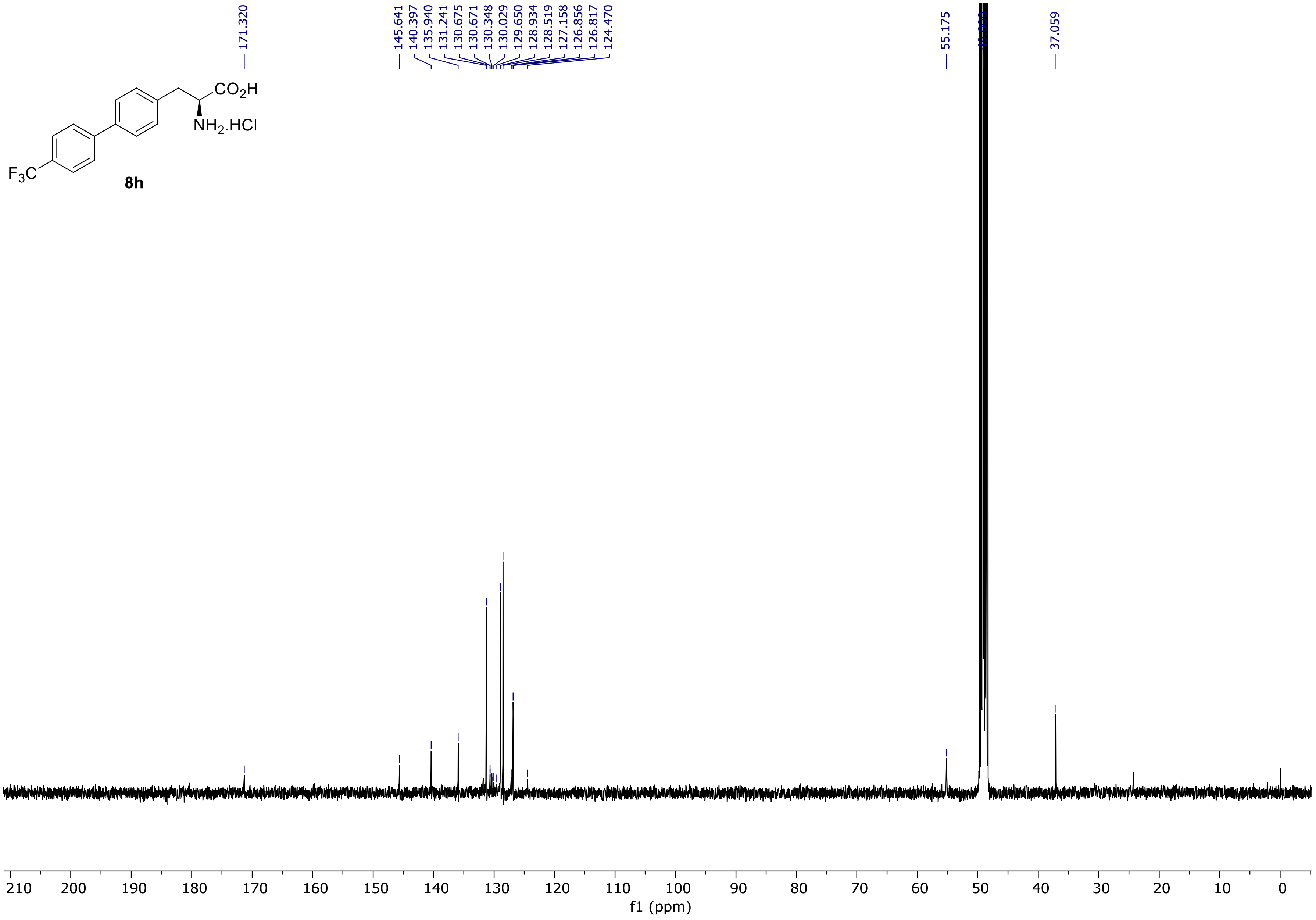

^{1}H NMR (400 MHz, CD_3OD)

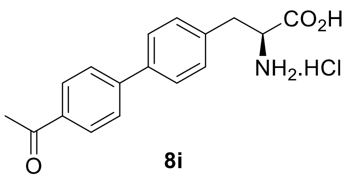
8i

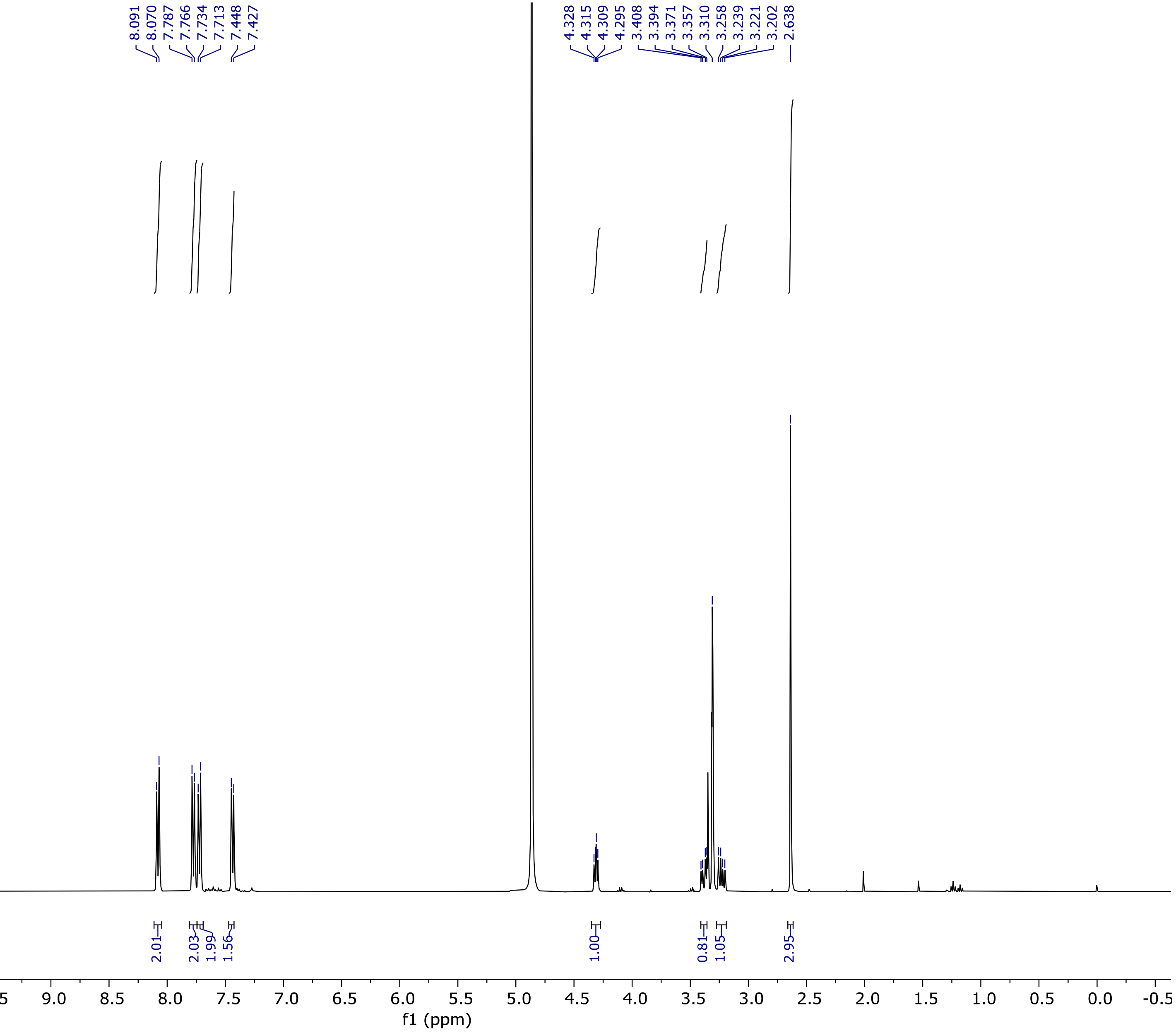

$^{13}C\{^1H\}$ NMR (101 MHz, CD_3OD)

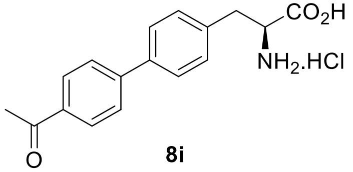

8i

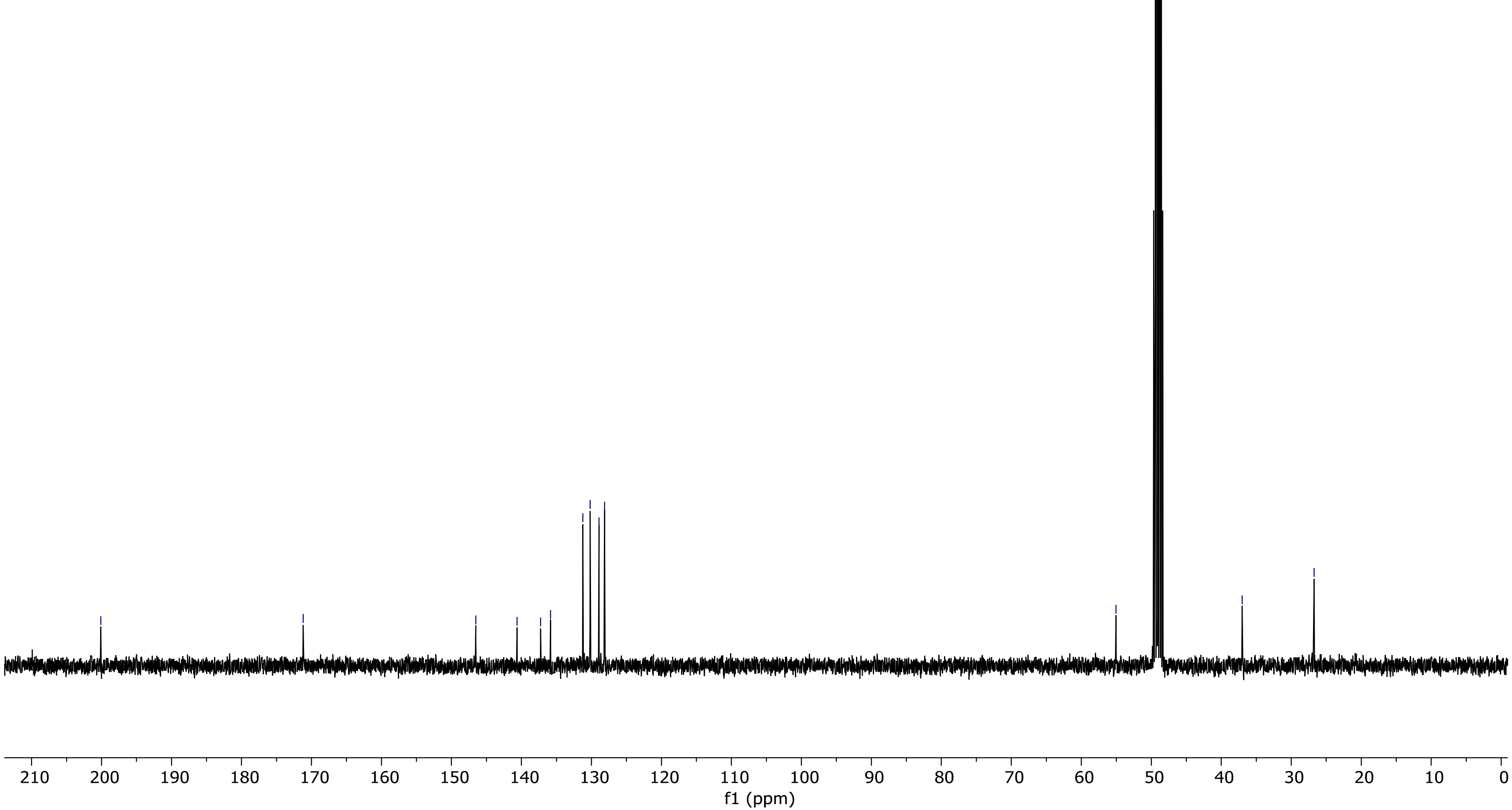

^{1}H NMR (400 MHz, CD_3OD)

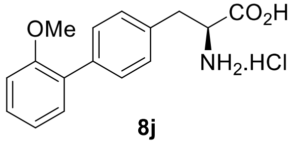

8j

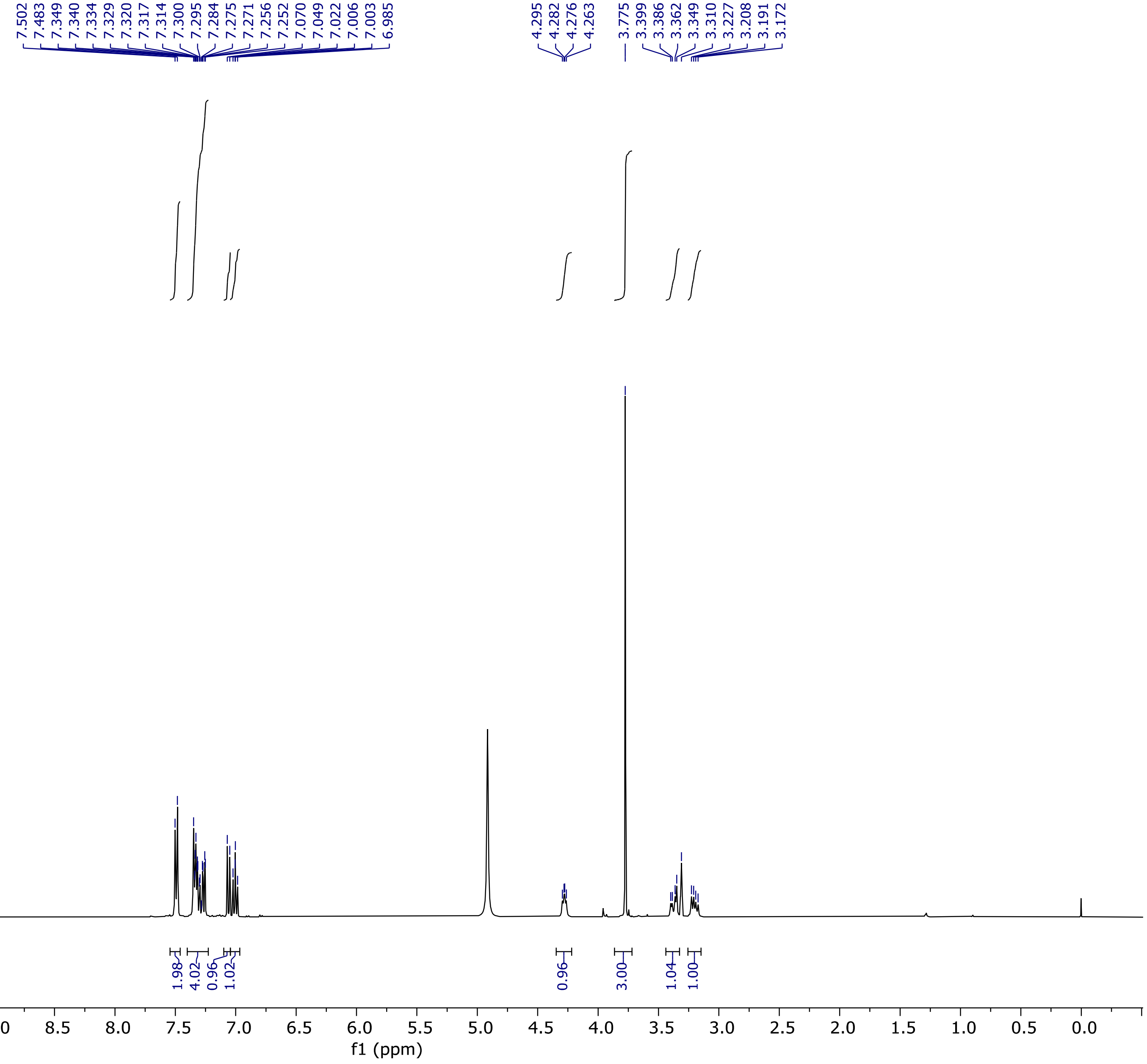

$^{13}C\{^1H\}$ NMR (101 MHz, CD_3OD)

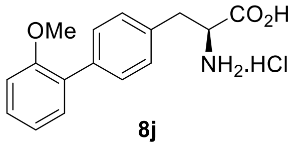

8j

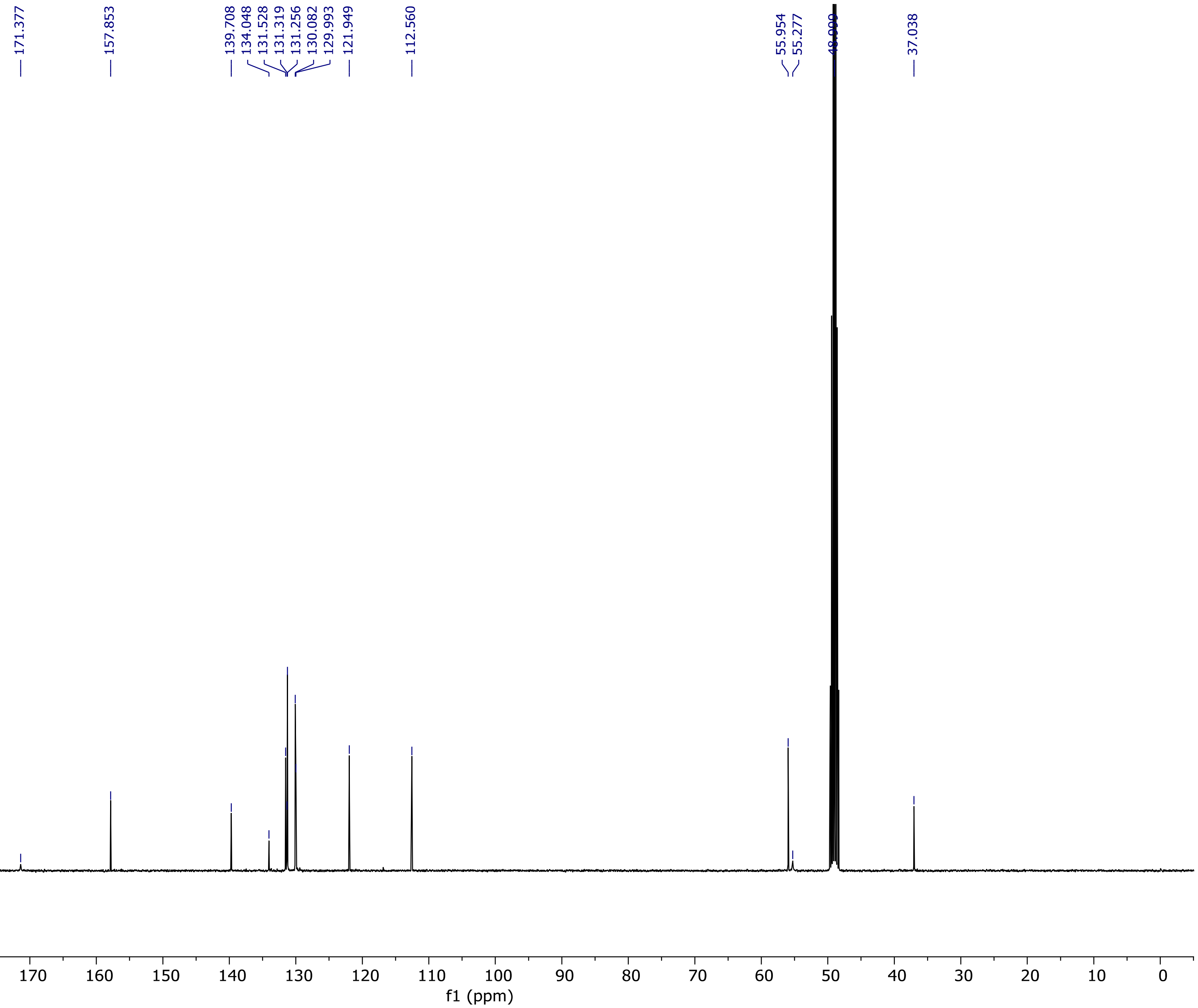

^{1}H NMR (400 MHz, CD_3OD)

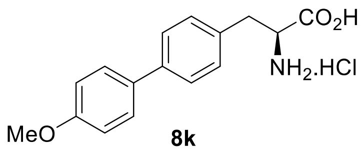

8k

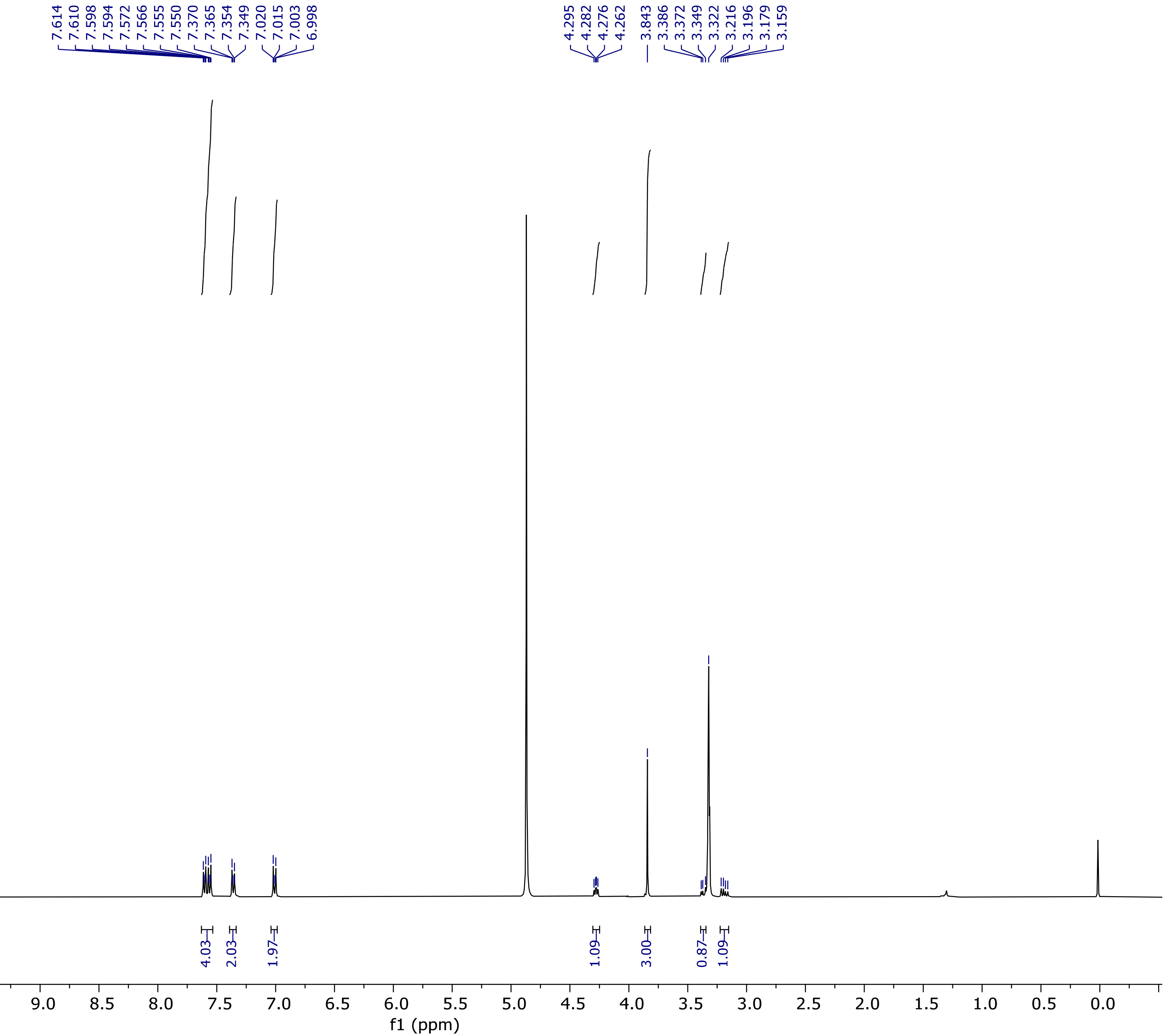

$^{13}C\{^{1}H\}$ NMR (101 MHz, CD_3OD)

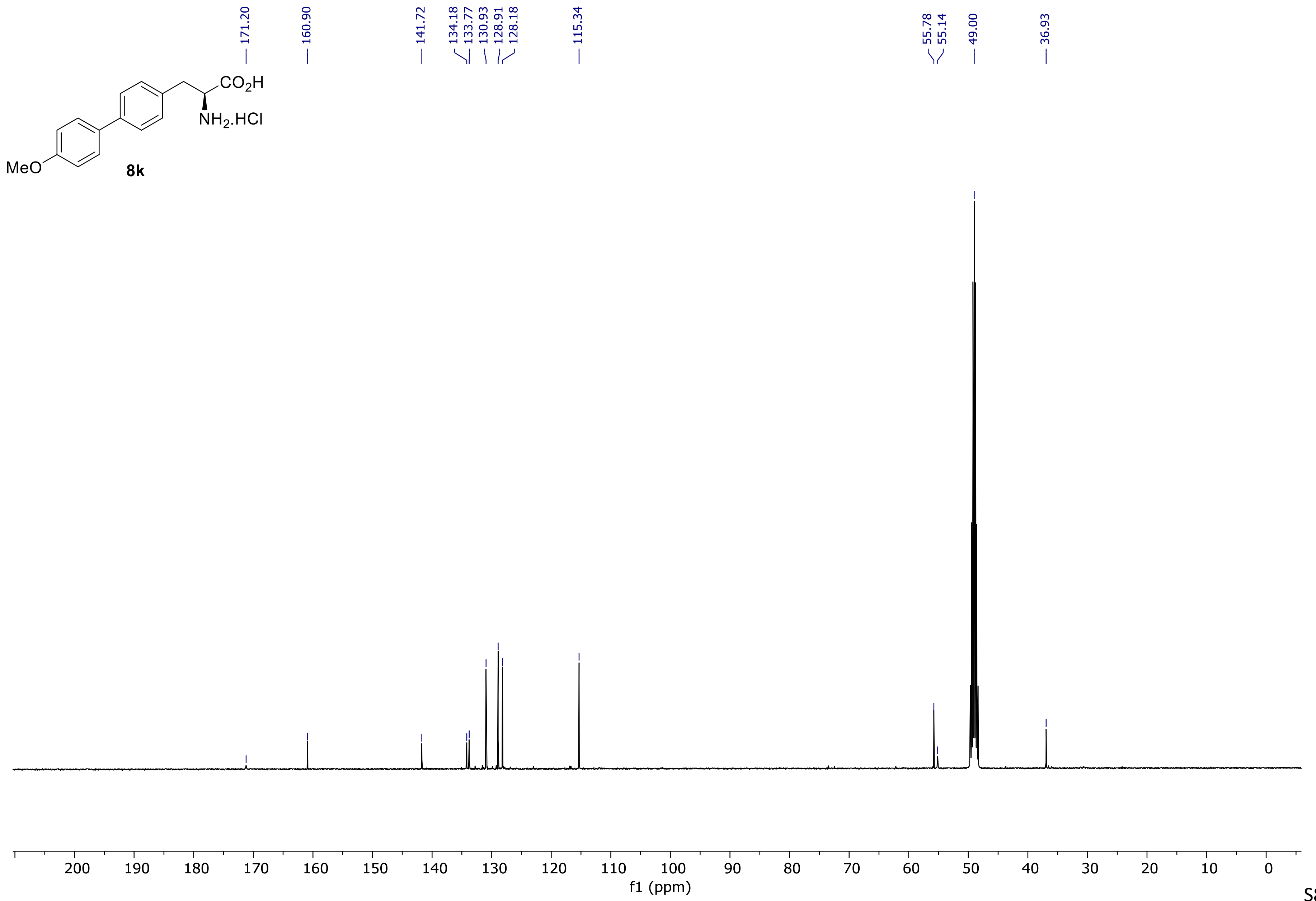

^{1}H NMR (400 MHz, CD_3OD)

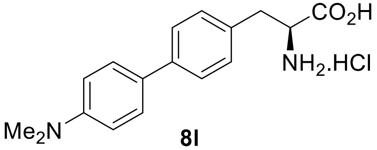

8l

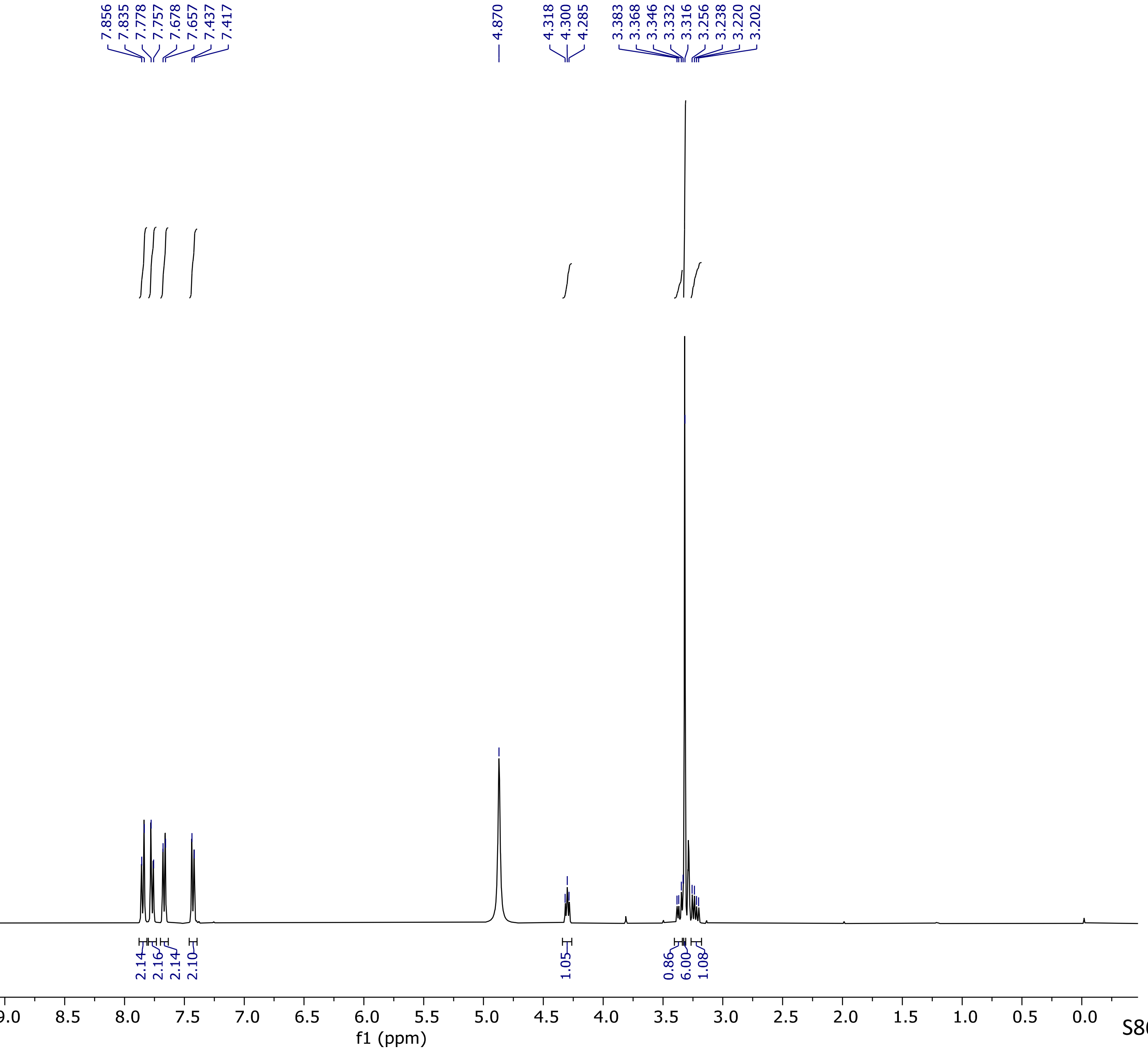

$^{13}C\{^{1}H\}$ NMR (101 MHz, CD_3OD)

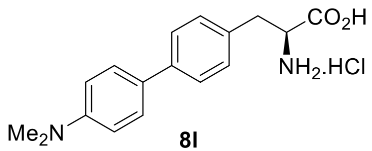

8l

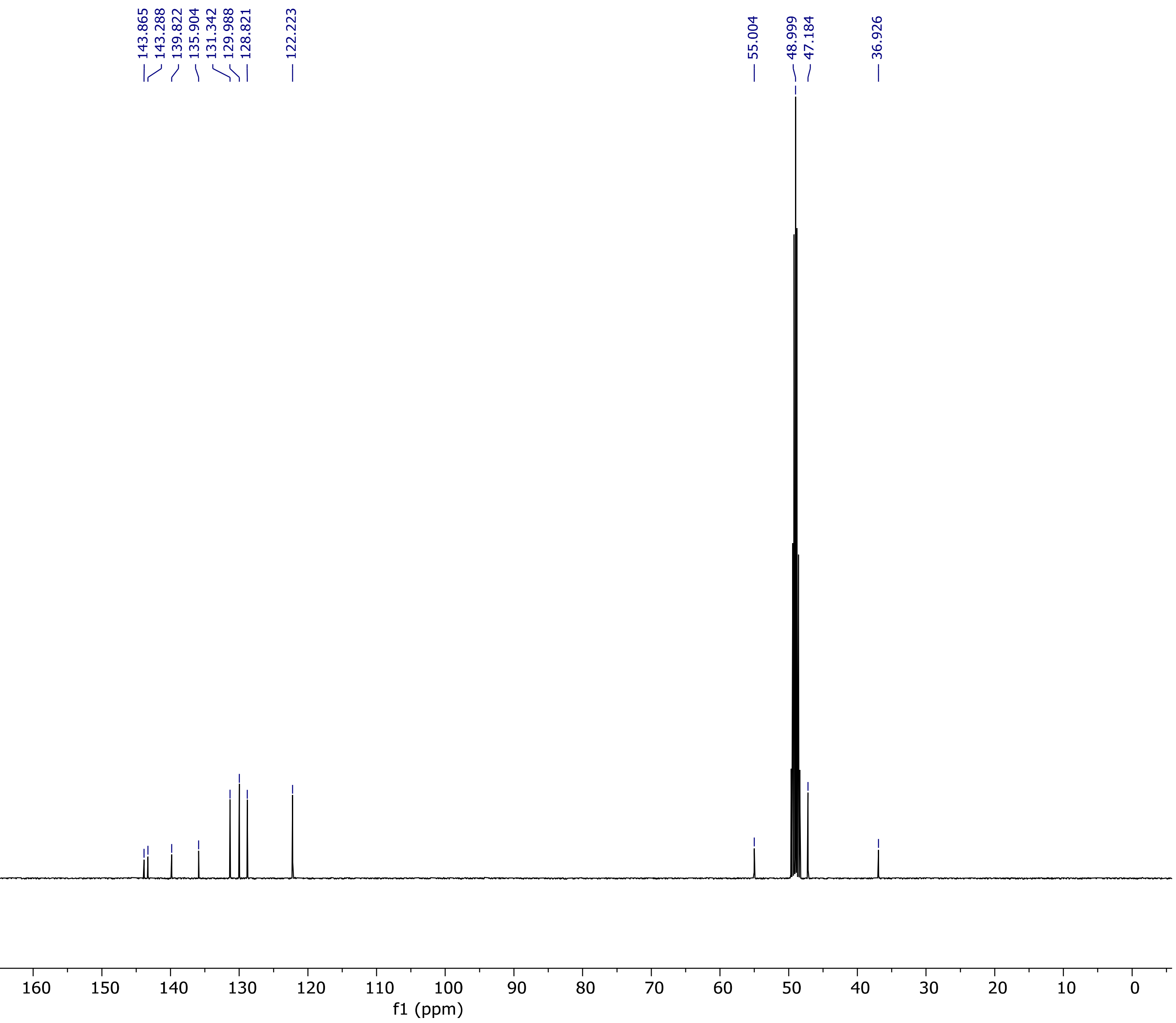

^{1}H NMR (400 MHz, CD_3OD)

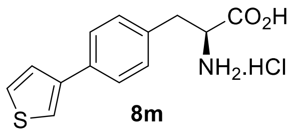

8m

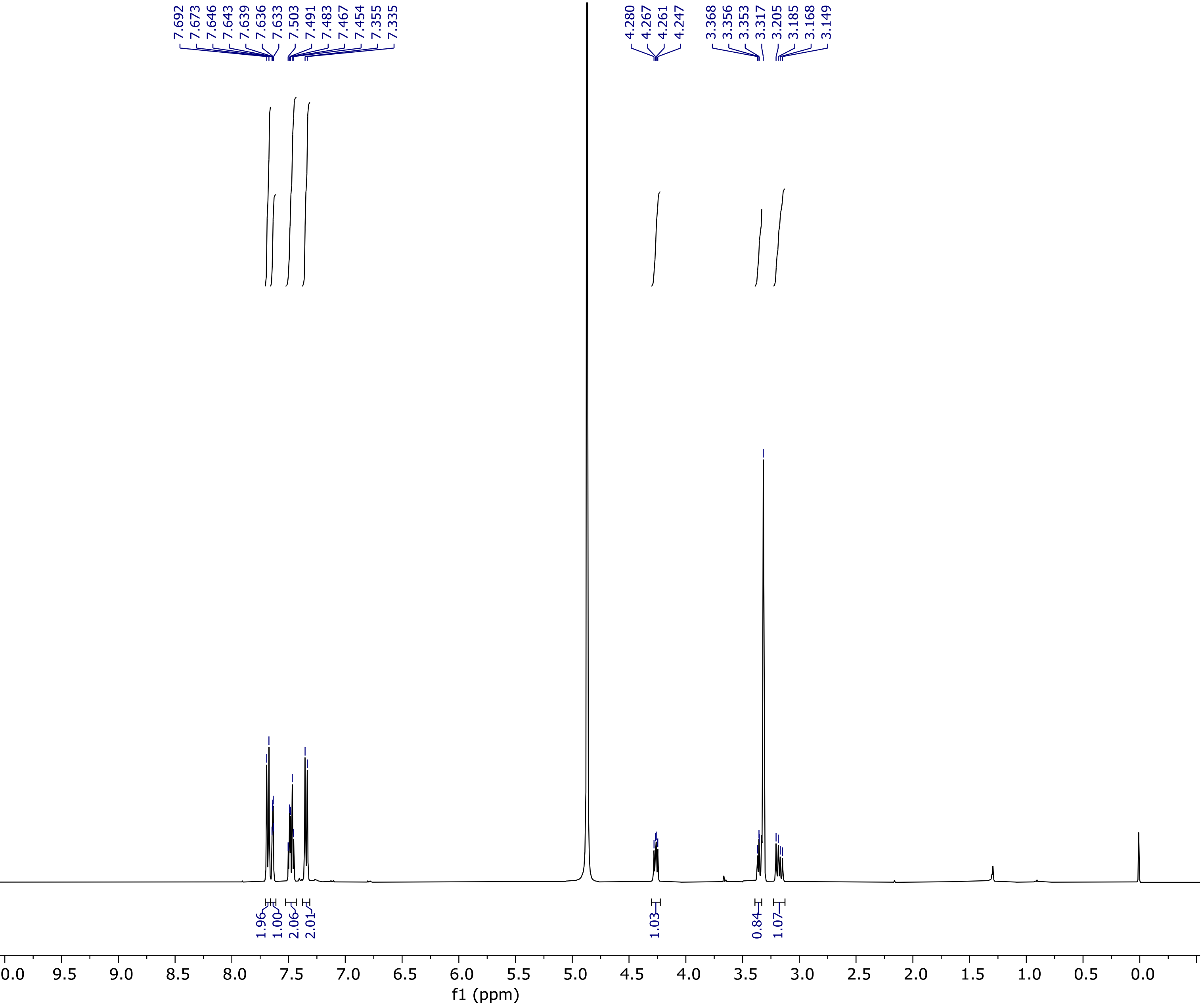

$^{13}C\{^1H\}$ NMR (101 MHz, CD_3OD)

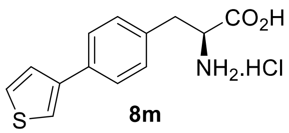

8m

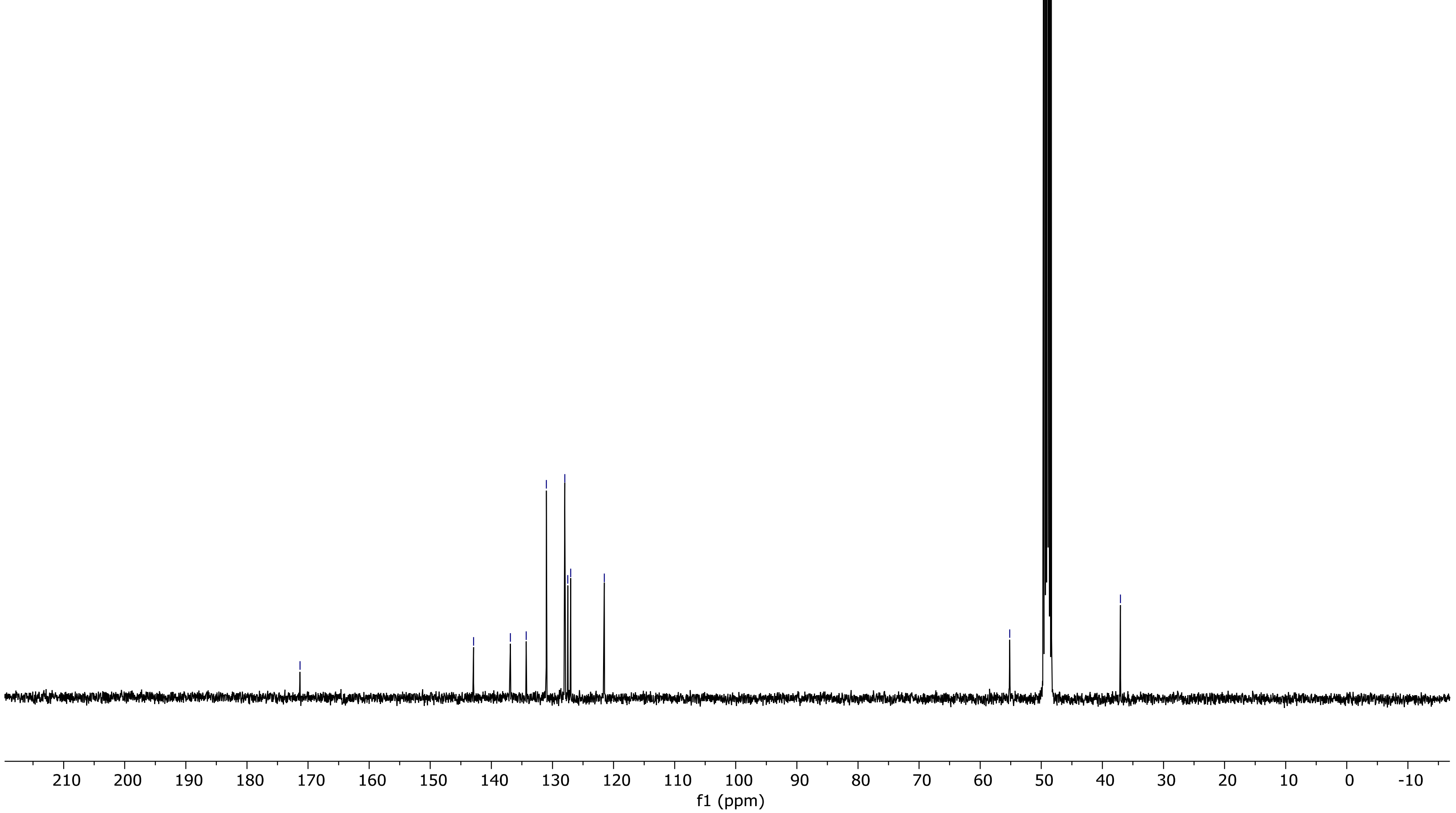

^{1}H NMR (400 MHz, DMSO-d_6)

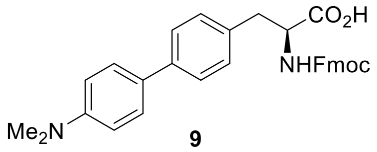

9

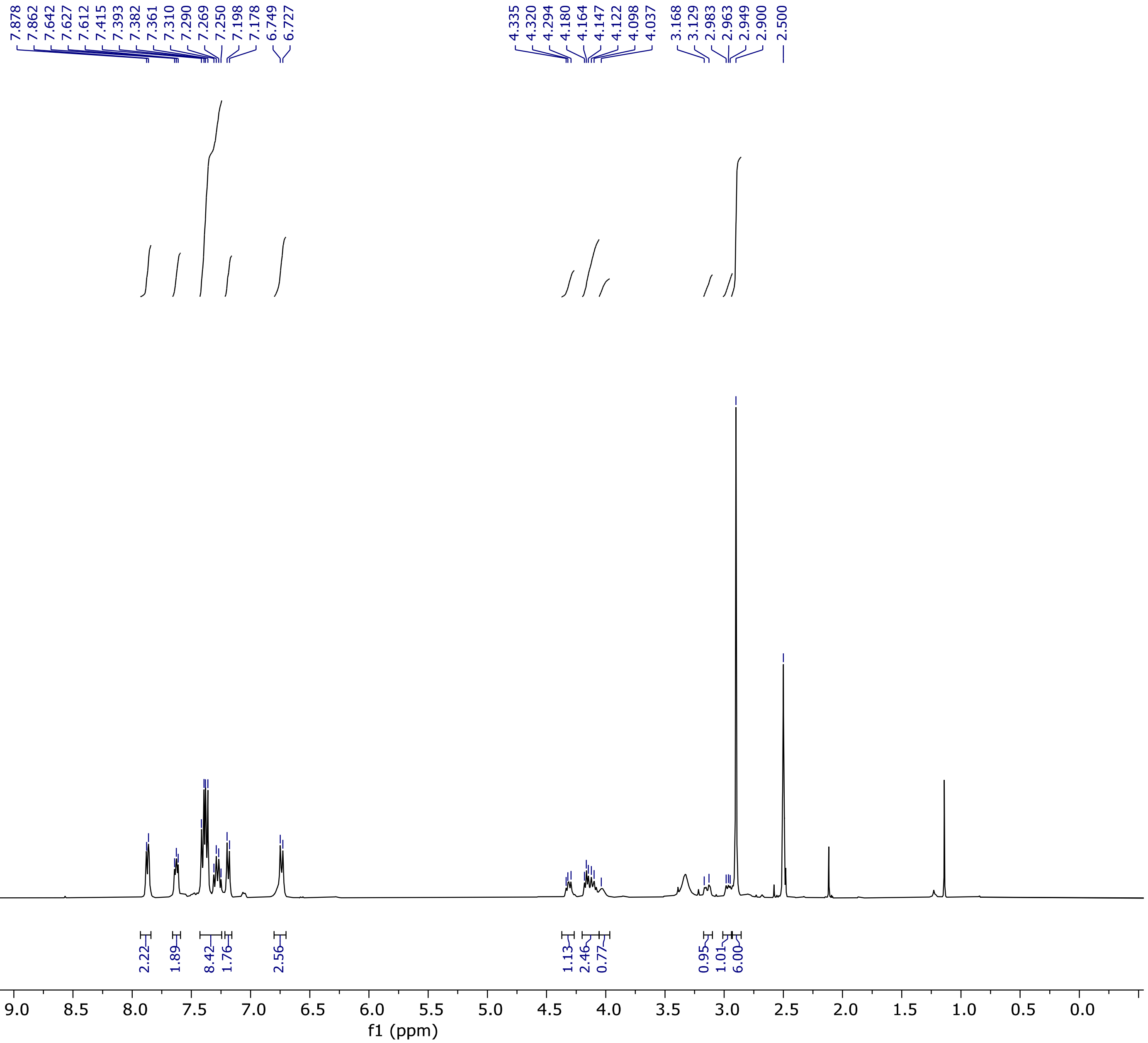

Supplement: SC-016-D5SC00020C-s001 [file SC-016-D5SC00020C-s001.pdf]
